# Unexpected loss of stereoselectivity in glycosylation reactions during the synthesis of chondroitin sulfate oligosaccharides

**DOI:** 10.3762/bjoc.15.14

**Published:** 2019-01-15

**Authors:** Teresa Mena-Barragán, José L de Paz, Pedro M Nieto

**Affiliations:** 1 Glycosystems Laboratory, Instituto de Investigaciones Químicas (IIQ), cicCartuja, CSIC and Universidad de Sevilla, Americo Vespucio, 49, 41092 Sevilla, Spain

**Keywords:** carbohydrate chemistry, chondroitin sulfate, glycosylation, oligosaccharide synthesis, stereoselectivity

## Abstract

Here, we present an exploratory study on the fluorous-assisted synthesis of chondroitin sulfate (CS) oligosaccharides. Following this approach, a CS tetrasaccharide was prepared. However, in contrast to our previous results, a significant loss of β-selectivity was observed in [2 + 2] glycosylations involving *N*-trifluoroacetyl-protected D-galactosamine donors and D-glucuronic acid (GlcA) acceptors. These results, together with those obtained from experiments employing model monosaccharide building blocks, highlight the impact of the glycosyl acceptor structure on the stereoselectivity of glycosylation reactions. Our study provides useful data about the substitution pattern of GlcA units for the efficient synthesis of CS oligomers.

## Introduction

Chondroitin sulfate (CS) is a highly heterogeneous polysaccharide, constituted by the repetition of D-glucuronic acid (GlcA)-β(1→3)-*N*-acetyl-D-galactosamine (GalNAc)-β(1→4) disaccharides that may contain sulfate groups at different positions. This biopolymer plays a key role in biological events such as brain development and cancer [1]. The preparation of well-defined CS oligosaccharides is crucial to determine structure–activity relationships and elucidate the particular sugar sequences involved in the interactions between CS and target proteins that regulate these biological functions. For this reason, several approaches have been reported for the synthesis of these molecules [2–8]. For example, Jacquinet, Lopin-Bon and co-workers presented the preparation of CS oligomers with different sulfation motifs, starting from a single GlcA-GalNAc disaccharide precursor easily obtained by depolymerization of the natural product [9–13]. CS tetrasaccharides following the sequence GlcA-GalNAc have also been obtained by [2 + 2] coupling of *N*-trichloroacetyl protected disaccharide units [14–15]. On the other hand, Tamura’s group reported the synthesis of CS tetra-, hexa- and octasaccharides, displaying biologically relevant sulfate distributions, by direct coupling between *N*-acetyl building blocks with the opposite sequence GalNAc-GlcA [16–19]. All of these routes involved the use of low reactive glucuronic acid moieties in glycosylation reactions. Very recently, a postglycosylation–oxidation strategy was applied to the synthesis of CS type E oligosaccharides in order to improve the glycosylation efficiency [20–21]. In this approach, glucose instead of GlcA residues were employed in the oligosaccharide assembly and final oxidation and derivatization was carried out at the oligomer stage. Syndecan-1 glycopeptide containing a CS type A sequence was also obtained by using a postglycosylation–oxidation strategy and thioglycosides as glycosyl donors [22].

Despite all these recent advances in the synthesis of CS oligosaccharides, the classical solution-phase preparation of these molecules is hampered by the required iterative chromatographic purifications. Therefore, alternative methodologies are still demanded in order to facilitate and accelerate intermediate purifications [23–25]. In this context, Seeberger and co-workers developed an automated solid-phase synthesis of CS derivatives using a photolabile linker [26].

Here, we describe the exploration of a fluorous-assisted approach for the synthesis of CS oligosaccharides. The use of a fluorous tag allows the rapid isolation of fluorinated intermediates by performing a simple extraction from fluorous silica gel [27–32]. Moreover, this method maintains the advantages of solution-phase synthesis, such as easy reaction monitoring by standard techniques and low consumption of donor building blocks to complete glycosylations. During the course of this investigation, unexpected stereochemical outcomes were observed in glycosylations involving glycosyl donors containing an *N*-trifluroacetyl participating group at position 2. Our results provide data for the correct design of CS building blocks and highlight the influence of glycosyl acceptor reactivity on the stereoselectivity of glycosylation reactions even in the presence of 2-participating groups [33].

## Results and Discussion

**Scheme 1:**
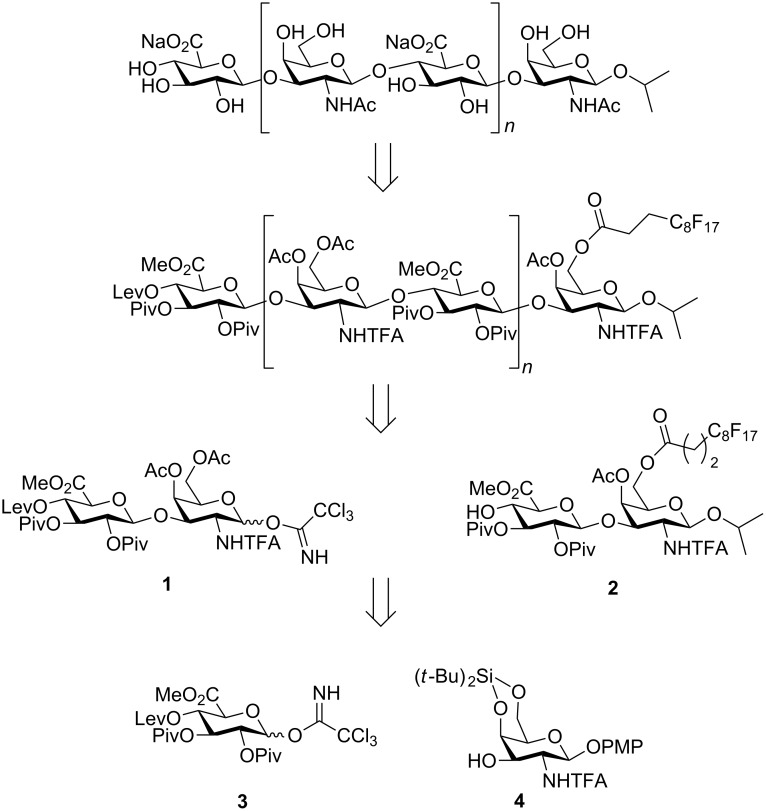
Retrosynthetic analysis for the preparation of CS oligosaccharides. Lev = levulinyl; Piv = pivaloyl; PMP = 4-methoxyphenyl.

We envisioned the synthetic approach shown in Scheme 1 for the preparation of CS oligosaccharides. Final molecules would be prepared from fully protected precursors by extensive basic hydrolysis followed by selective *N*-acetylation. The CS chains would be generated by iterative coupling between the disaccharide glycosyl trichloroacetimidate [34] **1** and the corresponding glycosyl acceptor, such as **2**. Subsequent delevulination would release the 4-OH group at the non-reducing end for next glycosylation. The C_8_F_17_ tail at position 6 of the galactosamine reducing end unit would allow us to purify the reaction intermediates by simple fluorous solid-phase extraction (F-SPE). Interestingly, the fluorous tag would be introduced by acylation using commercially available heptadecafluoroundecanoyl chloride and its deprotection would not involve any additional step at the end of the synthesis. *N*-Trifluoroacetyl (*N*-TFA)-protected disaccharides **1** and **2** would be prepared from known building blocks **3** [35] and **4** [36] using D-glucurono-6,3-lactone and D-galactosamine hydrochloride as starting materials, respectively. The *N*-TFA group [37–41] is an adequeate choice for 2-amino protection of GalNAc moieties because it leads to the selective formation of the desired 1,2-*trans* glycosidic linkages and can be easily removed at the end of the synthesis [7–8,36,42]. Moreover, ^19^F NMR experiments can be employed to assist in the analysis and monitoring of reactions involving *N*-TFA building blocks.

**Scheme 2:**
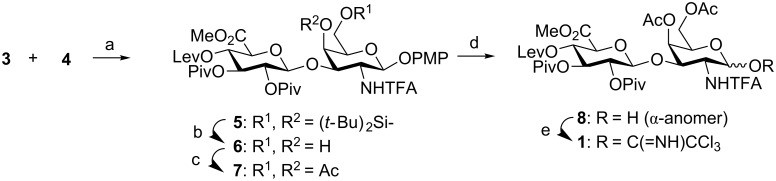
Reagents and conditions: a) TMSOTf, CH_2_Cl_2_, 0 °C, 30 min, 97%; b) (HF)*_n_*·Py, THF, 0 °C, 20 h, 90%; c) Ac_2_O, Py, 24 h, 97%; d) CAN, CH_2_Cl_2_/CH_3_CN/H_2_O, 0 °C, 1 h, 99%; e) Cl_3_CCN, DBU, CH_2_Cl_2_, 9 h, 98%. CAN = ceric(IV) ammonium nitrate.

First, we describe the preparation of disaccharide donor **1** and acceptor **2**. We have previously shown that the glycosylation between a 3-*O*-benzyl-2,4-di-*O*-acylated GlcA trichloroacetimidate and the GalNAc acceptor **4** afforded the corresponding β-disaccharide in excellent yield [36]. The presence of the 4,6-*O*-di-*tert*-butylsilylene group was the key for the high efficiency of this coupling. Similarly, condensation between peracylated GlcA donor **3** and **4** gave disaccharide **5** in excellent 97% yield (Scheme 2). The cyclic silylene group was then replaced by two acetyl groups because it is well-known that cyclic protecting groups at positions 4 and 6 of GalNAc units favor the formation of 1,2-*cis* glycosidic bonds, even in the presence of 2-participating groups [43–44]. Treatment with (HF)*_n_*·Py complex in THF followed by standard acetylation provided compound **7**. This derivative displayed a suitable protecting group distribution for the selective formation of the β(1→4) linkage in the [2 + 2] coupling. Cleavage of the anomeric 4-methoxyphenyl group followed by treatment with trichloroacetonitrile and DBU gave glycosyl donor **1** in high yield.

**Scheme 3:**
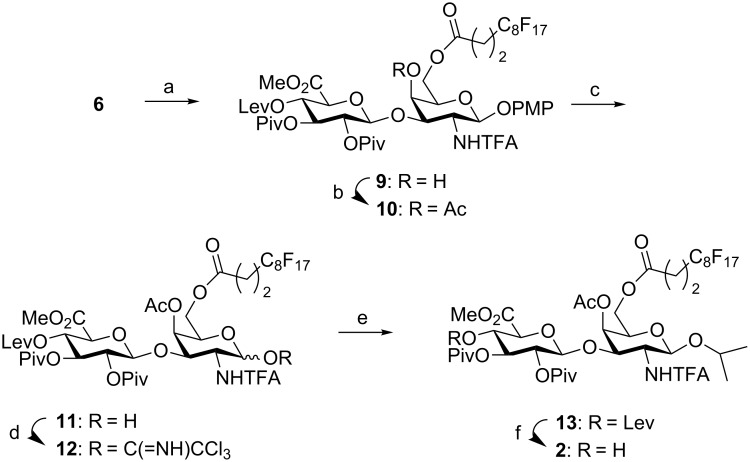
Reagents and conditions: a) C_8_F_17_CH_2_CH_2_COCl, Et_3_N, DMAP, DMF/CH_2_Cl_2_, 0 °C to rt, 6 h, 70%; b) Ac_2_O, Py, 24 h, 94%; c) CAN, CH_2_Cl_2_/CH_3_CN/H_2_O, 0 °C, 2 h, 54%; d) Cl_3_CCN, DBU, CH_2_Cl_2_, 6 h, 76%; e) 2-propanol, TMSOTf, CH_2_Cl_2_, 0 °C, 30 min, 65%; f) NH_2_NH_2_·H_2_O, Py/AcOH, CH_2_Cl_2_, 1 h, 94%.

For the synthesis of acceptor **2**, diol **6** was selectively acylated at position 6 using 1.1 equiv of heptadecafluoroundecanoyl chloride in the presence of Et_3_N and catalytic DMAP in a DMF/CH_2_Cl_2_ solvent mixture (Scheme 3). Acetylation of the 4-hydroxy group provided disaccharide **10** after F-SPE purification. We planned to employ the final CS oligosaccharides in binding studies to some of the enzymes participating in the biosynthesis of this polymer. The presence of aromatic rings, such as the 4-methoxyphenyl group at the anomeric position, should be avoided because these groups can significantly modify the binding mode between the CS and the enzymes. For this reason, we introduced an isopropyl group in β-position of the anomeric center of the glycosyl acceptor. We hypothesized that the isopropyl moiety would not affect the CS–enzyme interaction. Trichloroacetimidate **12** was obtained by oxidative removal of the 4-methoxyphenyl group with CAN at 0 ºC and further treatment with trichloroacetonitrile and catalytic amounts of DBU. The glycosylation reaction with 2-propanol using TMSOTf as Lewis acid at 0 ºC cleanly provided the β-isopropyl glycoside **13** in 65% yield. Interestingly, no α-anomer was detected in the reaction mixture. Finally, cleavage of the levulinyl group using hydrazine monohydrate in pyridine/acetic acid buffer afforded compound **2**.

**Scheme 4:**
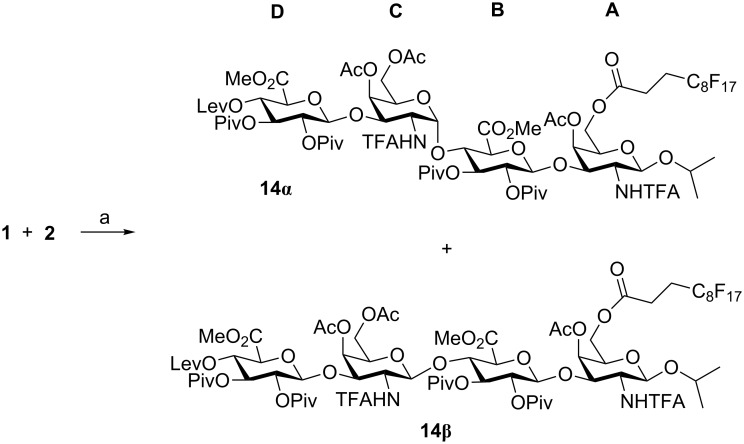
Reagents and conditions: a) TMSOTf, CH_2_Cl_2_, 0 °C, 30 min, 25% (**14α**) + 33% (**14β**).

With the required disaccharide building blocks in hand, the synthesis of tetrasaccharide **14β** was attempted using catalytic TMSOTf in CH_2_Cl_2_ at 0 ºC (Scheme 4). After F-SPE, two spots were detected in the TLC analysis of the fluorous containing fraction, in addition to unreacted acceptor **2**. Both compounds were separated by standard silica gel chromatography and unambiguously identified as α (**14α**) and β (**14β**) tetrasaccharides in a 1:1.3 ratio. The ^1^H NMR spectrum of **14α** showed diagnostic chemical shifts for protons H-1C (δ = 5.12 ppm in **14α**; δ = 4.94–4.78 ppm in **14β**) and H-2C (δ = 4.50 ppm in **14α**; δ = 3.52–3.43 ppm in **14β**). The value of the coupling constant *J*_1,2_ in ring C confirmed the configuration of the new glycosidic linkage (*J*_1,2_ = 3.2 Hz in **14α**; *J*_1,2_ = 8.2–8.5 Hz in **14β**). ^19^F NMR spectra also showed clear differences in the chemical shifts for the trifluoroacetyl signals (δ = −75.62 and −75.93 ppm in **14α**; δ = −75.85 and −75.89 ppm in **14β**).

The loss of stereocontrol in this glycosylation reaction was an unexpected result. The *N*-TFA group is considered as a participating group leading to the selective formation of 1,2-*trans* glycosidic linkages [37,45–46]. Moreover, in previous studies, we carried out the [2 + 2] coupling between several *N*-TFA-protected uronic acid-GalNAc disaccharides and the β-tetrasaccharides were exclusively isolated in good yields [7,36]. Importantly, the same reaction conditions were used for all these [2 + 2] condensations (20 mol % of TMSOTf with respect to the donor, 0 ºC, CH_2_Cl_2_ as solvent). As mentioned before, it has been reported that cyclic protecting groups like 4,6-*O*-benzylidene acetals in GalNAc trichloroacetimidates lead to the formation of α/β mixtures in glycosylations involving GlcA acceptors [43,47]. However, excellent β-stereoselectivity is observed when the benzylidene group is replaced by two acyl groups [43,47]. In the condensation of **1** and **2**, an α/β mixture was obtained despite the presence of two acetyls at positions 4 and 6 of the donor.

**Scheme 5:**
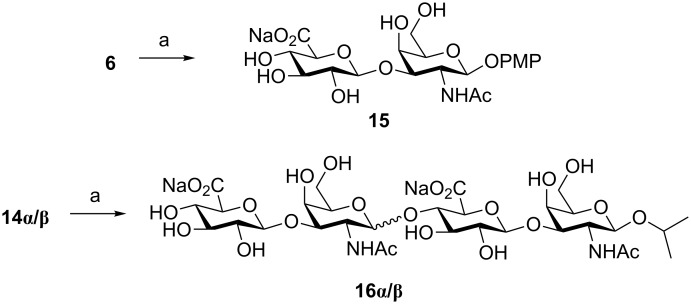
Reagents and conditions: a) LiOH, H_2_O_2_, THF, −5 °C to rt, 24 h, then NaOH, MeOH, 72 h, then Ac_2_O, Et_3_N, MeOH, 2 h, 38% (**15**); 30% (**16α**); 73% (**16β**).

In order to access to final CS sequences ready for biological assays, deprotection of dimer **6** and tetramer **14β** were accomplished (Scheme 5). Compound **14α** was also submitted to the deprotection sequence to obtain a non-natural α-containing oligosaccharide. Basic hydrolysis using H_2_O_2_/LiOH and then NaOH followed by selective *N*-acetylation in MeOH afforded CS deprotected derivatives in moderate to good yields. The structures of these compounds were confirmed by NMR and mass spectrometry. NMR data were in good agreement with those reported in the literature for similar CS oligosaccharides.

**Scheme 6:**
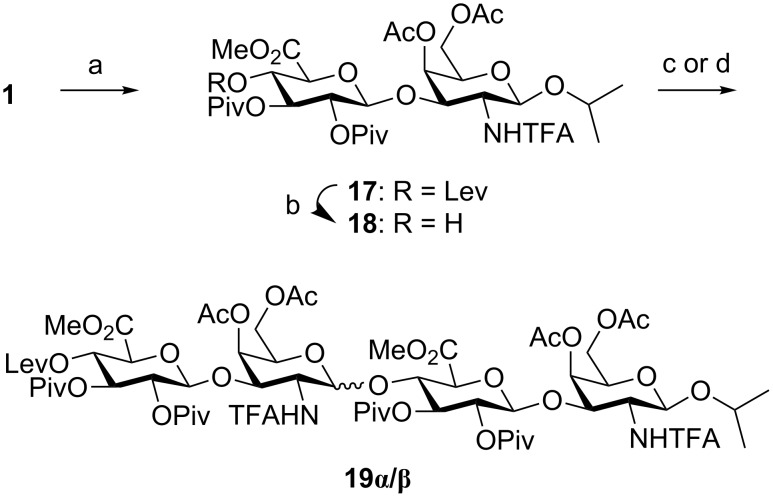
Reagents and conditions: a) 2-propanol, TMSOTf, CH_2_Cl_2_, 0 °C, 30 min, 73%; b) NH_2_NH_2_·H_2_O, Py/AcOH, CH_2_Cl_2_, 1 h, 89%; c) **1**, TMSOTf, CH_2_Cl_2_, 0 °C, 30 min, 18% (**19α**) + 53% (**19β**); d) **1**, TMSOTf, CH_3_CN, −20 °C, 30 min, 10% (**19α**) + 40% (**19β**).

The perfluorinated tag of acceptor **2** may strongly influence on the chemical properties of this derivative. To rule out any effect coming from the fluorous tag in the glycosylation stereochemical outcome, we decided to perform the coupling between **1** and the 4,6-di-*O*-acetyl disaccharide **18** (Scheme 6). Trichloroacetimidate **1** was glycosylated with 2-propanol and disaccharide **17** was isolated in good 73% yield. Noteworthy, exclusive formation of the β-product was observed. Nevertheless, the glycosylation of **1** and **18** again provided a mixture of the corresponding α and β-tetrasaccharides **19α** and **19β** in 71% overall yield (1:2.9 α/β ratio). The use of acetonitrile as solvent and lower temperature (−20 ºC), typical conditions for favoring the formation of the β-bond, increased the β-selectivity (1:4 α/β ratio) but also decreased the overall yield (50%).

**Scheme 7:**
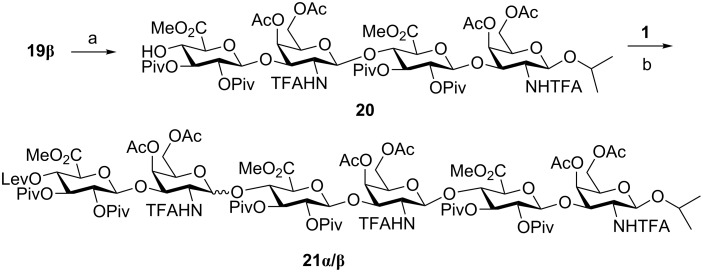
Reagents and conditions: a) NH_2_NH_2_·H_2_O, Py/AcOH, CH_2_Cl_2_, 1 h, 55%; b) TMSOTf, CH_2_Cl_2_, 0 °C, 30 min, 18% (**21α**) + 20% (**21β**).

Similarly, a loss of β-selectivity was observed at the hexasaccharide stage (Scheme 7). Tetrasaccharide acceptor **20** was prepared from **19β** by using hydrazine monohydrate. Glycosylation with **1** afforded α/β-hexasaccharides **21α** and **21β** that could be separated by silica gel chromatography and characterized by NMR and mass spectrometry. Overall, our results indicate an unexpected loss of β-selectivity in glycosylations involving 4,6-di-*O*-acylated *N*-TFA-protected GalNAc donors and GlcA acceptors that is not due to the presence of the fluorous tail. We hypothesized that the substitution pattern of the GlcA acceptor was responsible for the displayed stereochemistry and some experiments with model monosaccharides were planned to confirm this fact.

**Scheme 8:**
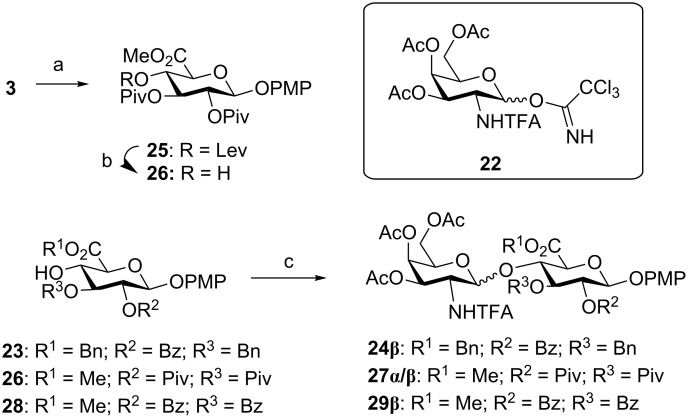
Reagents and conditions: a) 4-Methoxyphenol, TMSOTf, CH_2_Cl_2_, 0 °C, 50 min, 92%; b) NH_2_NH_2_·H_2_O, Py/AcOH, CH_2_Cl_2_, 1 h, 84%; c) **22**, TMSOTf, CH_2_Cl_2_, 0 °C, 85% for **24β;** 75% for **27α/β (**1:2.6 α/β ratio); 70% for **29β**.

In a previous work, the condensation between *N*-TFA-protected trichloroacetimidate **22** and 2-*O*-benzoyl-3-*O*-benzyl-GlcA derivative **23** was performed and β-disaccharide **24β** was exclusively isolated in excellent 85% yield (Scheme 8) [8]. In order to analyze the influence of the acceptor structure on the glycosylation outcome, we decided to prepare 2,3-di-*O*-pivaloyl compound **26** with the GlcA protecting group distribution used in this study. For this purpose, donor **3** was glycosylated with 4-methoxyphenol and then treated with hydrazine monohydrate in a pyridine/acetic acid/CH_2_Cl_2_ mixture to afford acceptor **26**. Using the same glycosylation conditions, the reaction between **22** and **26** gave a mixture of α (21%) and β (54%) disaccharides (**27α/β**). The coupling of **22** and the 2,3-di-*O*-benzoyl derivative **28** [48] was also tested. Interestingly, no α-product was detected in the reaction mixture and β-disaccharide **29β** was exclusively isolated in good 70% yield. Our results suggest that the loss of β-selectivity observed in [2 + 2] couplings can be attributed to the presence of bulky pivaloyl groups at positions 2 and 3 of the glucuronic acid acceptor.

## Conclusion

We planned a fluorous-tag-assisted approach for the synthesis of CS oligomers. A fully deprotected tetrasaccharide was successfully prepared for biological studies. This synthesis involved the [2 + 2] coupling of disaccharide building blocks. The use of a fluorous tag facilitated the isolation of the fluorinated species from the glycosylation reaction mixture and aided in the detection of a significant amount of the undesired α-anomer. This unexpected stereochemical outcome was also observed in [2 + 2] and [2 + 4] glycosylation reactions between building blocks lacking the fluorous tail.

For the GlcA moieties of the CS chains, we chose a 2,3-di-*O*-pivaloylated building block. Pivaloyl groups are widely employed in carbohydrate chemistry and are especially indicated for the protection of 2-OH groups of glycosyl donors because they minimize the formation of orthoester byproducts. However, glycosylation experiments between monosaccharide units revealed that the pivaloyl functions in the glycosyl acceptor favored the formation of the 1,2-*cis* glycosidic bond even in the presence of the *N*-TFA participating group in the glycosyl donor. In contrast, 2,3-di-*O*-benzoyl/benzyl GlcA derivatives exclusively gave the β-anomer. Overall, our results highlight the influence of the acceptor structure and reactivity on the stereoselectivity of glycosylation reactions [49] and give information on the design of building blocks for the synthesis of long CS sequences.

## Supporting Information

File 1 Experimental part and NMR spectra.
