# Supplementary material for: Unexpected loss of stereoselectivity in glycosylation reactions during the synthesis of chondroitin sulfate oligosaccharides
Source: Beilstein J Org Chem. 2019 Jan 15;15:137–44. doi: 10.3762/bjoc.15.14 (PMC6350880; doi:10.3762/bjoc.15.14)
Supplement: File 1 — Experimental part and NMR spectra. [file Beilstein_J_Org_Chem-15-137-s001.pdf]

BEILSTEIN JOURNAL OF ORGANIC CHEMISTRY

Supporting Information

for

Unexpected loss of stereoselectivity in glycosylation reactions during the synthesis of chondroitin sulfate oligosaccharides

Teresa Mena-Barragán, José L. de Paz and Pedro M. Nieto

Beilstein J. Org. Chem. **2019,** *15,* 137–144. doi:10.3762/bjoc.15.14

Experimental part and NMR spectra

License and Terms: This is a supporting information file under the terms of the Creative Commons Attribution License (http://creativecommons.org/licenses/by/4.0). Please note that the reuse, redistribution and reproduction in particular requires that the authors and source are credited.
The license is subject to the *Beilstein Journal of Organic Chemistry* terms and conditions: (https://www.beilstein-journals.org/bjoc)

Table of Contents

	**Page**
**Experimental Part**	S2–S19
**NMR spectra**	S20–S47

General methods

Optical rotations were measured with a Perkin Elmer 341 polarimeter, using a sodium lamp (λ = 589 nm) at 25 ºC in 1 dm tubes. NMR experiments were performed using Bruker Avance III-400 and DRX-500 spectrometers. 2-D COSY and HSQC experiments were carried out to assist in signal assignment. Unit A refers to the reducing end monosaccharide in the NMR data. For electrospray mass spectra (ESIMS), we employed a Bruker Esquire 6000 equipment. High-resolution mass spectra (HRMS) were performed at Centro de Investigación, Tecnología e Innovación, University of Seville (CITIUS). Thin-layer chromatography (TLC) analyses were performed on silica gel 60F-254 precoated plates from Merck. The compounds were detected by UV visualization (λ = 254 nm) and by staining with 5% v/v anisaldehyde/5% v/v H_2SO_4/0.2% v/v AcOH in EtOH or 0.2% w/v cerium (IV) sufate/5% w/v ammonium molybdate tetrahydrate in 1 M H_2SO_4 followed by heating at over 200 ºC. Column chromatography was performed on silica gel 60 from Merck (15–40 µm or 63–200 µm). For F-SPE purification, we followed our previously reported experimental procedure [1,2]. Briefly, FluoroFlash silica gel (5 g., from Sigma-Aldrich) was introduced in a glass chromatography column (1.7 cm diameter). The F-SPE column was washed with DMF (2 mL) and then preconditioned with MeOH/H_2O 4:1 (15 mL). Next, the crude sample (100–300 mg) was dissolved in DMF/H_2O 9:1 (0.8 mL) and loaded on the column. The fluorophobic elution was carried out with 15 mL of MeOH/H_2O 4:1. The fluorous compounds were then eluted using 100% acetone (20 mL).

4-Methoxyphenyl 3-*O*-(methyl 4-*O*-levulinyl-2,3-di-*O*-pivaloyl-β-D-glucopyranosyluronate)-4,6-*O*-di-*tert*-butylsilylene-2-deoxy-2-trifluoroacetamido-β-D-galactopyranoside (5). In a manner similar to a procedure from [3], glycosyl acceptor **4** (162 mg, 0.311 mmol) and D-glucuronic acid trichloroacetimidate **3** (270 mg, 0.436 mmol) were combined in a flask, coevaporated with toluene and dried under vacuum. The starting materials were dissolved in dry CH_2Cl_2 (4 mL) and further dried by stirring over freshly activated 4 Å molecular sieves for 15 min. TMSOTf (158 µL of a 0.28 M solution in CH_2Cl_2; 0.044 mmol) was added at 0 ºC. After 30 min at 0 ºC, the reaction was quenched with Et_3N (2 mL) and filtered, and the solvent was removed under reduced pressure. The residue was purified by silica gel column chromatography using 1:1 EtOAc-petroleum ether as eluent to give compound **5** as a colourless oil (294 mg, 97%). R_f 0.30 (1:1 EtOAc/petroleum ether). $[\alpha]_D$ +4º (*c* 1.0, CH_2Cl_2). ^{1}H NMR (400 MHz, $CDCl_3$) δ 7.41 (d, 1 H, $J_{2,NH}$ = 6.8 Hz, NH), 6.98 (d, 2 H, $^3J_{H,H}$ = 9.0 Hz, OPMP), 6.82 (d, 2 H, $^3J_{H,H}$ = 9.0 Hz, OPMP), 5.52 (d, 2 H, $J_{1,2}$ = 8.2 Hz, H-1A, H-1B), 5.25-5.22 (m, 2 H, H-3B, H-4B), 5.08-5.03 (m, 1 H, H-2B), 4.72 (d, 1 H, $J_{3,4}$ = 2.6 Hz, H-4A), 4.54 (dd, 1 H, $J_{2,3}$ = 11.6 Hz, H-3A), 4.27 (dd, 1 H, $J_{6a,6b}$ = 12.3 Hz, $J_{5,6a}$ = 2.0 Hz, H-6aA), 4.22 (dd, 1 H, $J_{5,6b}$ = 1.5 Hz, H-6bA), 4.07-4.00 (m, 2 H, H-2A, H-5B), 3.80, 3.78 (s, 6 H, COOMe, OCH_3), 3.49 (m, 1 H, H-5A), 2.74-2.50 (m, 4 H, CH_2 (Lev)), 2.20 (s, 3 H, CH_3 (Lev)), 1.19,

1.12 (s, 36 H, CH_3 (Piv), $Si(CH_3)_2$). ^{13}C NMR (100.6 MHz, $CDCl_3$) δ 205.7 (CO, Lev), 177.2, 176.3, 171.2, 167.4 (CO, Lev, Piv, COOMe), 157.8 (q, *C*OCF_3), 156.0, 151.0 (Ar-C), 120.3, 114.5 (Ar-CH), 115.5 (q, CO*C*F_3), 99.3, 98.8 (C-1A, C-1B), 74.2, 74.1 (C-3A, C-4A), 71.7, 71.6, 71.4 (C-3B, C-5B, C-5A), 70.8 (C-2B), 69.2 (C-4B), 67.0 (C-6A), 55.6 (COO*C*H_3 or OCH_3), 54.7 (C-2A), 53.2 (COO*C*H_3 or OCH_3), 38.8, 38.7 (*C*$(CH_3)_3$, Piv), 37.4 (*C*H_2COO, Lev), 29.8 (CH_3, Lev), 27.6, 27.1, 27.0 (*C*H_2CH_2COO (Lev), CH_3 (Piv), $Si(CH_3)_2$). HR MS: *m/z*: calcd. for $C_{45}H_{66}O_{17}NF_3NaSi$: 1000.3944; found: 1000.3934 $[M + Na]^+$.

4-Methoxyphenyl 3-*O*-(methyl 4-*O*-levulinyl-2,3-di-*O*-pivaloyl-β-D-glucopyranosyluronate)-4,6-di-*O*-acetyl-2-deoxy-2-trifluoroacetamido-β-D-galactopyranoside (7). In a manner similar to a procedure from [3], an excess of $(HF)_n$·Py (1.09 mL, 60 mmol) was added at 0 ºC under an argon atmosphere to a solution of **5** (294 mg, 0.30 mmol) in dry THF (13 mL). After 20 h at 0 ºC, the mixture was diluted with CH_2Cl_2 and washed with cool water and saturated aqueous $NaHCO_3$ solution until neutral pH. The organic layers were dried ($MgSO_4$), filtered and concentrated in vacuo to give **6** as a colourless amorphous solid (227 mg, 90%). R_f 0.39 (9:1 CH_2Cl_2/MeOH). ^{1}H NMR (400 MHz, 5:1 $CDCl_3$-MeOD) δ 6.88 (d, 2 H, $^3J_{H,H}$ = 9.0 Hz, OPMP), 6.74 (d, 2 H, $^3J_{H,H}$ = 9.0 Hz, OPMP), 5.23 (t, 1 H, $J_{2,3} = J_{3,4}$ = 9.2 Hz, H-3B), 5.14 (t, 1 H, $J_{4,5}$ = 9.2 Hz, H-4B), 4.99 (dd, 1 H, $J_{1,2}$ = 7.8 Hz, H-2B), 4.93 (d, 1 H, $J_{1,2}$ = 8.4 Hz, H-1A), 4.80 (d, 1 H, H-1B), 4.24 (dd, 1 H, $J_{2,3}$ = 10.8 Hz, $J_{3,4}$ = 3.0 Hz, H-3A), 4.08 (m, 2 H, H-2A, H-5B), 3.73 (m, 3 H, H-4A, H-6aA, H-6bA), 3.71, 3.70 (s, 6 H, COOMe, OCH_3), 3.57 (m, 1 H, H-5A), 2.64-2.38 (m, 4 H, CH_2 (Lev)), 2.13 (s, 3 H, CH_3 (Lev)), 1.09, 1.07 (s, 18 H, CH_3 (Piv)).

Acetic anhydride (0.55 mL) was added at 0 ºC to a solution of compound **6** (110 mg, 0.131 mmol) in dry pyridine (0.55 mL). After 24 h at room temperature, the mixture was diluted with CH_2Cl_2 and washed with 1 M HCl, saturated aqueous $NaHCO_3$ solution and water. The organic layers were dried ($MgSO_4$), filtered and concentrated in vacuo and the resulting residue was purified by column chromatography using 2:1 EtOAc-petroleum ether as eluent to give compound **7** as a colourless oil (117 mg, 97%). R_f 0.51 (2:1 EtOAc/acetone). $[\alpha]_D$ -2º (*c* 0.61, CH_2Cl_2). ^{1}H NMR (400 MHz, $CDCl_3$) δ 6.94 (d, 2 H, $^3J_{H,H}$ = 8.9 Hz, OPMP), 6.87 (d, 1 H, $J_{2,NH}$ = 7.3 Hz, NH), 6.81 (d, 2 H, $^3J_{H,H}$ = 8.9 Hz, OPMP), 5.51 (d, 1 H, $J_{3,4}$ = 3.0 Hz, H-4A), 5.26 (m, 2 H, H-3B, H-4B), 5.20 (d, 1 H, $J_{1,2}$ = 8.3 Hz, H-1A), 5.02 (t, 1 H, H-2B), 4.77 (d, 1 H, $J_{1,2}$ = 7.5 Hz, H-1B), 4.67 (dd, 1 H, $J_{2,3}$ = 11.1 Hz, H-3A), 4.17-4.12 (m, 2 H, H-6aA, H-6bA), 4.07-4.05 (m, 1 H, H-5B), 4.02-3.97 (m, 2 H, H-2A, H-5A), 3.78 (s, 6 H, COOMe, OCH_3), 2.75-2.44 (m, 4 H, CH_2 (Lev)), 2.18 (s, 3 H, CH_3 (Lev)), 2.16, 2.09 (s, 6 H, CH_3 (Ac)), 1.16, 1.13 (s, 18 H, CH_3 (Piv)). ^{13}C NMR (100.6 MHz, $CDCl_3$) δ 205.8 (CO, Lev), 177.1, 176.3, 171.0, 170.5, 169.8, 167.0 (CO, Lev, Piv, COOMe, Ac), 157.8 (q, $^2J_{C,F}$ = 37.0 Hz, *C*OCF_3), 155.9, 150.9 (Ar-C), 118.8, 114.6 (Ar-CH), 115.6 (q, $^1J_{C,F}$ = 298.5 Hz, CO*C*F_3), 99.3 (C-1A, C-1B), 72.3 (C-5B), 71.9 (C-3A), 71.5 (C-5A), 71.4 (C-3B or C-4B), 70.5 (C-2B), 69.3 (C-3B or C-4B), 68.5 (C-

4A), 62.0 (C-6A), 55.6 (COO*C*H_3 or OCH_3), 54.5 (C-2A), 53.1 (COO*C*H_3 or OCH_3), 38.8 (*C*(CH_3)$_3$,Piv), 37.4 (*C*H_2COO, Lev), 29.8 (CH_3, Lev), 27.5 (*C*$H_2$$CH_2$COO, Lev), 27.0 ($CH_3$, Piv), 20.7, 20.6 ($CH_3$, Ac). ^{19}F NMR (376 MHz, $CDCl_3$): δ -75.67 (s, 3F). HR MS: *m/z*: calcd. for $C_{41}H_{54}O_{19}NF_3Na$: 944.3134; found: 944.3123 $[M + Na]^+$.

3-*O*-(Methyl 4-*O*-levulinyl-2,3-di-*O*-pivaloyl-β-D-glucopyranosyluronate)-4,6-di-*O*-acetyl-2-deoxy-2-trifluoroacetamido-α-D-galactopyranose (8). CAN (260 mg, 0.476 mmol) was added to a solution of **7** (110 mg, 0.119 mmol) in CH_3CN/CH_2Cl_2/water (6:3:1, 6.7 mL) and the mixture was stirred for 1 h at 0 ºC. Then, the mixture was diluted with EtOAc, washed with water and saturated aqueous $NaHCO_3$ solution, dried ($MgSO_4$), filtered and concentrated in vacuo. The resulting residue was purified by column chromatography using 3:1 EtOAc-petroleum ether as eluent to give compound **8** as a pale yellow amorphous solid (96 mg, 99%). R_f 0.36 (3:1 EtOAc/petroleum ether). ^{1}H NMR (400 MHz, $CDCl_3$) δ 7.07 (d, 1 H, $J_{2,NH}$ = 8.2 Hz, NH), 5.46 (d, 1 H, $J_{3,4}$ = 2.2 Hz, H-4A), 5.43 (t, 1 H, $J_{1,2}$ = $J_{1,OH}$ = 3.2 Hz, H-1A), 5.30 (m, 1 H, H-3B), 5.21 (m, 1 H, H-4B), 5.07 (d, 1 H, H-2B), 4.82 (d, 1 H, $J_{1,2}$ = 8.0 Hz, H-1B), 4.49 (m, 1 H, H-2A), 4.41-4.35 (m, 2 H, H-3A, H-5A), 4.17 (dd, 1 H, $J_{6a,6b}$ = 11.5 Hz, $J_{5,6a}$ = 6.1 Hz, H-6aA), 4.09 (d, 1 H, $J_{4,5}$ = 9.8 Hz, H-5B), 4.02 (dd, 1 H, $J_{5,6b}$ = 7.3 Hz, H-6bA), 3.78 (s, 4 H, COOMe, OH), 2.72-2.45 (m, 4 H, CH_2 (Lev)), 2.19 (s, 3 H, CH_3 (Lev)), 2.13, 2.10 (s, 6 H, CH_3 (Ac)), 1.15, 1.13 (s, 18 H, CH_3 (Piv)). ^{13}C NMR (100.6 MHz, $CDCl_3$) δ 205.9 (CO, Lev), 177.2, 176.8, 171.1, 170.8, 170.0 (CO, Lev, Piv, Ac), 167.0 (COOMe), 157.4 (q, *C*OCF_3), 115.7 (q, CO*C*F_3), 98.5 (C-1B), 91.3 (C-1A), 72.4 (C-5B), 71.4 (C-3B), 70.2 (C-2B), 69.8 (C-3A), 69.5 (C-4B), 68.6 (C-4A), 67.3 (C-5A), 62.4 (C-6A), 53.1 (COO*C*H_3), 50.2 (C-2A), 38.8, 38.7 (*C*(CH_3)$_3$,Piv), 37.5 (*C*H_2COO, Lev), 29.8 (CH_3, Lev), 27.5 (*C*$H_2$$CH_2$COO, Lev), 26.9 ($CH_3$, Piv), 20.8, 20.6 ($CH_3$, Ac). ^{19}F NMR (376 MHz, $CDCl_3$): δ -75.63 (s, 3F). HR MS: *m/z*: calcd. for $C_{34}H_{48}O_{18}NF_3Na$: 838.2716; found: 838.2705 $[M + Na]^+$.

***O*-[3-*O*-(Methyl 4-*O*-levulinyl-2,3-di-*O*-pivaloyl-β-D-glucopyranosyluronate)-4,6-di-*O*-acetyl-2-deoxy-2-trifluoroacetamido-α,β-D-galactopyranosyl] trichloroacetimidate (1)**. In a manner similar to a procedure from [3], trichloroacetonitrile (147 µL, 1.47 mmol) and catalitic DBU (60 µL of a 0.084 M solution in dry CH_2Cl_2) were added to a solution of **8** (80 mg, 0.098 mmol) in dry CH_2Cl_2 (3 mL). After stirring for 9 h at room temperature, the reaction mixture was concentrated to dryness. The residue was purified by column chromatography using 1:1 EtOAc-toluene + 1% Et_3N as eluent to give **1** as a mixture of α-β anomers (92 mg, 98%). R_f 0.64 (2:1 EtOAc/petroleum ether). ^{1}H NMR for α anomer (400 MHz, $CDCl_3$) δ 8.81 (s, 1 H, NH acetimidate), 6.96 (d, 1 H, $J_{2,NH}$ = 7.3 Hz, NH amide), 6.60 (d, 1 H, $J_{1,2}$ = 3.6 Hz, H-1A), 5.53 (d, 1 H, $J_{3,4}$ = 2.2 Hz, H-4A), 5.35 (m, 1 H, H-3B), 5.13 (m, 1 H, H-4B), 5.11 (m, 1 H, H-2B), 4.87 (d, 1 H, $J_{1,2}$ = 7.8 Hz, H-1B), 4.71 (ddd, 1 H, $J_{2,3}$ = 10.9 Hz, H-2A), 4.38 (dd, 1 H, H-3A), 4.35 (m, 1 H, H-5A), 4.21 (dd, 1 H, $J_{6a,6b}$ = 11.4 Hz, $J_{5,6a}$ = 5.9 Hz, H-6aA), 4.11 (d, 1 H, $J_{4,5}$ = 9.8 Hz, H-5B), 4.00 (dd, 1 H, $J_{5,6b}$ = 6.8 Hz, H-6bA), 3.76 (s, 3 H, COOMe), 2.74-2.46 (m,

4 H, CH_2 (Lev)), 2.20 (s, 3 H, CH_3 (Lev)), 2.15, 2.06 (s, 6 H, CH_3 (Ac)), 1.18, 1.16 (s, 18H, CH_3 (Piv)). ^{13}C NMR (100.6 MHz, $CDCl_3$) δ 205.6 (CO, Lev), 177.1, 176.8, 171.1, 170.4, 169.7, 166.6 (CO Lev, Piv, COOMe, Ac), 160.4 (C=NH), 157.7 (q, *C*OCF_3), 115.0 (q, CO*C*F_3), 98.1 (C-1B), 94.4 (C-1A), 90.7 (CCl_3), 72.4 (C-5B), 71.2 (C-3B), 70.4, 70.0, 69.9, 69.3 (C-3A, C-5A, C-2B, C-4B), 67.4 (C-4A), 61.7 (C-6A), 53.1 (CO_2Me), 49.7 (C-2A), 38.9, 38.8 (*C*$(CH_3)_3$, Piv), 37.3 (*C*H_2COO, Lev), 29.8 (CH_3, Lev), 27.5 (*C*H_2CH_2COO, Lev), 27.1 (CH_3, Piv), 20.6 (CH_3, Ac). ESI MS: *m/z*: calcd. for $C_{36}H_{48}O_{18}N_2Cl_3F_3Na$: 981.2; found: 981.2 $[M + Na]^+$.

4-Methoxyphenyl 3-*O*-(methyl 4-*O*-levulinyl-2,3-di-*O*-pivaloyl-β-D-glucopyranosyluronate)-2-deoxy-6-*O*-4,4,5,5,6,6,7,7,8,8,9,9,10,10,11,11,11-heptadecafluoroundecanoyl-2-trifluoroacetamido-β-D-galactopyranoside (9). Et_3N (100 µL, 0.716 mmol), DMAP (11 mg, 0.09 mmol) and 4,4,5,5,6,6,7,7,8,8,9,9,10,10,11,11,11-heptadecafluoroundecanoyl chloride (200 mg, 0.394 mmol) were added to a solution of **6** (300 mg, 0.358 mmol) in dry 5:1 DMF/CH_2Cl_2 (15 mL) at 0 ºC. After stirring for 6 h at room temperature, the mixture was diluted with EtOAc, washed with saturated aqueous $NaHCO_3$ solution, dried ($MgSO_4$), filtered and concentrated in vacuo. The resulting residue was purified by column chromatography using 1:1 EtOAc-petroleum ether as eluent to give compound **9** as a colourless oil (329 mg, 70%). R_f 0.65 (1:1 EtOAc/petroleum ether). $[\alpha]_D$ -8º (*c* 0.88, 1:1 CH_2Cl_2-MeOH). ^{1}H NMR (400 MHz, $CDCl_3/CD_3OD$ 1:1) δ 6.92 (d, 2 H, $^3J_{H,H}$ = 9.4 Hz, OPMP), 6.78 (d, 2 H, $^3J_{H,H}$ = 9.40 Hz, OPMP), 5.31 (t, 1 H, $J_{2,3}$ = $J_{3,4}$ = 9.3 Hz, H-3B), 5.18 (t, 1 H, $J_{4,5}$ = 9.3 Hz, H-4B), 5.07 (dd, 1 H, $J_{1,2}$ = 7.8 Hz, H-2B), 4.90 (m, 2 H, H-1A, H-1B), 4.47 (dd, 1 H, $J_{6a,6b}$ = 11.4 Hz, $J_{5,6a}$ = 7.8 Hz, H-6aA), 4.35 (dd, 1 H, $J_{5,6b}$ = 5.1 Hz, H-6bA), 4.23-4.18 (m, 3 H, H-2A, H-3A, H-5B), 4.13 (m, 1 H, H-4A), 3.86 (m, 1 H, H-5A), 3.76, 3.74 (s, 6 H, COOMe, OMe), 2.74-2.44 (m, 8 H, CH_2 (Lev), $CH_2CH_2C_8F_{17}$), 2.19 (s, 3 H, CH_3 (Lev)), 1.14, 1.13 (s, 18H, CH_3 (Piv)). ^{13}C NMR (100.6 MHz, $CDCl_3/CD_3OD$ 1:1) δ 207.0 (CO, Lev), 177.5, 171.5, 171.1, 167.9 (CO, Lev, Piv, COOMe, *C*O$CH_2CH_2C_8F_{17}$), 157.0 (q, *C*OCF_3), 155.6, 151.4 (Ar-C), 118.4, 114.3 (Ar-CH), 115.6 (q, CO*C*F_3), 100.4, 1001 (C-1A, C-1B), 76.1 (C-3A), 72.2 (C-5A), 71.7 (C-3B, C-5B), 70.8 (C-2B), 69.6 (C-4B), 67.7 (C-4A), 63.7 (C-6A), 55.2 (CO_2Me or OPMP), 52.8 (C-2A), 52.4 (CO_2Me or OPMP), 38.7 (*C*$(CH_3)_3$,Piv), 37.1 (*C*H_2COO, Lev), 29.3 (CH_3, Lev), 27.4 (*C*H_2CH_2COO, Lev), 26.6 (CH_3, Piv), 26.2 (*C*$H_2CH_2C_8F_{17}$), 25.2 (t, *C*$H_2C_8F_{17}$). HR MS: *m/z*: calcd. for $C_{48}H_{53}O_{18}NF_{20}Na$: 1334.2835; found: 1334.2821 $[M + Na]^+$.

3-*O*-(Methyl 4-*O*-levulinyl-2,3-di-*O*-pivaloyl-β-D-glucopyranosyluronate)-4-*O*-acetyl-2-deoxy-6-*O*-4,4,5,5,6,6,7,7,8,8,9,9,10,10,11,11,11-heptadecafluoroundecanoyl-2-trifluoroacetamido-α,β-D-galactopyranose (11). Acetic anhydride (1.75 mL) was added at 0 ºC to a solution of compound **9** (290 mg, 0.221 mmol) in dry pyridine (1.75 mL). After 24 h at room temperature, the mixture was diluted with CH_2Cl_2 and washed with 1 M HCl, saturated

aqueous $NaHCO_3$ solution and water. The organic layers were dried ($MgSO_4$), filtered and concentrated in vacuo. The resulting residue was purified by using a fluorous solid-phase extraction cartridge packed with silica gel containing a perfluorooctylethylsilyl bonded phase. Fluorophobic wash was performed with 4:1 MeOH/H_2O. Fluorophilic elution with acetone afforded compound **10** as a colourless oil (290 mg, 94%). R_f 0.55 (2:1 EtOAc/petroleum ether). ^{1}H NMR (400 MHz, $CDCl_3$) δ 6.94 (d, 2 H, $^3J_{H,H}$ = 9.0 Hz, OPMP), 6.78 (d, 2 H, OPMP), 6.79 (s, 1 H, NH), 5.52 (d, 1 H, $J_{3,4}$ = 3.2 Hz, H-4A), 5.26 (m, 3 H, H-1A, H-3B, H-4B), 5.03 (dd, 1 H, $J_{2,3}$ = 9.0 Hz, $J_{1,2}$ = 7.9 Hz, H-2B), 4.78 (d, 1 H, H-1B), 4.70 (dd, 1 H, $J_{2,3}$ = 11.1 Hz, H-3A), 4.23 (m, 2 H, H-6aA, H-6bA), 4.03 (m, 3 H, H-2A, H-5A, H-5B), 3.78 (s, 6 H, COOMe, OMe), 2.73-2.45 (m, 8 H, CH_2 (Lev), $CH_2CH_2C_8F_{17}$), 2.19 (s, 3 H, CH_3 (Lev)), 2.17 (s, 3 H, CH_3 (Ac)), 1.17, 1.14 (s, 18H, CH_3 (Piv)). ESI MS: *m/z*: calcd. for $C_{50}H_{55}O_{19}NF_{20}Na$: 1376.3; found: 1375.9 [M + Na]$^+$.

CAN (131 mg, 0.240 mmol) was added to a solution of **10** (81 mg, 0.060 mmol) in $CH_3CN/CH_2Cl_2/H_2O$ (6:3:0.5, 4.75 mL) and the mixture was stirred for 2 h at 0 ºC. Then, the mixture was diluted with EtOAc, washed with water and saturated aqueous $NaHCO_3$ solution, dried ($MgSO_4$), filtered and concentrated in vacuo. The resulting residue was purified by column chromatography using 1:2 → 1:1 EtOAc-toluene as eluent to give compound **11** as a mixture of α-β anomers (40 mg, 54%). R_f 0.61 (2:3 EtOAc/toluene). ^{1}H NMR for α anomer (400 MHz, $CDCl_3$) δ 7.10 (d, 1 H, $J_{2,NH}$ = 8.3 Hz, NH amide), 5.45 (m, 2 H, H-1A, H-4A), 5.31 (t, 1 H, $J_{2,3}$ = $J_{3,4}$ = 9.1 Hz, H-3B), 5.21 (t, 1 H, $J_{4,5}$ = 9.1 Hz, H-4B), 5.09 (dd, 1 H, $J_{1,2}$ = 7.8 Hz, H-2B), 4.83 (d, 1 H, H-1B), 4.51 (ddd, 1 H, $J_{2,3}$ = 11.0 Hz, $J_{1,2}$ = 2.9 Hz, H-2A), 4.45-4.40 (m, 2 H, H-3A, H-5A), 4.20 (dd, 1 H, $J_{6a,6b}$ = 11.4 Hz, $J_{5,6a}$ = 6.7 Hz, H-6aA), 4.11 (d, 1 H, H-5B), 4.08 (dd, 1 H, $J_{5,6b}$ = 6.5 Hz, H-6bA), 3.78 (s, 3 H, COOMe), 2.73-2.49 (m, 8 H, CH_2 (Lev), $CH_2CH_2C_8F_{17}$), 2.20 (s, 3 H, CH_3 (Lev)), 2.13 (s, 3 H, CH_3 (Ac)), 1.16, 1.14 (s, 18H, CH_3, (Piv)). ^{13}C NMR (100.6 MHz, $CDCl_3$) δ 205.8 (CO, Lev), 177.1, 176.7, 171.1, 170.8, 169.9, 167.1 (CO, Lev, Piv, COOMe, Ac, *C*O$CH_2CH_2C_8F_{17}$), 157.4 (q, *C*OCF_3), 115.6 (q, CO*C*F_3), 98.3 (C-1B), 91.4 (C-1A), 72.3 (C-5B), 71.3 (C-3B), 69.7 (C-3A or C-5A), 69.4, 69.3 (C-2B, C-4B), 68.5 (C-4A), 67.3 (C-3A or C-5A), 62.4 (C-6A), 53.1 (CO_2Me), 50.1 (C-2A), 38.8, 38.7 (*C*$(CH_3)_3$, Piv), 37.4 (*C*H_2COO, Lev), 29.7 (CH_3, Lev), 27.5 (*C*H_2CH_2COO, Lev), 26.9 (CH_3, Piv), 26.4 (*C*$H_2CH_2C_8F_{17}$), 25.3 (t, *C*$H_2C_8F_{17}$), 20.6 (CH_3, Ac). ^{19}F NMR (376 MHz, $CDCl_3$): *δ* -75.65 (s, 3F), -80.76 (t, 3F), -114.66 (m, 2F), -121.91 (m, 6F), -122.71 (m, 2F), -123.39 (m, 2F), -126.11 (m, 2F). HR MS: *m/z*: calcd. for $C_{43}H_{49}O_{18}NF_{20}Na$: 1270.2522; found: 1270.2508 [M + Na]$^+$.

***O*-[3-*O*-(Methyl 4-*O*-levulinyl-2,3-di-*O*-pivaloyl-β-D-glucopyranosyluronate)-4-*O*-acetyl-2-deoxy-6-*O*-4,4,5,5,6,6,7,7,8,8,9,9,10,10,11,11,11-heptadecafluoroundecanoyl-2-trifluoroacetamido-α,β-D-galactopyranosyl] trichloroacetimidate (12)**. In a manner similar to a procedure from [3], trichloroacetonitrile (100 µL, 0.480 mmol) and catalitic DBU (30 µL of

a 0.084 M solution in dry CH_2Cl_2) were added to a solution of **11** (40 mg, 0.032 mmol) in dry CH_2Cl_2 (2 mL). After stirring for 6 h at room temperature, the reaction mixture was concentrated to dryness. The residue was purified by column chromatography using 2:3 EtOAc/toluene + 1% Et_3N as eluent to give **12** as a mixture of α-β anomers (34 mg, 76%). R_f 0.69 (1:1 EtOAc/toluene). ^{1}H NMR for α anomer (400 MHz, $CDCl_3$) δ 8.80 (s, 1 H, NH acetimidate), 7.05 (d, 1 H, $J_{2,NH}$ = 7.2 Hz, NH amide), 6.59 (d, 1 H, $J_{1,2}$ = 3.4 Hz, H-1A), 5.50 (d, 1 H, $J_{3,4}$ = 2.8 Hz, H-4A), 5.35 (t, 1 H, $J_{2,3}$ = $J_{3,4}$ = 9.4 Hz, H-3B), 5.11 (m, 2 H, H-2B, H-4B), 4.88 (d, 1 H, $J_{1,2}$ = 7.8 Hz, H-1B), 4.71 (ddd, 1 H, $J_{2,3}$ = 10.9 Hz, H-2A), 4.41 (dd, 1 H, H-3A), 4.38 (m, 1 H, H-5A), 4.21 (dd, 1 H, $J_{6a,6b}$ = 11.6 Hz, $J_{5,6a}$ = 6.8 Hz, H-6aA), 4.11 (d, 1 H, $J_{4,5}$ = 10.0 Hz, H-5B), 4.08 (dd, 1 H, $J_{5,6b}$ = 7.2 Hz, H-6bA), 3.75 (s, 3 H, COOMe), 2.72-2.45 (m, 8 H, CH_2 (Lev), $CH_2CH_2C_8F_{17}$), 2.20 (s, 3 H, CH_3 (Lev)), 2.15 (s, 3 H, CH_3 (Ac)), 1.17, 1.15 (s, 18H, CH_3 (Piv)). ^{13}C NMR (100.6 MHz, $CDCl_3$) δ 205.5 (CO, Lev), 177.0, 176.8, 171.1, 170.5, 169.7, 166.6 (CO, Lev, Piv, COOMe, Ac, *C*O$CH_2CH_2C_8F_{17}$), 160.4 (C=NH), 157.8 (q, *C*OCF_3), 114.2 (q, CO*C*F_3), 98.1 (C-1B), 94.4 (C-1A,), 90.6 (CCl_3), 72.4 (C-5B), 71.2 (C-3B), 70.0, 69.8, 69.6, 69.3 (C-3A, C-5A, C-2B, C-4B), 67.5 (C-4A), 62.0 (C-6A), 53.1 (CO_2Me), 49.6 (C-2A), 38.9, 38.8 (*C*$(CH_3)_3$, Piv), 37.3 (*C*H_2COO, Lev), 29.7 (CH_3, Lev), 27.5 (*C*H_2CH_2COO, Lev), 26.9, 26.8 (CH_3, Piv), 26.3 (*C*$H_2CH_2C_8F_{17}$), 25.3 (t, *C*$H_2C_8F_{17}$), 20.6 (CH_3, Ac). ^{19}F NMR (376 MHz, $CDCl_3$): *δ* -75.56 (s, 3F), -80.74 (t, 3F), -114.68 (m, 2F), -121.91 (m, 6F), -122.69 (m, 2F), -123.40 (m, 2F), -126.09 (m, 2F). ESI MS: *m/z*: calcd. for $C_{45}H_{49}O_{18}N_2F_{20}Cl_3Na$: 1413.2; found: 1413.0 $[M + Na]^+$.

2-Propyl 3-*O*-(methyl 4-*O*-levulinyl-2,3-di-*O*-pivaloyl-β-D-glucopyranosyluronate)-4-*O*-acetyl-2-deoxy-6-*O*-4,4,5,5,6,6,7,7,8,8,9,9,10,10,11,11,11-heptadecafluoroundecanoyl-2-trifluoroacetamido-β-D-galactopyranoside (13). **12** (72 mg, 0.055 mmol) was coevaporated with toluene, dried under vacuum, dissolved in dry CH_2Cl_2 (1.5 mL) and further dried by stirring over freshly activated 4 Å molecular sieves for 15 min. 2-propanol (210 µL of a 0.52 M solution in CH_2Cl_2, 0.11 mmol) was added. Then, TMSOTf (100 µL of a 0.11 M solution in CH_2Cl_2, 0.011 mmol) was added at 0 ºC. After 30 min at 0 ºC, the reaction was quenched with Et_3N (1 mL) and filtered, and the solvent was removed under reduced pressure. The residue was purified by column chromatography using 1:3 EtOAc/toluene as eluent to give compound **13** as a colourless oil (46 mg, 65%). R_f 0.43 (2:5 EtOAc-toluene). $[\alpha]_D$ -3º (*c* 1.0, CH_2Cl_2). ^{1}H NMR (400 MHz, $CDCl_3$) δ 6.64 (d, 1 H, $J_{2,NH}$ = 7.3 Hz, NH), 5.48 (d, 1 H, $J_{3,4}$ = 3.0 Hz, H-4A), 5.25 (m, 2 H, H-3B, H-4B), 4.99 (m, 1 H, H-2B), 4.92 (d, 1 H, $J_{1,2}$ = 8.6 Hz, H-1A), 4.73 (m, 1 H, H-3A), 4.72 (d, 1 H, $J_{1,2}$ = 7.8 Hz, H-1B), 4.22 (dd, 1 H, $J_{6a,6b}$ = 11.4 Hz, $J_{5,6a}$ = 6.0 Hz, H-6aA), 4.15 (dd, 1 H, $J_{5,6b}$ = 6.9 Hz, H-6bA), 4.04 (m, 1 H, H-5B), 3.94 (m, 2 H, H-5A, CH (isopropyl)), 3.78 (s, 3 H, COOMe), 3.60 (m, 1 H, H-2A), 2.72-2.46 (m, 8 H, CH_2 (Lev), $CH_2CH_2C_8F_{17}$), 2.19 (s, 3 H, CH_3 (Lev)), 2.13 (s, 3 H, CH_3 (Ac)), 1.24 (d, 3 H, $^3J_{H,H}$ = 6.2 Hz, CH_3 (isopropyl)), 1.14, 1.13 (s, 18 H, CH_3 (Piv)), 1.13 (m, 3 H, CH_3 (isopropyl)). ^{13}C NMR

(100.6 MHz, $CDCl_3$) δ 205.7 (CO, Lev), 177.1, 176.3, 171.0, 170.5, 169.8, 167.0 (CO, Lev, Piv, COOMe, Ac, $COCH_2CH_2C_8F_{17}$), 157.7 (q, $COCF_3$), 115.4 (q, $COCF_3$), 99.4 (C-1B), 97.7 (C-1A), 73.0 (C-5A or CH isopropyl), 72.2 (C-5B), 71.8 (C-3A), 71.4 (C-3B or C-4B), 71.0 (C-5A or CH isopropyl), 70.6 (C-2B), 69.3 (C-3B or C-4B), 68.8 (C-4A), 62.5 (C-6A), 55.4 (C-2A), 52.9 ($COOCH_3$), 38.8, 38.7 ($C(CH_3)_3$, Piv), 37.4 (CH_2COO, Lev), 29.8 (CH_3, Lev), 27.5 (CH_2CH_2COO, Lev), 26.9 (CH_3, Piv), 26.4 ($CH_2CH_2C_8F_{17}$), 25.3 (t, $CH_2C_8F_{17}$), 23.2, 21.6 (CH_3, isopropyl), 20.6 (CH_3, Ac). HR MS: *m/z*: calcd. for $C_{46}H_{55}O_{18}NF_{20}Na$: 1312.2992; found: 1312.2980 $[M + Na]^+$.

2-Propyl 3-*O*-(methyl 2,3-di-*O*-pivaloyl-β-D-glucopyranosyluronate)-4-*O*-acetyl-2-deoxy-6-*O*-4,4,5,5,6,6,7,7,8,8,9,9,10,10,11,11,11-heptadecafluoroundecanoyl-2-trifluoroacetamido-β-D-galactopyranoside (2). In a manner similar to a procedure from [3], **13** (46 mg, 0.036 mmol) was dissolved in CH_2Cl_2 (1 mL) and hydrazine monohydrate (0.36 mL of a 0.25 M solution in 3:2 Py/AcOH) was added. After stirring at room temperature for 1 h, the reaction mixture was quenched with acetone (1 mL), diluted with CH_2Cl_2, washed with 1 N HCl, aqueous saturated $NaHCO_3$ and H_2O, dried over $MgSO_4$, filtered and concentrated *in vacuo*. The residue was purified by column chromatography using 1:2 EtOAc/petroleum ether as eluent to give **2** as an amorphous white solid (40 mg, 94%). R_f 0.59 (1:1 EtOAc-petroleum ether). $[\alpha]_D$ -4° (*c* 1.0, CH_2Cl_2). ^{1}H NMR (400 MHz, $CDCl_3$) δ 6.63 (d, 1 H, $J_{2,NH}$ = 7.0 Hz, NH), 5.40 (d, 1 H, $J_{3,4}$ = 3.2 Hz, H-4A), 5.11 (m, 1 H, H-3B), 4.97 (d, 1 H, $J_{1,2}$ = 8.5 Hz, H-1A), 4.92 (dd, 1 H, $J_{2,3}$ = 9.6 Hz, $J_{1,2}$ = 7.4 Hz, H-2B), 4.77 (dd, 1 H, $J_{2,3}$ = 11.0 Hz, H-3A), 4.68 (d, 1 H, $J_{1,2}$ = 7.4 Hz, H-1B), 4.23 (dd, 1 H, $J_{6a,6b}$ = 11.2 Hz, $J_{5,6a}$ = 5.5 Hz, H-6aA), 4.13 (dd, 1 H, $J_{5,6b}$ = 7.1 Hz, H-6bA), 3.94 (m, 3 H, H-4B, H-5B, CH isopropyl), 3.87 (s, 3 H, COOMe), 3.53 (m, 1 H, H-2A), 3.14 (bs, 1 H, OH), 2.70-2.43 (m, 4 H, $CH_2CH_2C_8F_{17}$), 2.13 (s, 3 H, CH_3 (Ac)), 1.25 (d, 3 H, $^3J_{H,H}$ = 6.2 Hz, CH_3 (isopropyl)), 1.19, 1.15 (s, 18 H, CH_3 (Piv)), 1.13 (d, 3 H, $^3J_{H,H}$ = 6.2 Hz, CH_3 (isopropyl)). ^{13}C NMR (100.6 MHz, $CDCl_3$) δ 178.1, 176.5, 170.6, 170.0, 168.9 (CO, Piv, COOMe, Ac, $COCH_2CH_2C_8F_{17}$), 157.7 (q, $COCF_3$), 115.4 (q, $COCF_3$), 100.0 (C-1B), 97.4 (C-1A), 74.1 (C-4B, C-5A, C-5B or CH isopropyl), 73.5 (C-3B), 72.9 (C-4B, C-5A, C-5B or CH isopropyl), 71.8 (C-3A), 71.1 (C-4B, C-5A, C-5B or CH isopropyl), 70.6 (C-2B), 70.2 (C-4B, C-5A, C-5B or CH isopropyl), 69.1 (C-4A), 62.8 (C-6A), 55.7 (C-2A), 52.9 ($COOCH_3$), 38.8, 38.7 ($C(CH_3)_3$, Piv), 27.0, 26.9 (CH_3, Piv), 26.4 ($CH_2CH_2C_8F_{17}$), 25.3 (t, $CH_2CH_2C_8F_{17}$), 23.2, 21.6 (CH_3, isopropyl), 20.6 (CH_3, Ac). ^{19}F NMR (376 MHz, $CDCl_3$): *δ* -75.93 (s, 3F), -80.77 (t, 3F), -114.71 (m, 2F), -121.87 (m, 6F), -122.71 (m, 2F), -123.44 (m, 2F), -126.12 (m, 2F). HR MS: *m/z*: calcd. for $C_{41}H_{49}O_{16}NF_{20}Na$: 1214.2624; found: 1214.2612 $[M + Na]^+$.

2-Propyl *O*-(methyl 4-*O*-levulinyl-2,3-di-*O*-pivaloyl-β-D-glucopyranosyluronate)-(1→3)-*O*-(4,6-di-*O*-acetyl-2-deoxy-2-trifluoroacetamido-α,β-D-galactopyranosyl)-(1→4)-*O*-(methyl 2,3-di-*O*-pivaloyl-β-D-glucopyranosyluronate)-(1→3)-4-*O*-acetyl-2-deoxy-6-*O*-4,4,5,5,6,6,7,7,8,8,9,9,10,10,11,11,11-heptadecafluoroundecanoyl-2-trifluoroacetamido-β-D-

galactopyranoside (14α/β). In a manner similar to a procedure from [3], **1** (58 mg, 0.060 mmol) and **2** (40 mg, 0.034 mmol) were combined in a flask, coevaporated with toluene and dried under vacuum. The starting materials were dissolved in dry CH_2Cl_2 (2 mL) and further dried by stirring over freshly activated 4 Å molecular sieves for 15 min. TMSOTf (92 µL of a 0.13 M solution in CH_2Cl_2, 0.012 mmol) was added at 0 ºC. After 30 min at 0 ºC, the reaction was quenched with Et_3N (1 mL) and filtered, and the solvent was removed under reduced pressure. The residue was first purified by fluorous solid-phase extraction. Fluorophobic wash was carried out with 4:1 MeOH-H_2O. Fluorophilic elution with acetone gave a mixture of the α-β tetrasaccharides and unreacted acceptor. Standard silica gel column chromatography using 1:2 Et_2O/CH_2Cl_2 as eluent afforded pure **14α** (17 mg, 25%) and **14β** (22 mg, 33%) as colourless oils, together with recovered acceptor **2** (10 mg, 25%).

14α: R_f 0.54 (1:1 EtOAc-toluene). $[\alpha]_D$ +8º (*c* 1.0, CH_2Cl_2). ^{1}H NMR (400 MHz, $CDCl_3$) δ 6.94 (d, 1 H, $J_{2,NH}$ = 8.2 Hz, NH), 6.76 (d, 1 H, $J_{2,NH}$ = 7.6 Hz, NH), 5.44 (d, 1 H, $J_{3,4}$ = 3.5 Hz, H-4A), 5.40 (m, 1 H, H-4C), 5.28 (m, 2 H, H-3D, H-4D), 5.18 (m, 1 H, H-3B), 5.12 (d, 1 H, $J_{1,2}$ = 3.2 Hz, H-1C), 5.08 (m, 1 H, H-2D), 4.91 (dd, 1 H, $J_{2,3}$ = 8.5 Hz, $J_{1,2}$ = 7.2 Hz, H-2B), 4.89 (d, 1 H, $J_{1,2}$ = 8.2 Hz, H-1A), 4.80 (d, 1 H, $J_{1,2}$ = 7.7 Hz, H-1D), 4.76 (dd, 1 H, H-3A), 4.72 (d, 1 H, H-1B), 4.50 (m, 1 H, H-2C), 4.32 (dd, 1 H, $J_{4,5}$ = 9.0 Hz, $J_{3,4}$ = 7.7 Hz, H-4B), 4.29-4.13 (m, 4 H, H-3C, H-6aA, H-6bA, H-6aC), 4.16 (d, 1 H, H-5B), 4.11 (d, 1 H, $J_{4,5}$ = 9.5 Hz, H-5D), 3.97 (m, 3 H, H-5A, H-6bC, CH isopropyl), 3.91 (s, 3 H, COOMe), 3.87 (m, 1 H, H-5C), 3.81 (s, 3 H, COOMe), 3.58 (m, 1 H, H-2A), 2.71-2.44 (m, 8 H, CH_2 (Lev), $CH_2CH_2C_8F_{17}$), 2.20 (s, 3 H, CH_3 (Lev)), 2.14, 2.13, 2.12 (s, 9 H, CH_3 (Ac)), 1.25 (d, 3 H, $^3J_{H,H}$ = 6.2 Hz, CH_3 (isopropyl)), 1.16, 1.15, 1.14, 1.13 (s, 36 H, CH_3 (Piv)), 1.13 (d, 3 H, $^3J_{H,H}$ = 6.2 Hz, CH_3 (isopropyl)). ^{13}C NMR (100.6 MHz, $CDCl_3$) δ 205.7 (CO, Lev), 177.0-167.0 (CO, Lev, Piv, COOMe, Ac, *C*O$CH_2CH_2C_8F_{17}$), 157.7 (q, *C*OCF_3), 115.4 (q, CO*C*F_3), 99.1 (C-1B), 98.3 (C-1D), 97.6 (C-1A), 96.3 (C-1C), 74.3 (C-3B), 73.9 (C-5B), 72.9 (C-5A or CH isopropyl), 72.2 (C-4B, C-5D), 71.4, 71.2 (C-3A, C-3D), 71.1 (C-2B, C-5A or CH isopropyl), 69.5, 69.4 (C-2D, C-4D), 68.9, 68.8, 68.6 (C-3C, C-4A, C-5C), 68.3 (C-4C), 62.9 (C-6A), 61.2 (C-6C), 55.6 (C-2A), 53.0, 52.9 (COO*C*H_3), 49.0 (C-2C), 38.8, 38.7 (*C*$(CH_3)_3$, Piv), 37.4 (*C*H_2COO, Lev), 29.7 (CH_3, Lev), 27.5 (*C*H_2CH_2COO, Lev), 27.0, 26.9 (CH_3, Piv), 26.4 (*C*$H_2CH_2C_8F_{17}$), 25.3 (t, *C*$H_2C_8F_{17}$), 23.2, 21.6 (CH_3, isopropyl), 20.7, 20.6, 20.5 (CH_3, Ac). ^{19}F NMR (376 MHz, $CDCl_3$): *δ* -75.62 (s, 3F), -75.93 (s, 3F), -80.72 (t, 3F), -114.73 (m, 2F), -121.87 (m, 6F), -122.68 (m, 2F), -123.42 (m, 2F), -126.05 (m, 2F). HR MS: *m/z*: calcd. for $C_{75}H_{95}O_{33}N_2F_{23}Na$: 2011.5342; found: 2011.5342 $[M + Na]^+$.

14β: R_f 0.46 (1:1 EtOAc-toluene). $[\alpha]_D$ -2º (*c* 1.0, CH_2Cl_2). ^{1}H NMR (400 MHz, $CDCl_3$) δ 6.96 (d, 1 H, $J_{2,NH}$ = 7.6 Hz, NH), 6.92 (d, 1 H, $J_{2,NH}$ = 6.5 Hz, NH), 5.47 (d, 1 H, $J_{3,4}$ = 3.1 Hz, H-4A or H-4C), 5.42 (d, 1 H, $J_{3,4}$ = 3.4 Hz, H-4A or H-4C), 5.23 (m, 2 H, H-3D, H-4D), 5.12 (m, 1 H, H-3B), 4.96 (m, 1 H, H-2D), 4.94 (d, 1 H, $J_{1,2}$ = 8.2 Hz, H-1A or H-1C), 4.89 (dd, 1 H,

$J_{2,3}$ = 8.5 Hz, $J_{1,2}$ = 7.2 Hz, H-2B), 4.78 (d, 1 H, $J_{1,2}$ = 8.5 Hz, H-1A or H-1C), 4.71 (m, 1 H, H-3A or H-3C), 4.69 (d, 1 H, H-1B), 4.67 (m, 1 H, H-3A or H-3C), 4.64 (d, 1 H, $J_{1,2}$ = 7.5 Hz, H-1D), 4.28 (m, 1 H, H-4B), 4.22 (dd, 1 H, $J_{6a,6b}$ = 11.1 Hz, $J_{5,6a}$ = 5.4 Hz, H-6aA or H-6aC), 4.15-4.04 (m, 3 H, H-6aA or H-6aC, H-6bA, H-6bC), 4.02 (m, 1 H, H-5D), 3.92 (m, 4 H, H-5A, H-5B, H-5C, CH isopropyl), 3.86, 3.78 (s, 6 H, COOMe), 3.52, 3.43 (m, 2 H, H-2A, H-2C), 2.70-2.43 (m, 8 H, CH_2 (Lev), $CH_2CH_2C_8F_{17}$), 2.18 (s, 3 H, CH_3 (Lev)), 2.13, 2.12, 2.04 (s, 9 H, CH_3 (Ac)), 1.25 (d, 3 H, $^3J_{H,H}$ = 6.2 Hz, CH_3 (isopropyl)), 1.18, 1.14 (s, 36 H, CH_3 (Piv)), 1.12 (d, 3 H, $^3J_{H,H}$ = 6.2 Hz, CH_3 (isopropyl)). ^{13}C NMR (100.6 MHz, $CDCl_3$) δ 205.7 (CO, Lev), 177.6-166.9 (CO, Lev, Piv, COOMe, Ac, *C*O$CH_2CH_2C_8F_{17}$), 157.7 (q, *C*OCF_3), 115.4 (q, CO*C*F_3), 99.9 (C-1B), 99.4 (C-1D), 97.3, 97.2 (C-1A, C-1C), 74.1 (C-5A or C-5B or C-5C or CH isopropyl), 73.4 (C-4B), 72.9 (C-5A or C-5B or C-5C or CH isopropyl), 72.2 (C-5D), 71.5-71.1 (C-3A, C-3B, C-3C, C-2B, C-3D or C-4D, C-5A or C-5B or C-5C or CH isopropyl), 70.6 (C-2D), 69.4 (C-3D or C-4D), 68.8, 68.6 (C-4A, C-4C), 62.9, 61.4 (C-6A, C-6C), 55.7, 55.0 (C-2A, C-2C), 53.3, 53.0 (COO*C*H_3), 38.7, 38.6 (*C*$(CH_3)_3$, Piv), 37.4 (*C*H_2COO, Lev), 29.7 (CH_3, Lev), 27.6 (*C*H_2CH_2COO, Lev), 27.1, 27.0, 26.9 (CH_3, Piv), 26.2 (*C*$H_2CH_2C_8F_{17}$), 25.4 (t, *C*$H_2C_8F_{17}$), 23.2, 21.6 (CH_3, isopropyl), 20.7, 20.6, 20.4 (CH_3, Ac). ^{19}F NMR (376 MHz, $CDCl_3$): *δ* -75.85 (s, 3F), -75.89 (s, 3F), -80.74 (t, 3F), -114.72 (m, 2F), -121.89 (m, 6F), -122.68 (m, 2F), -123.42 (m, 2F), -126.11 (m, 2F). HR MS: *m/z*: calcd. for $C_{75}H_{95}O_{33}N_2F_{23}Na$: 2011.5342; found: 2011.5342 $[M + Na]^+$.

4-Methoxyphenyl 3-*O*-(sodium β-D-glucopyranosyluronate)-2-acetamido-2-deoxy-β-D-galactopyranoside (15). In a manner similar to a procedure from [4], H_2O_2 (30%, 1.2 mL) and an aqueous solution of LiOH (0.7 M, 0.72 mL) were added at −5 ºC to a solution of **6** (25 mg, 0.030 mmol) in THF (1 mL). After stirring for 24 h at room temperature, MeOH (2 mL) and an aqueous solution of NaOH (4 M, 0.76 mL) were added. After 72 h at room temperature, the mixture was neutralized with Amberlite IR-120 (H^+) resin and then, a drop of Et_3N was added. The mixture was filtered, concentrated and coevaporated several times with MeOH. Et_3N (55 µL) and Ac_2O (60 µL) were added at 0 ºC to a solution of the reaction crude in MeOH (3 mL). After stirring for 2 h at room temperature, Et_3N (1 mL) was added and the mixture was concentrated to dryness. The residue was purified by Sephadex G-10 (9:1 H_2O-MeOH), Sephadex LH-20 (MeOH) and by silica gel column chromatography (14:5:3 EtOAc/MeOH/H_2O) to give **15** as a colourless amorphous solid. The sodium salt of **15** (6 mg, 38%) was obtained by treatment with sodium Dowex 50 WX2. R_f 0.17 (14:5:3 EtOAc-MeOH-H_2O). ^{1}H NMR (400 MHz, CD_3OD) δ 7.01 (d, 2 H, $^3J_{H,H}$ = 9.0 Hz, OPMP), 6.84 (d, 2 H, $^3J_{H,H}$ = 9.0 Hz, OPMP), 4.96 (d, 1 H, $J_{1,2}$ = 8.5 Hz, H-1A), 4.43 (d, 1 H, $J_{1,2}$ = 7.9 Hz, H-1B), 4.30 (dd, 1 H, $J_{2,3}$ = 10.6 Hz, H-2A), 4.23 (d, 1 H, $J_{3,4}$ = 3.0 Hz, H-4A), 3.89 (dd, 1 H, H-3A), 3.82 (m, 2 H, H-6aA, H-6bA), 3.76 (s, 3 H, OPMP), 3.68 (m, 1 H, H-5A), 3.59 (d, 1 H, $J_{4,5}$ = 9.3 Hz, H-5B), 3.41 (m, 2 H, H-3B, H-4B), 3.30 (m, 1 H, H-2B), 2.00 (s, 3 H, NHAc). ^{13}C NMR (100.6

MHz, CD_3OD) δ 175.9 (CO), 173.0 (CO, amide), 155.3, 151.9 (Ar-C), 117.8, 114.1 (Ar-CH), 104.4 (C-1B), 100.9 (C-1A), 80.3 (C-3A), 76.1 (C-3B or C-4B), 75.4 (C-5A), 74.7 (C-5B), 73.3 (C-2B), 72.2 (C-3B or C-4B), 67.7 (C-4A), 61.4 (C-6A), 54.6 (OPMP), 51.6 (C-2A), 21.8 (CH_3, NHAc). ESI MS: *m/z*: calcd. for $C_{21}H_{28}O_{13}N$: 502.2; found: 501.9 $[M - Na]^-$.

2-Propyl *O*-(sodium β-D-glucopyranosyluronate)-(1→3)-*O*-(2-acetamido-2-deoxy-α-D-galactopyranosyl)-(1→4)-*O*-(sodium β-D-glucopyranosyluronate)-(1→3)-2-acetamido-2-deoxy-β-D-galactopyranoside (16α). H_2O_2 (30%, 1.2 mL) and an aqueous solution of LiOH (0.7 M, 0.73 mL) were added at −5 ºC to a solution of **14α** (30 mg, 0.015 mmol) in THF (1 mL). After stirring for 24 h at room temperature, MeOH (2 mL) and an aqueous solution of NaOH (4 M, 0.76 mL) were added. After 72 h at room temperature, the mixture was neutralized with Amberlite IR-120 (H^+) resin and then, a drop of Et_3N was added. The residue was filtered, concentrated and coevaporated several times with MeOH. Et_3N (90 µL) and Ac_2O (100 µL) were added at 0 ºC to a solution of the reaction crude in MeOH (4 mL). After stirring for 2 h at room temperature, Et_3N (1 mL) was added and the mixture was concentrated to dryness. The residue was purified by Sephadex LH-20 (MeOH and 9:1 H_2O/MeOH) and silica gel column chromatography (14:5:3 EtOAc/MeOH/H_2O) to give **16α** as a colourless amorphous solid. The sodium salt of **16α** (4 mg, 30%) was obtained by treatment with sodium Dowex 50 WX2 resin. R_f 0.34 (6:5:3 EtOAc-MeOH-H_2O). ^1H NMR (500 MHz, D_2O) δ 5.34 (d, 1 H, $J_{1,2}$ = 3.8 Hz, H-1C), 4.49 (d, 1 H, $J_{1,2}$ = 8.5 Hz, H-1A), 4.48 (d, 1 H, $J_{1,2}$ = 7.9 Hz, H-1B or H-1D), 4.43 (d, 1 H, $J_{1,2}$ = 7.9 Hz, H-1B or H-1D), 4.25 (dd, 1 H, $J_{2,3}$ = 11.0 Hz, H-2C), 4.14 (d, 1 H, $J_{3,4}$ = 2.6 Hz, H-4C), 4.04 (d, 1 H, $J_{3,4}$ = 2.9 Hz, H-4A), 3.96 (m, 1 H, CH isopropyl), 3.89-3.40 (m, 15 H, H-2A, H-3A, H-5A, H-6aA, H-6bA, H-3B, H-4B, H-5B, H-3C, H-5C, H-6aC, H-6bC, H-3D, H-4D, H-5D), 3.26 (m, 2 H, H-2B, H-2D), 1.95, 1.94 (s, 6 H, CH_3 (NHAc)), 1.13 (d, 3 H, $^3J_{H,H}$ = 6.4 Hz, CH_3 (isopropyl)), 1.06 (d, 3 H, $^3J_{H,H}$ = 6.4 Hz, CH_3 (isopropyl)). ^{13}C NMR (125.7 MHz, D_2O, selected data from HSQC experiment) δ 104.7, 104.2 (C-1B, C-1D), 100.9 (C-1A), 97.4 (C-1C), 81.1-69.6 (C-3A, C-5A, C-2B, C-3B, C-4B, C-5B, C-3C, C-5C, C-2D, C-3D, C-4D, C-5D, CH isopropyl), 68.7, 68.4 (C-4A, C-4C), 61.3 (C-6A, C-6C), 52.3 (C-2A), 49.0 (C-2C), 23.4 (CH_3, isopropyl), 23.0 (CH_3, NHAc), 22.0 (CH_3, isopropyl). HR MS: *m/z*: calcd. for $C_{31}H_{49}O_{23}N_2Na_2$: 863.2516; found: 863.2508 $[M + H]^+$.

2-Propyl *O*-(sodium β-D-glucopyranosyluronate)-(1→3)-*O*-(2-acetamido-2-deoxy-β-D-galactopyranosyl)-(1→4)-*O*-(sodium β-D-glucopyranosyluronate)-(1→3)-2-acetamido-2-deoxy-β-D-galactopyranoside (16β). H_2O_2 (30%, 1.2 mL) and an aqueous solution of LiOH (0.7 M, 0.7 mL) were added at −5 ºC to a solution of **14β** (22 mg, 0.011 mmol) in THF (1 mL). After stirring for 24 h at room temperature, MeOH (2 mL) and an aqueous solution of NaOH (4 M, 0.76 mL) were added. After 72 h at room temperature, the mixture was neutralized with Amberlite IR-120 (H^+) resin and then, a drop of Et_3N was added. The residue was filtered, concentrated and coevaporated several times with MeOH. Et_3N (80 µL) and Ac_2O (94 µL) were

added at 0 ºC to a solution of the reaction crude in MeOH (4 mL). After stirring for 2 h at room temperature, Et_3N (1 mL) was added and the mixture was concentrated to dryness. The residue was purified by Sephadex LH-20 (MeOH and 9:1 H_2O-MeOH) and silica gel column chromatography (14:5:3 EtOAc/MeOH/H_2O) to give **16β** as a colourless amorphous solid. The sodium salt of **16β** (7 mg, 73%) was obtained by treatment with sodium Dowex 50 WX2 resin. R_f 0.31 (6:5:3 EtOAc-MeOH-H_2O). ^1H NMR (500 MHz, D_2O) δ 4.48 (d, 1 H, $J_{1,2}$ = 8.6 Hz, H-1A), 4.43 (d, 1 H, $J_{1,2}$ = 7.8 Hz, H-1C), 4.41 (d, 1 H, $J_{1,2}$ = 7.5 Hz, H-1B or H-1D), 4.40 (d, 1 H, $J_{1,2}$ = 7.9 Hz, H-1B or H-1D), 4.09 (d, 1 H, $J_{3,4}$ = 3.2 Hz, H-4A or H-4C), 4.03 (d, 1 H, $J_{3,4}$ = 3.2 Hz, H-4A or H-4C), 3.96 (m, 1 H, CH isopropyl), 3.93 (m, 1 H, H-2C), 3.86 (dd, 1 H, $J_{2,3}$ = 11.0 Hz, $J_{1,2}$ = 8.6 Hz, H-2A), 3.75-3.38 (m, 14 H, H-3A, H-5A, H-6aA, H-6bA, H-3B, H-4B, H-5B, H-3C, H-5C, H-6aC, H-6bC, H-3D, H-4D, H-5D), 3.26 (m, 2 H, H-2B, H-2D), 1.95, 1.94 (s, 6 H, CH_3 (NHAc)), 1.12 (d, 3 H, $^3J_{H,H}$ = 6.2 Hz, CH_3 (isopropyl)), 1.05 (d, 3 H, $^3J_{H,H}$ = 6.2 Hz, CH_3 (isopropyl)). ^{13}C NMR (125.7 MHz, D_2O, selected data from HSQC experiment) δ 105.0-100.4 (C-1A, C-1B, C-1C, C-1D), 79.9-71.6 (C-3A, C-5A, C-2B, C-3B, C-4B, C-5B, C-3C, C-5C, C-2D, C-3D, C-4D, C-5D, CH isopropyl), 68.4 (C-4A, C-4C), 61.6 (C-6A, C-6C), 51.6, 50.7 (C-2A, C-2C), 22.3 (CH_3, isopropyl), 22.2 (CH_3, NHAc), 20.7 (CH_3, isopropyl). HR MS: *m/z*: calcd. for $C_{31}H_{49}O_{23}N_2Na_2$: 863.2516; found: 863.2506 $[M + H]^+$.

2-Propyl 3-*O*-(methyl 4-*O*-levulinyl-2,3-di-*O*-pivaloyl-β-D-glucopyranosyluronate)-4,6-di-*O*-acetyl-2-deoxy-2-trifluoroacetamido-β-D-galactopyranoside (17). **1** (200 mg, 0.208 mmol) was coevaporated with toluene, dried under vacuum, dissolved in dry CH_2Cl_2 (4 mL) and further dried by stirring over freshly activated 4 Å molecular sieves for 15 min. 2-propanol (400 µL of a 1.05 M solution in CH_2Cl_2, 0.417 mmol) was added. Then, TMSOTf (190 µL of a 0.22 M solution in CH_2Cl_2, 0.042 mmol) was added at 0 ºC. After 30 min at 0 ºC, the reaction was quenched with Et_3N (1 mL) and filtered, and the solvent was removed under reduced pressure. The residue was purified by column chromatography using 1:2→1:1 EtOAc/petroleum ether as eluent to give compound **17** as an amorphous white solid (130 mg, 73%). R_f 0.35 (2:1 EtOAc-petroleum ether). $[\alpha]_D$ +6º (*c* 0.94, CH_2Cl_2). ^1H NMR (400 MHz, $CDCl_3$) δ 6.73 (d, 1 H, $J_{2,NH}$ = 7.0 Hz, NH), 5.46 (d, 1 H, $J_{3,4}$ = 3.0 Hz, H-4A), 5.25 (m, 2 H, H-3B, H-4B), 4.97 (m, 1 H, H-2B), 4.89 (d, 1 H, $J_{1,2}$ = 8.5 Hz, H-1A), 4.69 (d, 1 H, $J_{1,2}$ = 7.5 Hz, H-1B), 4.68 (dd, 1 H, $J_{2,3}$ = 7.8 Hz, H-3A), 4.12 (dd, 1 H, $J_{6a,6b}$ = 11.7 Hz, $J_{5,6a}$ = 7.0 Hz, H-6aA), 4.10 (dd, 1 H, $J_{5,6b}$ = 6.7 Hz, H-6bA), 4.02 (m, 1 H, H-5B), 3.92 (m, 2 H, H-5A, CH (isopropyl)), 3.77 (s, 3 H, COOMe), 3.58 (m, 1 H, H-2A), 2.70-2.44 (m, 4 H, CH_2 (Lev)), 2.13 (s, 3 H, CH_3 (Lev)), 2.10, 2.07 (s, 6 H, CH_3 (Ac)), 1.23 (d, 3 H, $^3J_{H,H}$ = 6.2 Hz, CH_3 (isopropyl)), 1.12, 1.11 (s, 18 H, CH_3 (Piv)), 1.11 (m, 3 H, CH_3 (isopropyl)). ^{13}C NMR (100.6 MHz, $CDCl_3$) δ 205.8 (CO, Lev), 177.1, 176.2, 171.0, 170.6, 169.8, 166.9 (CO, Lev, Piv, COOMe, Ac), 157.7 (q, *C*OCF_3), 115.4 (q, CO*C*F_3), 99.4 (C-1B), 97.7 (C-1A), 72.9 (CH isopropyl), 72.3 (C-5B), 72.0 (C-3A), 71.4 (C-3B or C-4B), 71.2 (C-5A), 70.7 (C-2B), 69.4 (C-3B or C-4B), 68.8 (C-4A), 62.1 (C-6A), 55.4 (C-2A), 52.9

(COOCH_3), 38.8, 38.7 (*C*(CH_3)$_3$, Piv), 37.4 (*C*H_2COO, Lev), 29.8 (CH_3, Lev), 27.6 (*C*H_2CH_2COO, Lev), 26.9 (CH_3, Piv), 23.2, 21.7 (CH_3, isopropyl), 20.7 (CH_3, Ac). ^{19}F NMR (376 MHz, $CDCl_3$): δ -75.88 (s, 3F). HR MS: *m/z*: calcd. for $C_{37}H_{54}O_{18}NF_3Na$: 880.3185; found: 880.3205 $[M + Na]^+$.

2-Propyl 3-*O*-(methyl 2,3-di-*O*-pivaloyl-β-D-glucopyranosyluronate)-4,6-di-*O*-acetyl-2-deoxy-2-trifluoroacetamido-β-D-galactopyranoside (18). In a manner similar to a procedure from [3], **17** (130 mg, 0.151 mmol) was dissolved in CH_2Cl_2 (3 mL) and hydrazine monohydrate (1.5 mL of a 0.25 M solution in 3:2 Py/AcOH) was added. After stirring at room temperature for 1 h, the reaction mixture was quenched with acetone (1 mL), diluted with CH_2Cl_2, washed with 1 N HCl, aqueous saturated $NaHCO_3$ and H_2O, dried with $MgSO_4$, filtered and concentrated in vacuo. The residue was purified by column chromatography using 1:1 → 2:1 EtOAc/petroleum ether as eluent to give **18** as a colourless oil (102 mg, 89%). R_f 0.47 (2:1 EtOAc/petroleum ether). $[\alpha]_D$ -39° (*c* 1.1, CH_2Cl_2). ^{1}H NMR (400 MHz, $CDCl_3$) δ 6.75 (d, 1 H, $J_{2,NH}$ = 7.0 Hz, NH), 5.48 (d, 1 H, $J_{3,4}$ = 3.5 Hz, H-4A), 5.10 (m, 1 H, H-3B), 4.94 (d, 1 H, $J_{1,2}$ = 8.5 Hz, H-1A), 4.91 (dd, 1 H, $J_{2,3}$ = 9.6 Hz, $J_{1,2}$ = 7.2 Hz, H-2B), 4.73 (dd, 1 H, $J_{2,3}$ = 10.9 Hz, H-3A), 4.68 (d, 1 H, $J_{1,2}$ = 7.2 Hz, H-1B), 4.17-4.07 (m, 2 H, H-6aA, H-6bA), 3.98-3.90 (m, 4 H, H-5A, H-4B, H-5B, CH isopropyl), 3.87 (s, 3 H, COOMe), 3.54 (ddd, 1 H, H-2A), 3.23 (bs, 1 H, OH), 2.08, 2.06 (s, 6 H, CH_3 (Ac)), 1.25 (d, 3 H, $^3J_{H,H}$ = 6.1 Hz, CH_3 (isopropyl)), 1.18, 1.14 (s, 18 H, CH_3 (Piv)), 1.13 (d, 3 H, $^3J_{H,H}$ = 6.1 Hz, CH_3 (isopropyl)). ^{13}C NMR (100.6 MHz, $CDCl_3$) δ 178.1, 176.5, 170.6, 17.0, 169.0 (CO, Piv, COOMe, Ac), 157.7 (q, *C*OCF_3), 115.4 (q, CO*C*F_3), 100.0 (C-1B), 97.4 (C-1A), 74.2 (C-4B or C-5A or C-5B or CH isopropyl), 73.5 (C-3B), 72.9 (C-4B or C-5A or C-5B or CH isopropyl), 71.9 (C-3A), 71.2 (C-4B or C-5A or C-5B or CH isopropyl), 70.7 (C-2B), 70.2 (C-4B or C-5A or C-5B or CH isopropyl), 69.1 (C-4A), 62.3 (C-6A), 55.7 (C-2A), 52.9 (COO*C*H_3), 38.8, 38.7 (*C*(CH_3)$_3$, Piv), 27.0, 26.9 (CH_3, Piv), 23.2, 21.7 (CH_3, isopropyl), 20.7, 20.6 (CH_3, Ac). HR MS: *m/z*: calcd. for $C_{32}H_{48}O_{16}NF_3Na$: 782.2817; found: 782.2824 $[M + Na]^+$.

2-Propyl *O*-(methyl 4-*O*-levulinyl-2,3-di-*O*-pivaloyl-β-D-glucopyranosyluronate)-(1→3)-*O*-(4,6-di-*O*-acetyl-2-deoxy-2-trifluoroacetamido-α,β-D-galactopyranosyl)-(1→4)-*O*-(methyl 2,3-di-*O*-pivaloyl-β-D-glucopyranosyluronate)-(1→3)-4,6-di-*O*-acetyl-2-deoxy-2-trifluoroacetamido-β-D-galactopyranoside (19α/β).

Reaction conditions 1: **1** (143 mg, 0.149 mmol) and **18** (63 mg, 0.083 mmol) were combined in a flask, coevaporated with toluene and dried under vacuum. The starting materials were dissolved in dry CH_2Cl_2 (3 mL) and further dried by stirring over freshly activated 4 Å molecular sieves for 15 min. TMSOTf (185 µL of a 0.161 M solution in CH_2Cl_2, 0.03 mmol) was added at 0 °C. After 30 min at 0 °C, the reaction was quenched with Et_3N (1 mL) and filtered, and the solvent was removed under reduced pressure. The residue was purified by

column chromatography using 1:4 EtOAc/Et_2O and 15:1→12:1→9:1 CH_2Cl_2/acetone as eluents to give compounds **19α** (24 mg, 18%) and **19β** (68 mg, 53%) as colourless oils.

Reaction conditions 2: **1** (70 mg, 0.073 mmol) and **18** (40 mg, 0.053 mmol) were combined in a flask, coevaporated with toluene and dried under vacuum. The starting materials were dissolved in dry CH_3CN (2 mL) and further dried by stirring over freshly activated 4 Å molecular sieves for 15 min. TMSOTf (91 µL of a 0.16 M solution in CH_3CN, 0.015 mmol) was added at −20 ºC. After 30 min at −20ºC, the reaction was quenched with Et_3N (1 mL) and filtered, and the solvent was removed under reduced pressure. The residue was purified by column chromatography using 1:1 CH_2Cl_2/Et_2O and 1:5 EtOAc/Et_2O as eluents to give compounds **19α** (8 mg, 10%) and **19β** (33 mg, 40%).

19α: R_f 0.50 (1:3 EtOAc/Et_2O). $[\alpha]_D$ +92º (*c* 1.0, CH_2Cl_2). ^{1}H NMR (400 MHz, $CDCl_3$) δ 6.65 (d, 1 H, $J_{2,NH}$ = 8.7 Hz, NH), 6.65 (d, 1 H, $J_{2,NH}$ = 6.9 Hz, NH), 5.46 (d, 1 H, $J_{3,4}$ = 3.3 Hz, H-4A), 5.40 (m, 1 H, H-4C), 5.30 (m, 1 H, H-3D), 5.26 (m, 1 H, H-4D), 5.18 (m, 1 H, H-3B), 5.12 (d, 1 H, $J_{1,2}$ = 3.3 Hz, H-1C), 5.08 (m, 1 H, H-2D), 4.91 (d, 1 H, $J_{1,2}$ = 8.1 Hz, H-1A), 4.89 (m, 1 H, H-2B), 4.79 (d, 1 H, $J_{1,2}$ = 7.6 Hz, H-1D), 4.76 (dd, 1 H, $J_{2,3}$ = 11.0 Hz, H-3A), 4.71 (d, 1 H, $J_{1,2}$ = 7.2 Hz, H-1B), 4.51 (m, 1 H, H-2C), 4.33 (m, 1 H, H-4B), 4.26 (dd, 1 H, $J_{2,3}$ = 11.0 Hz, $J_{3,4}$ = 3.9 Hz, H-3C), 4.25-4.09 (m, 5 H, H-5B, H-5D, H-6aA, H-6bA, H-6aC), 4.00-3.88 (m, 7 H, H-5A, H-5C, H-6bC, CH isopropyl, COOMe), 3.80 (s, 3 H, COOMe), 3.54 (m, 1 H, H-2A), 2.78-2.46 (m, 4 H, CH_2 (Lev)), 2.20 (s, 3 H, CH_3 (Lev)), 2.14, 2.12, 2.11, 2.08 (s, 12 H, CH_3 (Ac)), 1.26 (d, 3 H, $^3J_{H,H}$ = 6.2 Hz, CH_3 (isopropyl)), 1.16, 1.15, 1.14, 1.13 (s, 36 H, CH_3 (Piv)), 1.13 (m, 3 H, CH_3 (isopropyl)). ^{13}C NMR (100.6 MHz, $CDCl_3$) δ 205.7 (CO, Lev), 177.0-167.0 (CO, Lev, Piv, COOMe, Ac), 157.5 (q, *C*OCF_3), 117.0 (q, CO*C*F_3), 99.1 (C-1B), 98.3 (C-1D), 97.5 (C-1A), 96.2 (C-1C), 74.3 (C-3B), 73.9 (C-5B), 72.9 (C-5A or CH isopropyl), 72.2, 72.1 (C-4B, C-5D), 71.4-71.2 (C-2B, C-3A, C-3D, C-5A or CH isopropyl), 69.6 (C-2D), 69.4 (C-4D), 69.0 (C-3C), 68.7, 68.6 (C-4A, C-5C), 68.2 (C-4C), 62.2 (C-6A), 61.3 (C-6C), 55.8 (C-2A), 53.0, 52.9 (COO*C*H_3), 49.0 (C-2C), 38.8, 38.7 (*C*$(CH_3)_3$, Piv), 37.4 (*C*H_2COO, Lev), 29.7 (CH_3, Lev), 27.5 (*C*H_2CH_2COO, Lev), 27.0, 26.9 (CH_3, Piv), 23.2, 21.7 (CH_3, isopropyl), 20.8, 20.7, 20.6, (CH_3, Ac). HR MS: *m/z*: calcd. for $C_{66}H_{94}F_6N_2O_{33}Na$: 1579.5535; found: 1579.5526 $[M + Na]^+$.

19β: R_f 0.42 (1:3 EtOAc/Et_2O). $[\alpha]_D$ +7º (*c* 1.0, CH_2Cl_2). ^{1}H NMR (400 MHz, $CDCl_3$) δ 6.77 (d, 1 H, $J_{2,NH}$ = 7.1 Hz, NH), 6.54 (d, 1 H, $J_{2,NH}$ = 7.1 Hz, NH), 5.47 (d, 1 H, $J_{3,4}$ = 3.1 Hz, H-4A or H-4C), 5.44 (d, 1 H, $J_{3,4}$ = 3.3 Hz, H-4A or H-4C), 5.23 (m, 2 H, H-3D, H-4D), 5.12 (m, 1 H, H-3B), 4.96 (d, 1 H, $J_{1,2}$ = 8.4 Hz, H-1A or H-1C), 4.94 (m, 1 H, H-2D), 4.87 (dd, 1 H, $J_{2,3}$ = 8.7 Hz, $J_{1,2}$ = 7.1 Hz, H-2B), 4.81 (d, 1 H, $J_{1,2}$ = 8.4 Hz, H-1A or H-1C), 4.72 (dd, 1 H, $J_{2,3}$ = 11.1 Hz, H-3A or H-3C), 4.69 (d, 1 H, H-1B), 4.65 (d, 1 H, $J_{1,2}$ = 7.7 Hz, H-1D), 4.63 (dd, 1 H, $J_{2,3}$ = 11.1 Hz, H-3A or H-3C), 4.29 (t, 1 H, $J_{3,4}$ = $J_{4,5}$ = 9.1 Hz, H-4B), 4.17-4.01 (m, 5 H, H-5D, H-6aA, H-6bA, H-6aC, H-6bC), 3.97-3.88 (m, 4 H, H-5A, H-5B, H-5C, CH isopropyl),

3.87, 3.78 (s, 6 H, COOMe), 3.49 (m, 2 H, H-2A, H-2C), 2.76-2.44 (m, 4 H, CH_2 (Lev)), 2.19 (s, 3 H, CH_3 (Lev)), 2.14, 2.12, 2.08, 2.04 (s, 12 H, CH_3 (Ac)), 1.26 (d, 3 H, $^3J_{H,H}$ = 6.5 Hz, CH_3 (isopropyl)), 1.18, 1.15, 1.12 (s, 36 H, CH_3 (Piv)), 1.12 (m, 3 H, CH_3 (isopropyl)). ^{13}C NMR (100.6 MHz, $CDCl_3$) δ 205.7 (CO, Lev), 177.5-166.9 (CO, Lev, Piv, COOMe, Ac), 157.5 (q, *C*OCF_3), 117.0 (q, CO*C*F_3), 99.9 (C-1B), 99.4 (C-1D), 97.3, 97.2 (C-1A, C-1C), 74.1 (C-5A or C-5B or C-5C or CH isopropyl), 73.4 (C-4B), 72.9-69.4 (C-2B, C-2D, C-3A, C-3B, C-3C, C-3D, C-4D, C-5A or C-5B or C-5C or CH isopropyl, C-5D), 68.8, 68.6 (C-4A, C-4C), 62.3, 61.4 (C-6A, C-6C), 55.9, 54.9 (C-2A, C-2C), 53.3, 52.9 (COO*C*H_3), 38.7, 38.6 (*C*$(CH_3)_3$, Piv), 37.5 (*C*H_2COO, Lev), 29.7 (CH_3, Lev), 27.6 (*C*H_2CH_2COO, Lev), 27.1, 27.0, 26.9 (CH_3, Piv), 23.2, 21.7 (CH_3, isopropyl), 20.7, 20.6, 20.4 (CH_3, Ac). HR MS: *m/z*: calcd. for $C_{66}H_{94}F_6N_2O_{33}Na$: 1579.5535; found: 1579.5523 $[M + Na]^+$.

2-Propyl *O*-(methyl 2,3-di-*O*-pivaloyl-β-D-glucopyranosyluronate)-(1→3)-*O*-(4,6-di-*O*-acetyl-2-deoxy-2-trifluoroacetamido-β-D-galactopyranosyl)-(1→4)-*O*-(methyl 2,3-di-*O*-pivaloyl-β-D-glucopyranosyluronate)-(1→3)-4,6-di-*O*-acetyl-2-deoxy-2-trifluoroacetamido-β-D-galactopyranoside (20). In a manner similar to a procedure from [3], **19β** (68 mg, 0.044 mmol) was dissolved in CH_2Cl_2 (2 mL) and hydrazine monohydrate (0.44 mL of a 0.25 M solution in 3:2 Py/AcOH) was added. After stirring at room temperature for 1 h, the reaction mixture was quenched with acetone (1 mL), diluted with CH_2Cl_2, washed with 1 N HCl, aqueous saturated $NaHCO_3$ and H_2O, dried with $MgSO_4$, filtered and concentrated in vacuo. The residue was purified by column chromatography using 2:1 EtOAc/petroleum ether as eluent to give **20** as a colourless oil (35 mg, 55%). R_f 0.42 (2:1 EtOAc/petroleum ether). $[\alpha]_D$ -44° (*c* 1.0, CH_2Cl_2). ^{1}H NMR (400 MHz, $CDCl_3$) δ 6.89 (d, 1 H, $J_{2,NH}$ = 7.1 Hz, NH), 6.62 (d, 1 H, $J_{2,NH}$ = 7.3 Hz, NH), 5.49 (d, 1 H, $J_{3,4}$ = 3.4 Hz, H-4A or H-4C), 5.42 (d, 1 H, $J_{3,4}$ = 3.2 Hz, H-4A or H-4C), 5.17 (t, 1 H, $J_{2,3}$ = $J_{3,4}$ = 9.1 Hz, H-3B), 5.10 (t, 1 H, $J_{2,3}$ = $J_{3,4}$ = 9.1 Hz, H-3D), 4.92 (d, 1 H, $J_{1,2}$ = 8.3 Hz, H-1A or H-1C), 4.89 (m, 2 H, H-2B, H-2D), 4.84 (d, 1 H, $J_{1,2}$ = 8.1 Hz, H-1A or H-1C), 4.71 (d, 1 H, $J_{1,2}$ = 7.3 Hz, H-1B), 4.69 (m, 1 H, H-3A or H-3C), 4.67 (dd, 1 H, $J_{2,3}$ = 11.1 Hz, $J_{3,4}$ = 3.4 Hz, H-3A or H-3C), 4.62 (d, 1 H, $J_{1,2}$ = 7.5 Hz, H-1D), 4.29 (t, 1 H, $J_{3,4}$ = $J_{4,5}$ = 9.1 Hz, H-4B), 4.17-4.03 (m, 4 H, H-6aA, H-6aC, H-6bA or H-6bC), 3.98-3.89 (m, 6 H, H-4D, H-5A, H-5B, H-5C, H-5D, CH isopropyl), 3.86, 3.85 (s, 6 H, COOMe), 3.53, 3.36 (m, 2 H, H-2A, H-2C), 3.16 (d, 1 H, $J_{4,OH}$ = 3.1 Hz, OH), 2.13, 2.11, 2.07, 2.04 (s, 12 H, CH_3 (Ac)), 1.24 (d, 3 H, $^3J_{H,H}$ = 6.1 Hz, CH_3 (isopropyl)), 1.18, 1.14, 1.13 (s, 36 H, CH_3 (Piv)), 1.12 (m, 3 H, CH_3 (isopropyl)). ^{13}C NMR (100.6 MHz, $CDCl_3$) δ 178.2-168.4 (CO, Piv, COOMe, Ac), 157.8 (q, *C*OCF_3), 115.9 (q, CO*C*F_3), 100.0 (C-1B, C-1D), 97.4, 97.0 (C-1A, C-1C), 74.0, 73.9 (C-4D or C-5B or C-5D or CH isopropyl), 73.6 (C-3D), 73.3 (C-4B), 72.9 (C-4D or C-5B or C-5D or CH isopropyl), 72.3-70.4 (C-2B, C-2D, C-3A, C-3B, C-3C, C-5A, C-5C, C-4D or C-5B or C-5D or CH isopropyl), 68.9, 68.8 (C-4A, C-4C), 62.3, 61.6 (C-6A, C-6C), 55.7, 55.2 (C-2A, C-2C), 53.3, 52.9 (COO*C*H_3), 38.8, 38.7, 38.6 (*C*$(CH_3)_3$, Piv), 27.1, 27.0,

26.9 (CH_3, Piv), 23.2, 21.6 (CH_3, isopropyl), 20.7, 20.6, 20.4, (CH_3, Ac). HR MS: *m/z*: calcd. for $C_{61}H_{88}F_6N_2O_{31}Na$: 1481.5167; found: 1481.5155 $[M + Na]^+$.

2-Propyl *O*-(methyl 4-*O*-levulinyl-2,3-di-*O*-pivaloyl-β-D-glucopyranosyluronate)-(1→3)-*O*-(4,6-di-*O*-acetyl-2-deoxy-2-trifluoroacetamido-α,β-D-galactopyranosyl)-(1→4)-*O*-(methyl 2,3-di-*O*-pivaloyl-β-D-glucopyranosyluronate)-(1→3)-*O*-(4,6-di-*O*-acetyl-2-deoxy-2-trifluoroacetamido-β-D-galactopyranosyl)-(1→4)-*O*-(methyl 2,3-di-*O*-pivaloyl-β-D-glucopyranosyluronate)-(1→3)-4,6-di-*O*-acetyl-2-deoxy-2-trifluoroacetamido-β-D-galactopyranoside (21α/β). **1** (46 mg, 0.048 mmol) and **20** (35 mg, 0.024 mmol) were combined in a flask, coevaporated with toluene and dried under vacuum. The starting materials were dissolved in dry CH_2Cl_2 (2 mL) and further dried by stirring over freshly activated 4 Å molecular sieves for 15 min. TMSOTf (105 µL of a 0.092 M solution in CH_2Cl_2, 0.0096 mmol) was added at 0 ºC. After 30 min at 0 ºC, the reaction was quenched with Et_3N (1 mL) and filtered, and the solvent was removed under reduced pressure. The residue was purified by column chromatography using 100:1→80:1→60:1 CH_2Cl_2/MeOH as eluent to give compounds **21α** (10 mg, 18%) and **21β** (11 mg, 20%) as colourless oils and unreacted **20** (11 mg, 31%).

21α: R_f 0.34 (1:9 acetone/Et_2O). $[\alpha]_D$ +55º (*c* 0.83, CH_2Cl_2). ^1H NMR (400 MHz, $CDCl_3$) δ 6.81 (d, 1 H, $J_{2,NH}$ = 7.6 Hz, NH), 6.78 (d, 1 H, $J_{2,NH}$ = 8.7 Hz, NH), 6.45 (d, 1 H, $J_{2,NH}$ = 7.1 Hz, NH), 5.36, (m, 2 H, H-4A, H-4C) 5.31 (m, 1 H, H-4E), 5.19 (m, 2 H, H-3F, H-4F), 5.11-4.98 (m, 4 H, H-1E, H-2F, H-3B, H-3D), 4.89 (d, 1 H, $J_{1,2}$ = 8.3 Hz, H-1A or H-1C), 4.82-4.70 (m, 4 H, H-1F, H-1A or H-1C, H-2B, H-2D), 4.65 (dd, 1 H, $J_{2,3}$ = 10.9 Hz, $J_{3,4}$ = 3.3 Hz, H-3A or H-3C), 4.62-4.58 (m, 3 H, H-1B, H-1D, H-3A or H-3C), 4.42 (m, 1 H, H-2E), 4.27-3.71 (m, 25 H, H-3E, H-4B, H-4D, H-5A, H-5B, H-5C, H-5D, H-5E, H-5F, H-6aA, H-6bA, H-6aC, H-6bC, H-6aE, H-6bE, COOMe, CH (isopropyl)), 3.37 (m, 2 H, H-2A, H-2C), 2.60-2.38 (m, 4 H, CH_2 (Lev)), 2.12, 2.06, 2.05, 2.04, 2.03, 2.01, 1.96 (s, 21 H, CH_3 (Lev), CH_3 (Ac)), 1.18 (d, 3 H, $^3J_{H,H}$ = 6.3 Hz, CH_3 (isopropyl)), 1.10-1.06 (m, 57 H, CH_3 (Piv), CH_3 (isopropyl)). HR MS: *m/z*: calcd. for $C_{95}H_{134}O_{48}N_3F_9Na$: 2278.7883; found: 2278.7859 $[M + Na]^+$.

21β: R_f 0.33 (1:9 acetone/Et_2O). $[\alpha]_D$ -19º (*c* 0.92, CH_2Cl_2). ^1H NMR (400 MHz, $CDCl_3$) δ 6.71 (m, 2 H, NH), 6.43 (d, 1 H, $J_{2,NH}$ = 7.0 Hz, NH), 5.39, 5.36, 5.34 (3 t, 1 H, $J_{3,4}$ = 3.0 Hz, H-4A, H-4C, H-4E), 5.15 (m, 2 H, H-3F, H-4F), 5.03 (m, 2 H, H-3B, H-3D), 4.89 (m, 2 H, H-1A or H-1C or H-1E, H-2F), 4.80 (dd, 1 H, $J_{2,3}$ = 8.8 Hz, $J_{1,2}$ = 7.1 Hz, H-2B or H-2D), 4.75 (d, 1 H, $J_{1,2}$ = 8.3 Hz, H-1A or H-1C or H-1E), 4.74 (m, 1 H, H-2B or H-2D), 4.70 (d, 1 H, $J_{1,2}$ = 8.5 Hz, H-1A or H-1C or H-1E), 4.66 (dd, 1 H, $J_{4,5}$ = 11.1 Hz, $J_{3,4}$ = 3.0 Hz, H-3A or H-3C or H-3E), 4.61-4.51 (m, 5 H, H-1B, H-1D, H-1F, H-3A or H-3C or H-3E), 4.21, 4.20 (2 t, 2 H, $J_{3,4}$ = $J_{4,5}$ = 9.2 Hz, H-4B, H-4D), 4.09-3.93 (m, 7 H, H-5F, H-6aA, H-6bA, H-6aC, H-6bC, H-6aE, H-6bE), 3.91-3.79 (m, 12 H, H-5A, H-5B, H-5C, H-5D, H-5E, CH isopropyl, COOMe), 3.71 (s, 3 H, COOMe), 3.39 (m, 3 H, H-2A, H-2C, H-2E), 2.68-2.36 (m, 4 H, CH_2 (Lev)), 2.11-1.96 (s, 21 H, CH_3 (Lev), CH_3 (Ac)), 1.18 (d, 3 H, $^3J_{H,H}$ = 6.1 Hz, CH_3 (isopropyl)), 1.10-1.04 (m, 57 H,

CH_3 (Piv), CH_3 (isopropyl)). ^{13}C NMR (100.6 MHz, $CDCl_3$) δ 205.8 (CO, Lev), 177.4-166.8 (CO, Lev, Piv, COOMe, Ac), 157.7 (q, *C*OCF_3), 115.5 (q, CO*C*F_3), 100.0, 99.9, 99.4 (C-1B, C-1D, C-1F), 97.4, 97.1 (C-1A, C-1C, C-1E), 74.1-69.4 (C-2B, C-2D, C-2F, C-3A, C-3B, C-3C, C-3D, C-3E, C-3F, C-4B, C-4D, C-4F, C-5A, C-5B, C-5C, C-5D, C-5E, C-5F, CH isopropyl), 69.4-68.5 (C-4A, C-4C, C-4E), 62.2, 61.3 (C-6A, C-6C, C-6E), 55.9, 55.2, 54.9 (C-2A, C-2C, C-2E), 53.3, 52.9 (COO*C*H_3), 38.7 (*C*(CH_3)$_3$, Piv), 37.4 (*C*H_2COO, Lev), 29.7 (CH_3, Lev), 27.6 (*C*H_2CH_2COO, Lev), 27.0 (CH_3, Piv), 23.2, 21.7 (CH_3, isopropyl), 20.7, 20.5, (CH_3, Ac). HR MS: *m/z*: calcd. for $C_{95}H_{134}O_{48}N_3F_9Na$: 2278.7885; found: 2278.7864 $[M + Na]^+$.

Methyl (4-methoxyphenyl 4-*O*-levulinyl-2,3-di-*O*-pivaloyl-β-D-glucopyranoside) uronate (25). 3 (300 mg, 0.485 mmol) and 4-methoxyphenol (120 mg, 0.970 mmol) were dried under vacuum, dissolved in dry CH_2Cl_2 (6 mL) and further dried by stirring over freshly activated 4 Å molecular sieves for 15 min. TMSOTf (176 µL of a 0.28 M solution in CH_2Cl_2, 0.048 mmol) was added at 0 ºC. After 50 min at 0 ºC, the reaction was quenched with Et_3N (1 mL) and filtered, and the solvent was removed under reduced pressure. The residue was purified by column chromatography using 1:2 EtOAc/petroleum ether as eluent to give compound **25** as an amorphous solid (260 mg, 92%). R_f 0.38 (1:2 EtOAc/petroleum ether). $[\alpha]_D$ -86º (*c* 1.0, CH_2Cl_2). ^1H NMR (400 MHz, $CDCl_3$) δ 6.94 (d, 2 H, $^3J_{H,H}$ = 8.9 Hz, OPMP), 6.81 (d, 2 H, $^3J_{H,H}$ = 9.2 Hz, OPMP), 5.34 (m, 3 H, H-2, H-3, H-4), 5.01 (d, 1 H, $J_{1,2}$ = 7.8 Hz, H-1), 4.17 (m, 1 H, H-5), 3.77 (s, 6 H, COOMe, OPMP), 2.77-2.46 (m, 4 H, CH_2 (Lev)), 2.18 (s, 3 H, CH_3 (Lev)), 1.18, 1.16 (s, 18 H, CH_3 (Piv)). ^{13}C NMR (100.6 MHz, $CDCl_3$) δ 205.7 (CO, Lev), 177.2, 176.4, 171.1, 166.9 (CO, Lev, Piv, COOMe), 155.8-150.9 (Ar-C), 118.7-114.6 (Ar-CH), 100.7 (C-1), 72.6 (C-5), 71.3, 70.4, 69.5 (C-2, C-3, C-4), 55.6, 53.0 (COO*C*H_3, OPMP), 38.8 (*C*(CH_3)$_3$, Piv), 37.5 (*C*H_2COO, Lev), 29.7 (CH_3, Lev), 27.6 (*C*H_2CH_2COO, Lev), 27.0 (CH_3, Piv). HR MS: *m/z*: calcd. for $C_{29}H_{40}O_{12}Na$: 603.2412; found: 603.2402 $[M + Na]^+$.

Methyl (4-Methoxyphenyl 2,3-di-*O*-pivaloyl-β-D-glucopyranoside)uronate (26). 25 (260 mg, 0.444 mmol) was dissolved in CH_2Cl_2 (6 mL) and hydrazine monohydrate (1.1 mL of a 1 M solution in 3:2 Py/AcOH) was added. After stirring at room temperature for 1 h, the reaction mixture was quenched with acetone (2 mL), diluted with CH_2Cl_2, washed with 1 N HCl, aqueous saturated $NaHCO_3$ and H_2O, dried with $MgSO_4$, filtered and concentrated in vacuo. The residue was purified by column chromatography using 1:2 EtOAc-petroleum ether as eluent to give **26** as a colourless oil (180 mg, 84%). R_f 0.34 (1:2 EtOAc/petroleum ether). $[\alpha]_D$ -230º (*c* 1.0, CH_2Cl_2). ^1H NMR (400 MHz, $CDCl_3$) δ 6.97 (d, 2 H, $^3J_{H,H}$ = 9.2 Hz, OPMP), 6.82 (d, 2 H, $^3J_{H,H}$ = 8.8 Hz, OPMP), 5.28 (m, 2 H, H-2, H-3), 5.05 (d, 1 H, $J_{1,2}$ = 7.5 Hz, H-1), 4.07 (m, 2 H, H-4, H-5), 3.84, 3.78 (s, 6 H, COOMe, OPMP), 1.21, 1.18 (s, 18 H, CH_3 (Piv)). ^{13}C NMR (100.6 MHz, $CDCl_3$) δ 178.3, 176.6, 169.0 (CO, Piv, COOMe), 155.7-151.1 (Ar-C), 118.4-114.6 (Ar-CH), 100.8 (C-1), 74.4 (C-5), 73.7 (C-3), 70.4, 70.3 (C-2, C-4), 55.6, 53.0

($COOCH_3$, OPMP), 38.9, 38.8 ($C(CH_3)_3$, Piv), 27.1 (CH_3, Piv). HR MS: *m/z*: calcd. for $C_{24}H_{34}O_{10}Na$: 505.2044; found: 505.2037 $[M + Na]^+$.

Methyl [4-methoxyphenyl 4-*O*-(3,4,6-tri-*O*-acetyl-2-deoxy-2-trifluroacetamido- α,β-D-galactopyranosyl)-2,3-di-*O*-pivaloyl-β-D-glucopyranoside]uronate (27α/β). **22** (37 mg, 0.068 mmol) and **26** (27 mg, 0.056 mmol) were coevaporated with toluene in a flask and dried under vacuum. The starting materials were dissolved in dry CH_2Cl_2 (2 mL) and further dried by stirring over freshly activated 4 Å molecular sieves for 15 min. TMSOTf (84 µL of a 0.16 M solution in CH_2Cl_2, 0.0135 mmol) was added at 0 ºC. After 50 min at 0ºC, the reaction was quenched with Et_3N (1 mL) and filtered, and the solvent was removed under reduced pressure. The residue was purified by column chromatography using 1:4 EtOAc/toluene as eluent to give compounds **27α** (10 mg, 21%) and **27β** (26 mg, 54%) as colourless oils.

27α: R_f 0.35 (1:2 EtOAc/petroleum ether). $[\alpha]_D$ +80º (*c* 0.91, CH_2Cl_2). ^1H NMR (400 MHz, $CDCl_3$) δ 6.93 (m, 2 H, OPMP), 6.84 (m, 2 H, OPMP), 6.35 (d, 1 H, $J_{2,NH}$ = 10.0 Hz, NH), 5.46 (t, 1 H, $J_{2,3}$ = $J_{3,4}$ = 9.0 Hz, H-3A), 5.41 (m, 1 H, H-4B), 5.25 (dd, 1 H, $J_{1,2}$ = 7.1 Hz, H-2A), 5.23 (d, 1 H, $J_{1,2}$ = 3.4 Hz, H-1B), 5.11 (dd, 1 H, $J_{2,3}$ = 11.3 Hz, $J_{3,4}$ = 3.1 Hz, H-3B), 5.07 (d, 1 H, H-1A), 4.59 (td, 1 H, H-2B), 4.55 (t, 1 H, $J_{4,5}$ = 9.0 Hz, H-4A), 4.27 (dd, 1 H, $J_{6a,6b}$ = 11.0 Hz, $J_{5,6a}$= 6.9 Hz, H-6aB), 4.23 (d, 1 H, H-5A), 4.03 (m, 1 H, H-6bB), 3.97 (m, 1 H, H-5B), 3.81, 3.80 (s, 6 H, COOMe, OPMP), 2.20, 2.10, 2.00 (s, 9 H, CH_3CO), 1.18, 1.17 (s, 18 H, CH_3 (Piv)). ^{13}C NMR (100.6 MHz, $CDCl_3$) δ 176.8-167.9 (CO, Ac, Piv, COOMe), 157.7 ($COCF_3$), 155.7-150.6 (Ar-C), 118.1-114.6 (Ar-CH), 115.5 ($COCF_3$), 100.2 (C-1A), 96.7 (C-1B), 73.9, 73.8 (C-3A, C-5A), 72.7 (C-4A), 71.1 (C-2A), 67.8, 67.6 (C-3B, C-5B), 66.7 (C-4B), 60.9 (C-6B), 55.6, 53.1 ($COOCH_3$, OPMP), 47.7 (C-2B), 38.9 ($C(CH_3)_3$, Piv), 27.1, 27.0 (CH_3, Piv), 20.7, 20.4 (CH_3, Ac). HR MS: *m/z*: calcd. for $C_{38}H_{50}O_{18}NF_3Na$: 888.2872; found: 888.2864 $[M + Na]^+$.

27β: R_f 0.23 (1:2 EtOAc/petroleum ether). $[\alpha]_D$ -139º (*c* 0.8, CH_2Cl_2). ^1H NMR (400 MHz, $CDCl_3$) δ 6.94 (d, 2 H, $^3J_{H,H}$ = 9.1 Hz, OPMP), 6.83 (d, 2 H, $^3J_{H,H}$ = 9.3 Hz, OPMP), 6.75 (d, 1 H, $J_{2,NH}$ = 8.7 Hz, NH), 5.36 (m, 1 H, H-4B), 5.32 (m, 1 H, H-3A), 5.22 (dd, 1 H, $J_{2,3}$ = 9.3 Hz, $J_{1,2}$ = 7.3 Hz, H-2A), 5.17 (dd, 1 H, $J_{2,3}$ = 11.1 Hz, $J_{3,4}$ = 3.3 Hz, H-3B), 5.03 (d, 1 H, H-1A), 4.84 (d, 1 H, $J_{1,2}$ = 8.4 Hz, H-1B), 4.31 (t, 1 H, $J_{3,4}$ = $J_{4,5}$ = 9.4 Hz, H-4A), 4.14 (m, 3 H, H-5A, H-6aB, H-6bB), 4.00 (m, 1 H, H-2B), 3.95 (m, 1 H, H-5B), 3.82, 3.79 (s, 6 H, COOMe, OPMP), 2.12, 2.10, 2.00 (s, 9 H, CH_3CO), 1.23, 1.19 (s, 18 H, CH_3 (Piv)). ^{13}C NMR (100.6 MHz, $CDCl_3$) δ 177.1, 176.7, 170.4, 170.3, 169.9, 169.3 (CO, Ac, Piv, COOMe), 157.7 ($COCF_3$), 155.8-150.8 (Ar-C), 118.2-114.6 (Ar-CH), 115.5 ($COCF_3$), 100.3 (C-1A), 99.2 (C-1B), 74.5, 74.0 (C-4A, C-5A), 71.4 (C-5B), 71.1, 70.0 (C-2A, C-3A, C-3B), 66.3 (C-4B), 61.0 (C-6B), 55.6, 53.4 ($COOCH_3$, OPMP), 51.9 (C-2B), 38.8 ($C(CH_3)_3$, Piv), 27.1 (CH_3, Piv), 20.7, 20.5, 20.4 (CH_3, Ac). HR MS: *m/z*: calcd. for $C_{38}H_{50}O_{18}NF_3Na$: 888.2872; found: 888.2863 $[M + Na]^+$.

Methyl [4-methoxyphenyl 4-*O*-(3,4,6-tri-*O*-acetyl-2-deoxy-2-trifluroacetamido-β-D-galactopyranosyl)-2,3-di-*O*-benzoyl-β-D-glucopyranoside]uronate (29β): 22 (45 mg, 0.082 mmol) and **28** (33 mg, 0.063 mmol) were coevaporated with toluene in a flask and dried under vacuum. The starting materials were dissolved in dry CH_2Cl_2 (1.5 mL) and further dried by stirring over freshly activated 4 Å molecular sieves for 15 min. TMSOTf (100 µL of a 0.16 M solution in CH_2Cl_2, 0.016 mmol) was added at 0 ºC. After 50 min at 0 ºC, the reaction was quenched with Et_3N (0.3 mL) and filtered, and the solvent was removed under reduced pressure. The residue was purified by column chromatography using 2:3 EtOAc/petroleum ether as eluent to give compound **29β** (40 mg, 70%) as white amorphous solid. R_f 0.14 (2:3 EtOAc/petroleum ether). $[\alpha]_D$ +11º (*c* 1.0, CH_2Cl_2). ^{1}H NMR (400 MHz, $CDCl_3$) δ 8.03-7.39 (m, 10H, Ar), 6.96 (d, 2 H, $^3J_{H,H}$ = 9.0 Hz, OPMP), 6.81 (d, 2 H, $^3J_{H,H}$ = 9.0 Hz, OPMP), 6.63 (d, 1 H, $J_{2,NH}$ = 9.0 Hz, NH), 5.76 (t, 1 H, $J_{2,3} = J_{3,4}$ = 9.4 Hz, H-3A), 5.65 (dd, 1 H, $J_{1,2}$ = 7.1 Hz, H-2A), 5.26 (d, 1 H, H-1A), 5.18 (d, 1 H, $J_{3,4}$ = 3.1 Hz, H-4B), 5.05 (dd, 1 H, $J_{2,3}$ = 11.3 Hz, H-3B), 4.88 (d, 1 H, $J_{1,2}$ = 8.4 Hz, H-1B), 4.41 (t, 1 H, $J_{4,5}$ = 9.4 Hz, H-4A), 4.28 (d, 1H, H-5A), 4.11 (m, 1 H, H-2B), 3.79, 3.78 (s, 6 H, COOMe, OPMP), 3.73 (t, 1H, H-5B), 3.29 (m, 2H, H-6aB, H-6bB), 2.02, 2.00, 1.97 (s, 9 H, CH_3CO). ^{13}C NMR (100.6 MHz, $CDCl_3$) δ 170.4, 170.01, 169.97, 169.2, 165.27, 165.20, (CO, Ac, Bz, COOMe), 157.2 (*C*OCF_3), 155.8-114.2 (Ar), 115.7 (CO*C*F_3), 100.9 (C-1A), 100.6 (C-1B), 77.0 (C-4A), 73.7 (C-5A), 72.1, 71.6 (C-2A, C-3A), 70.8 (C-5B), 70.2 (C-3B), 65.9 (C-4B), 60.1 (C-6B), 55.6, 53.2 (COO*C*H_3, OPMP), 51.5 (C-2B), 20.6, 20.5, 20.4 (CH_3, Ac). HR MS: *m/z*: calcd. for $C_{42}H_{42}O_{18}NF_3Na$: 928.2246; found: 928.2236 $[M + Na]^+$.

References

1. Macchione, G.; de Paz, J. L.; Nieto, P. M. *Carbohydr. Res.* **2014,** *394*, 17-25.

2. Maza, S.; Gandia-Aguado, N.; de Paz, J. L.; Nieto, P. M. *Bioorg. Med. Chem.* **2018,** *26*, 1076-1085.

3. Maza, S.; Mar Kayser, M.; Macchione, G.; Lopez-Prados, J.; Angulo, J.; de Paz, J. L.; Nieto, P. M. *Org. Biomol. Chem.* **2013,** *11*, 3510-3525.

4. Macchione, G.; Maza, S.; Kayser, M. M.; de Paz, J. L.; Nieto, P. M. *Eur. J. Org. Chem.* **2014,** 3868-3884.

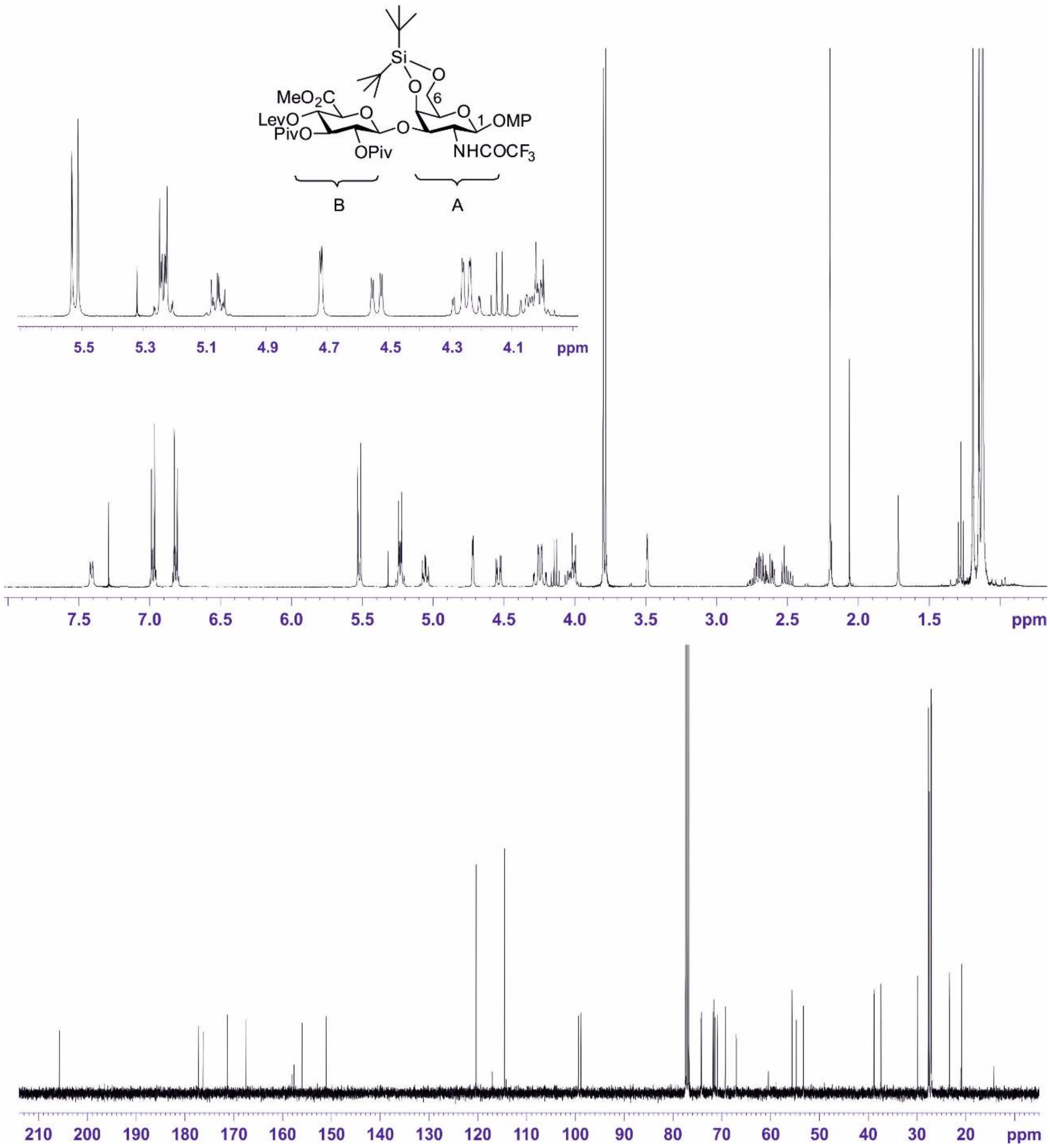

^{1}H and ^{13}C NMR (400 MHz, 100.6 MHz, $CDCl_3$) spectra of **5**.

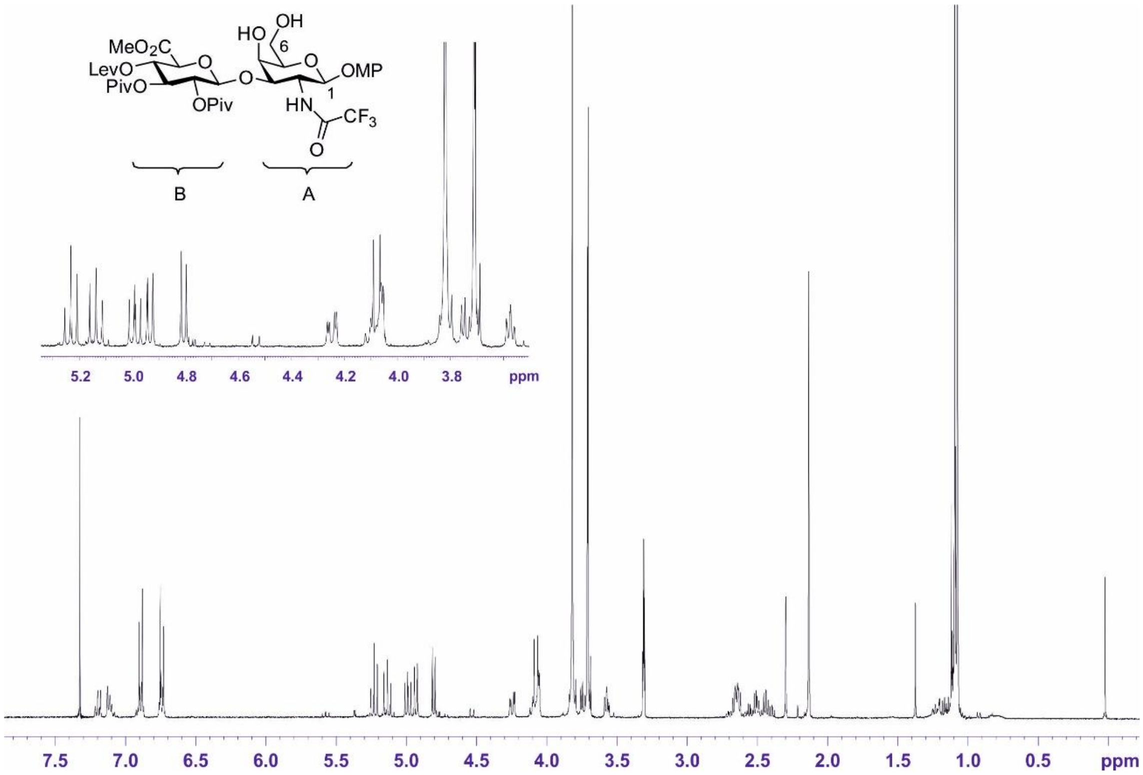

^1H NMR (400 MHz, 5:1 $CDCl_3$-MeOD) spectrum of **6**.

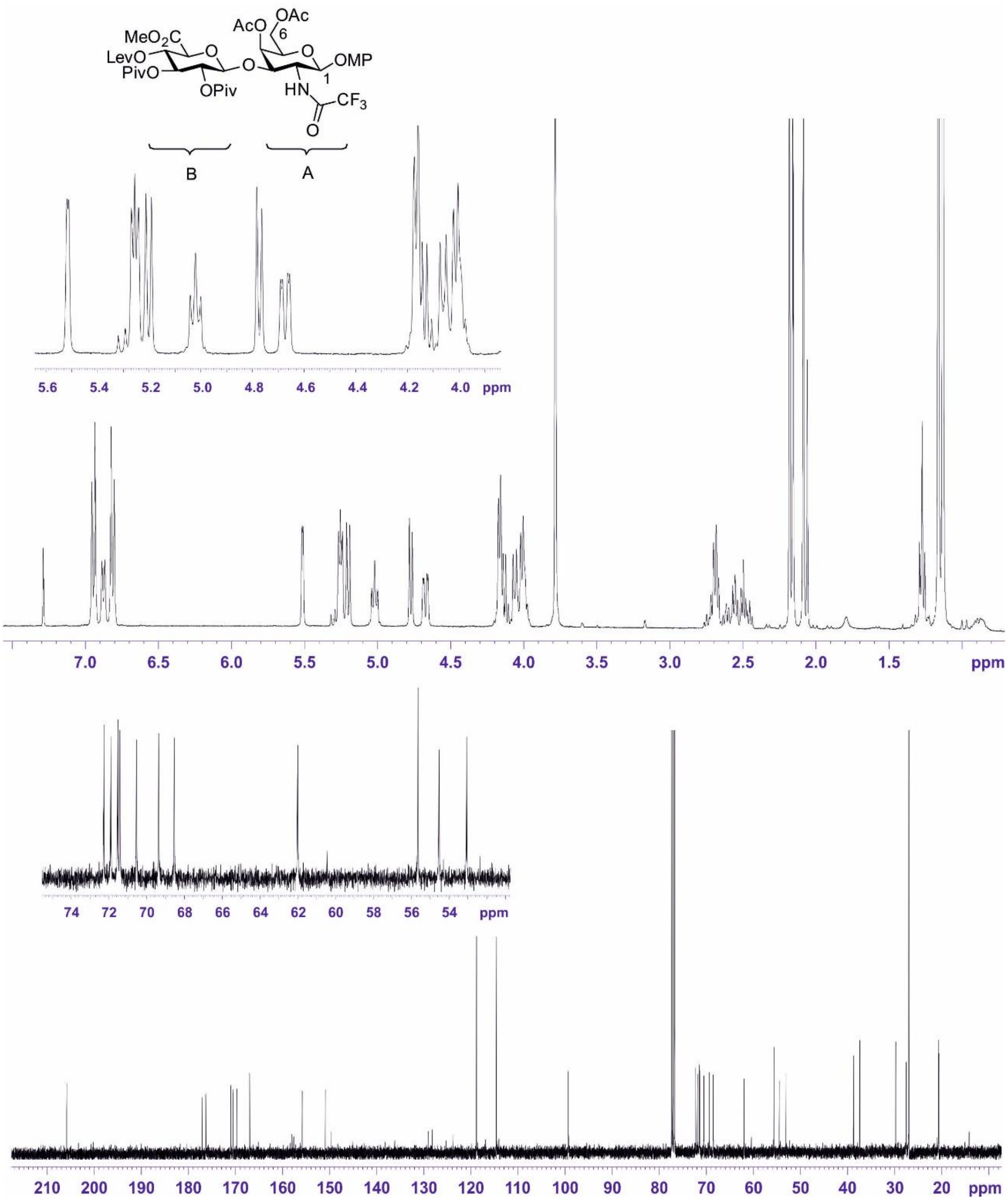

^{1}H and ^{13}C NMR (400 MHz, 100.6 MHz, $CDCl_3$) spectra of **7**.

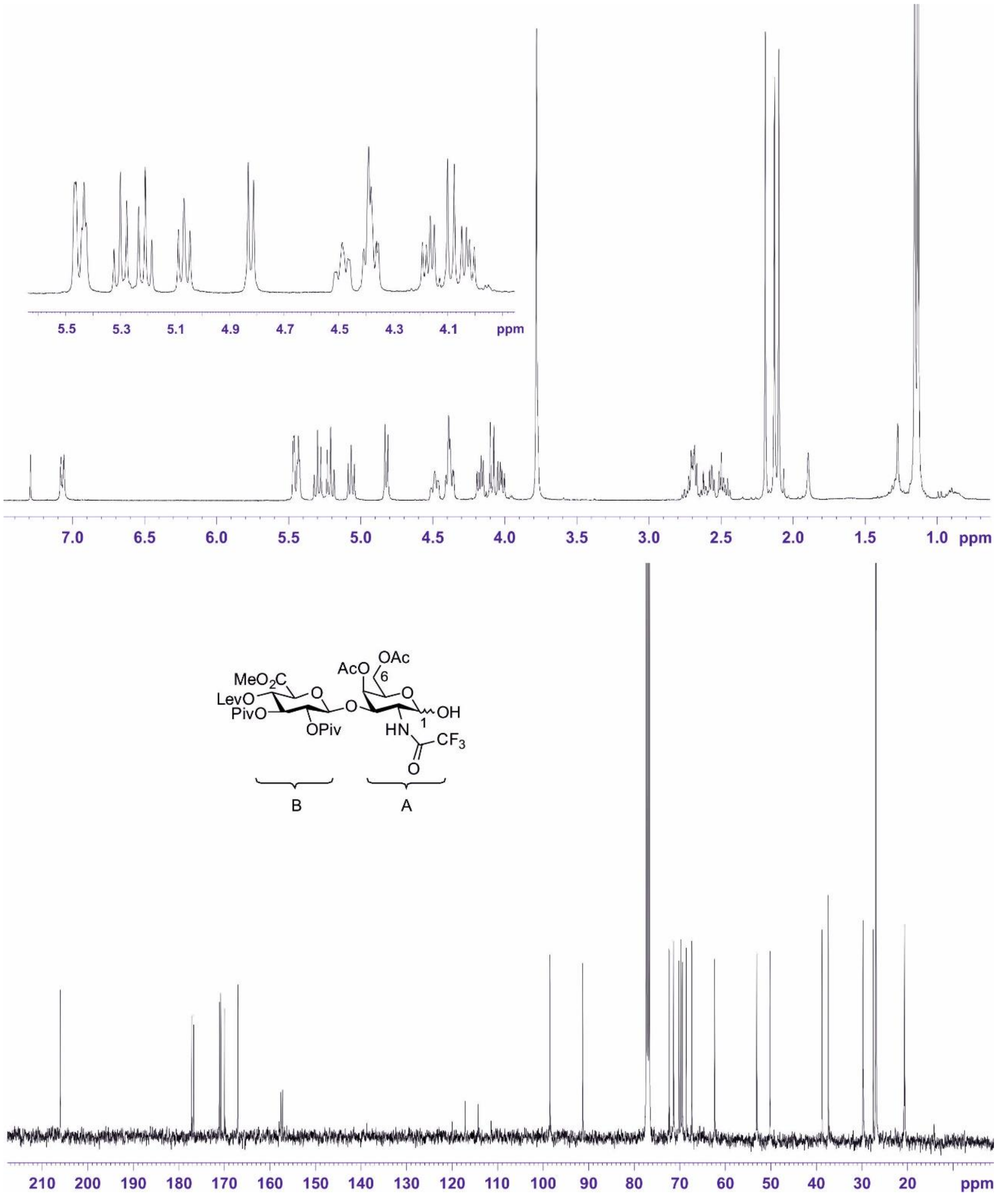

^{1}H and ^{13}C NMR (400 MHz, 100.6 MHz, $CDCl_3$) spectra of **8**.

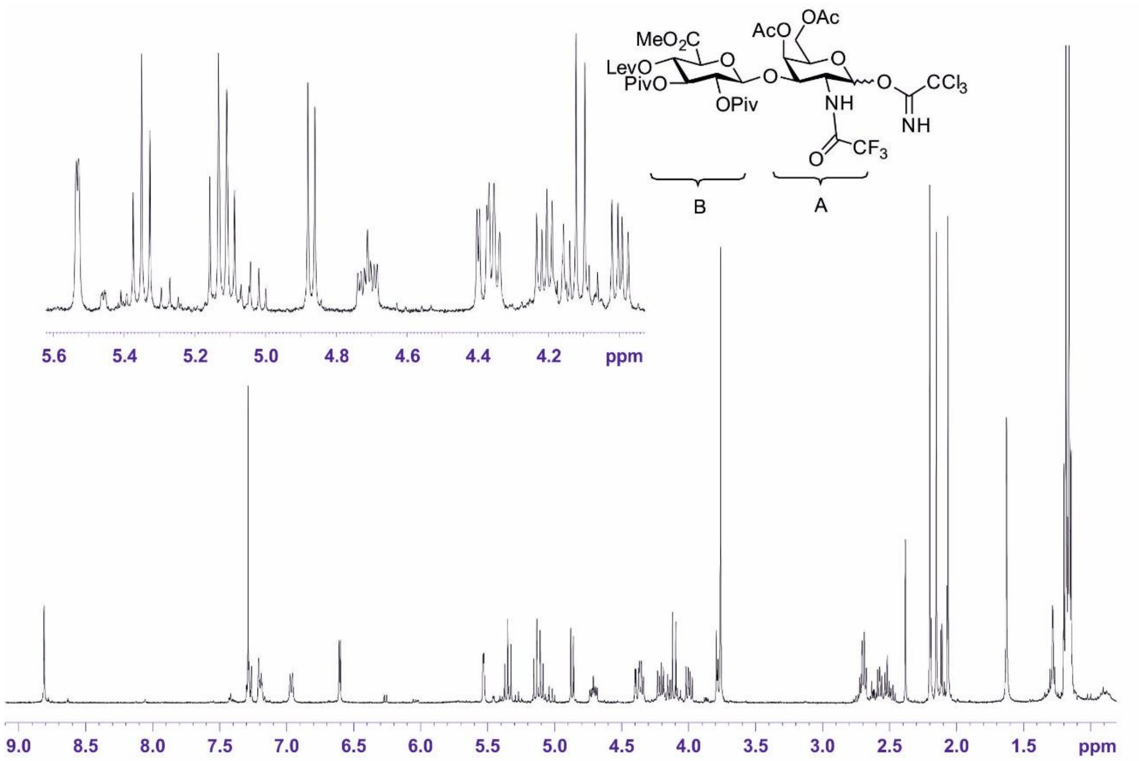

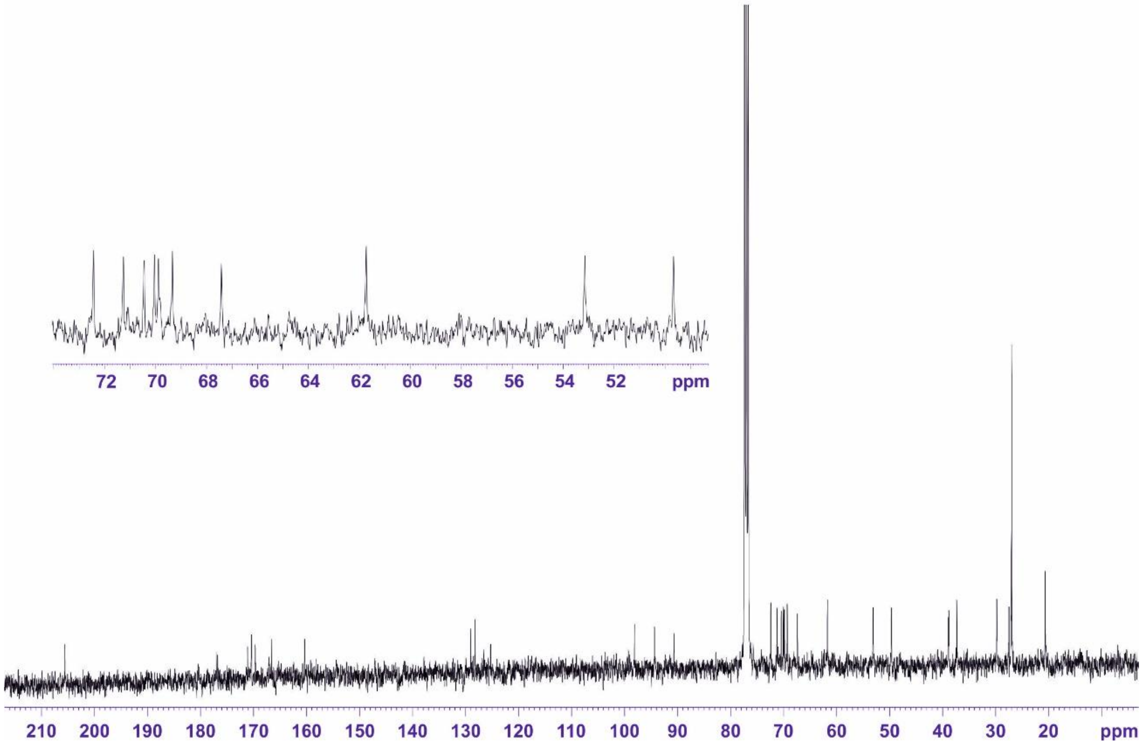

^{1}H and ^{13}C NMR (400 MHz, 100.6 MHz, $CDCl_3$) spectra of **1**.

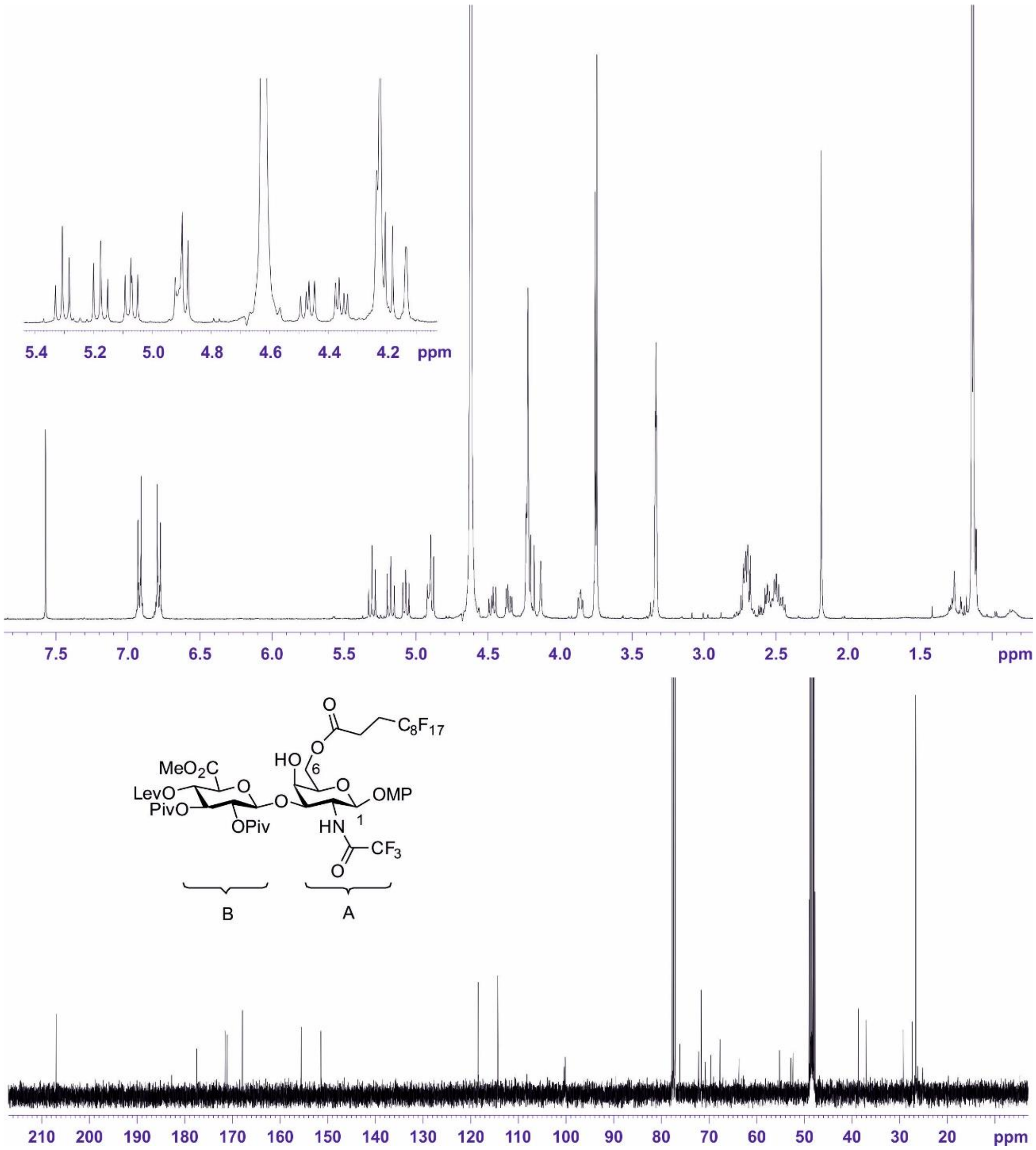

^{1}H and ^{13}C NMR (400 MHz, 100.6 MHz, 1:1 $CDCl_3$-MeOD) spectra of **9**.

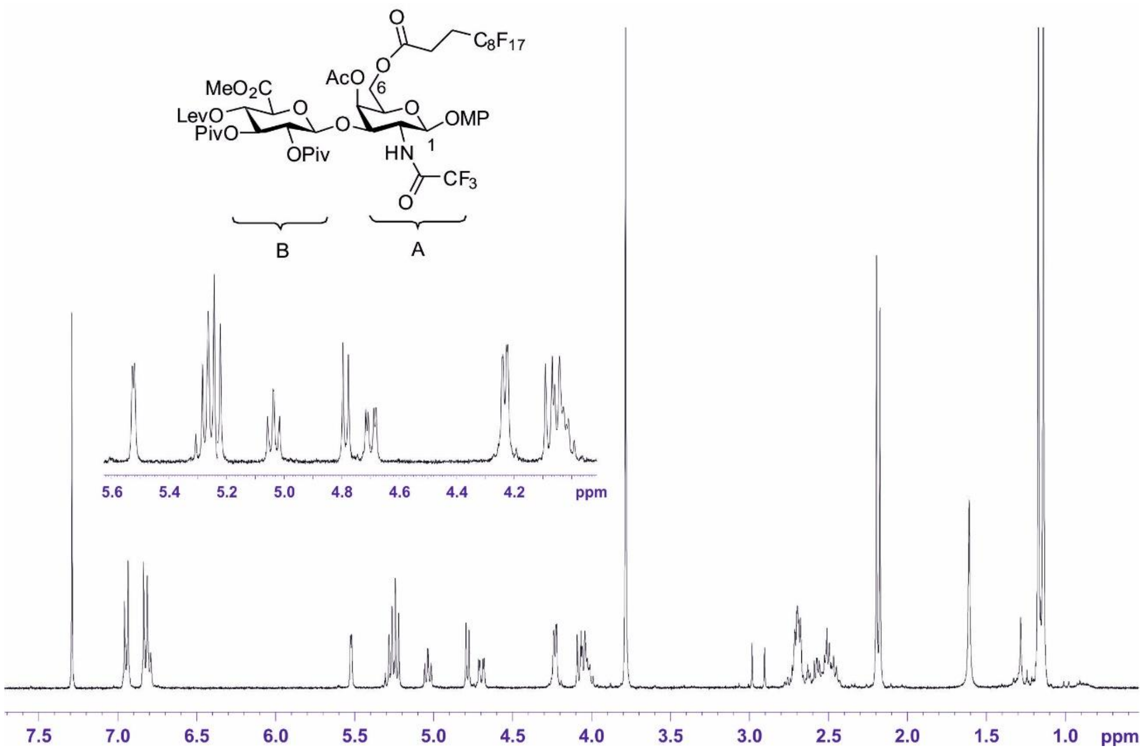

^{1}H NMR (400 MHz, $CDCl_3$) spectrum of **10**.

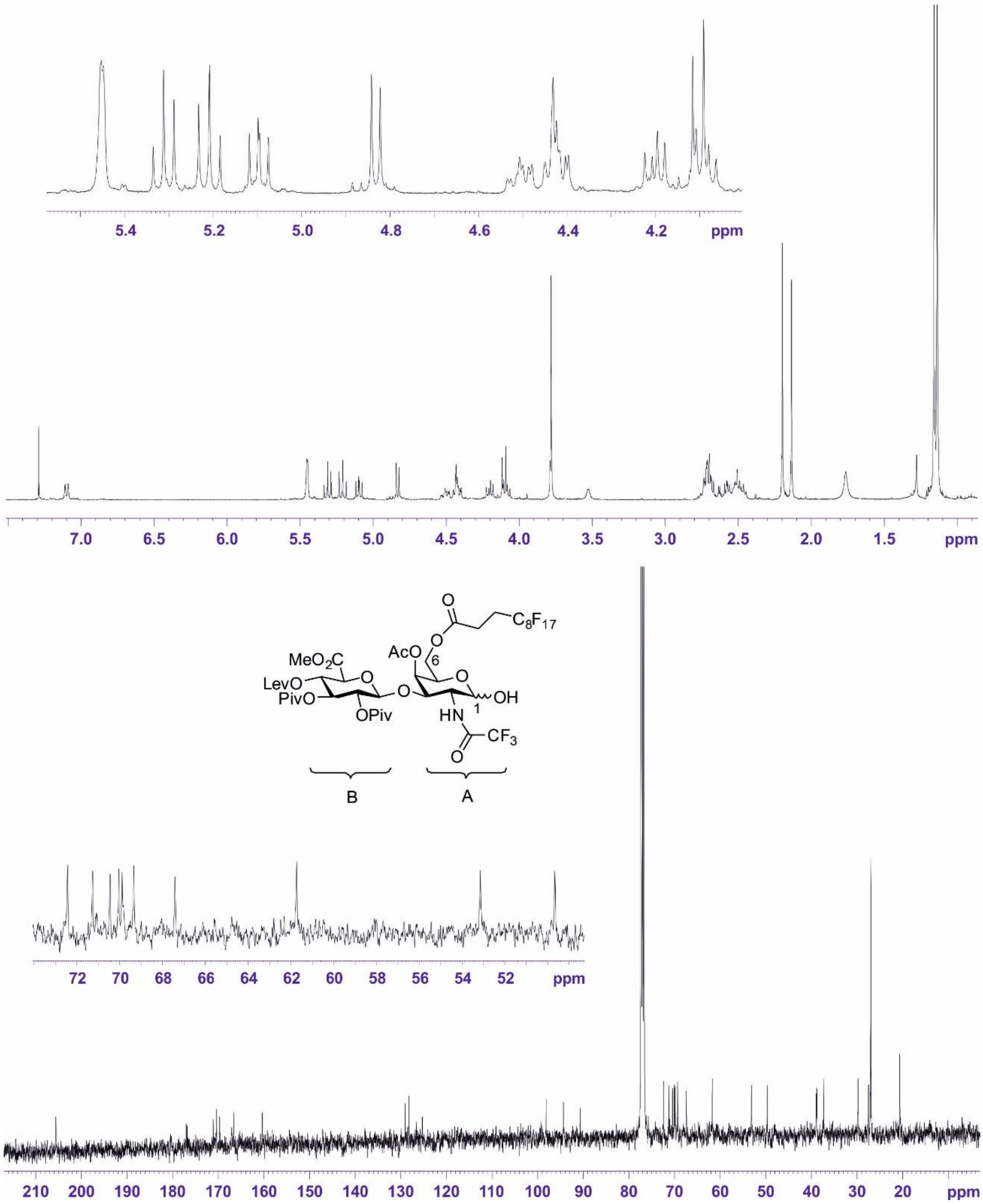

^{1}H and ^{13}C NMR (400 MHz, 100.6 MHz, $CDCl_3$) spectra of **11**.

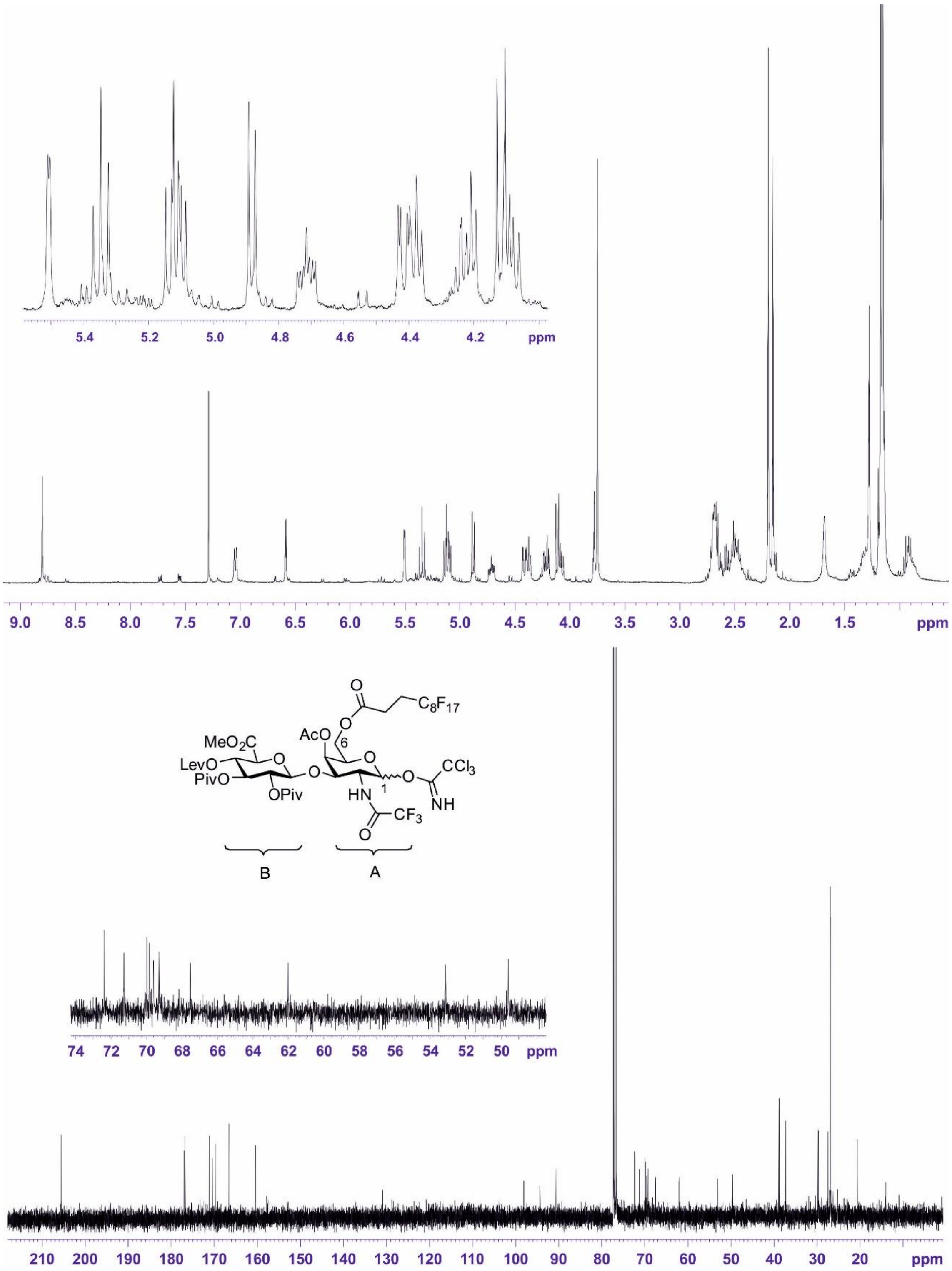

^{1}H and ^{13}C NMR (400 MHz, 100.6 MHz, $CDCl_3$) spectra of **12**.

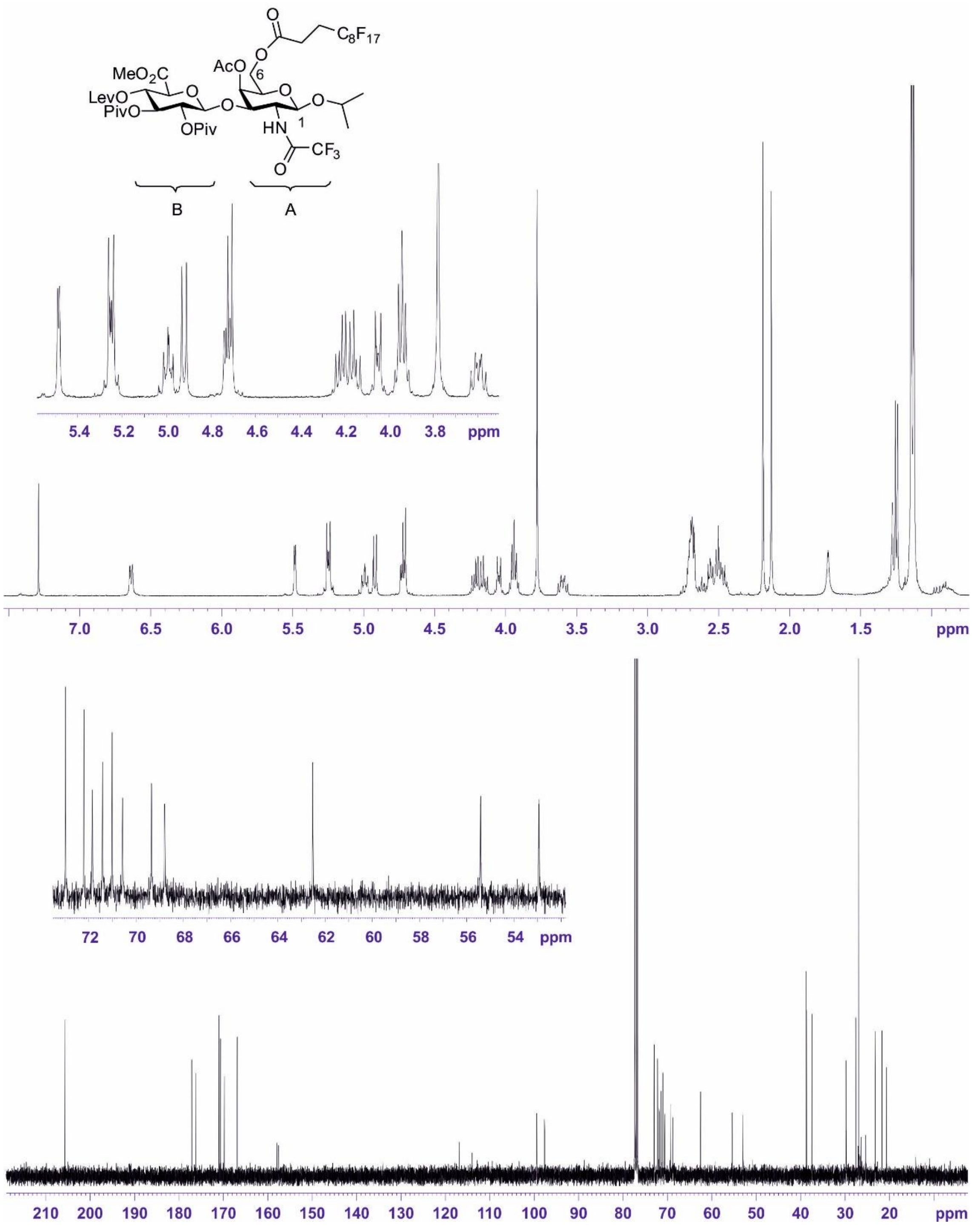

^{1}H and ^{13}C NMR (400 MHz, 100.6 MHz, $CDCl_3$) spectra of **13**.

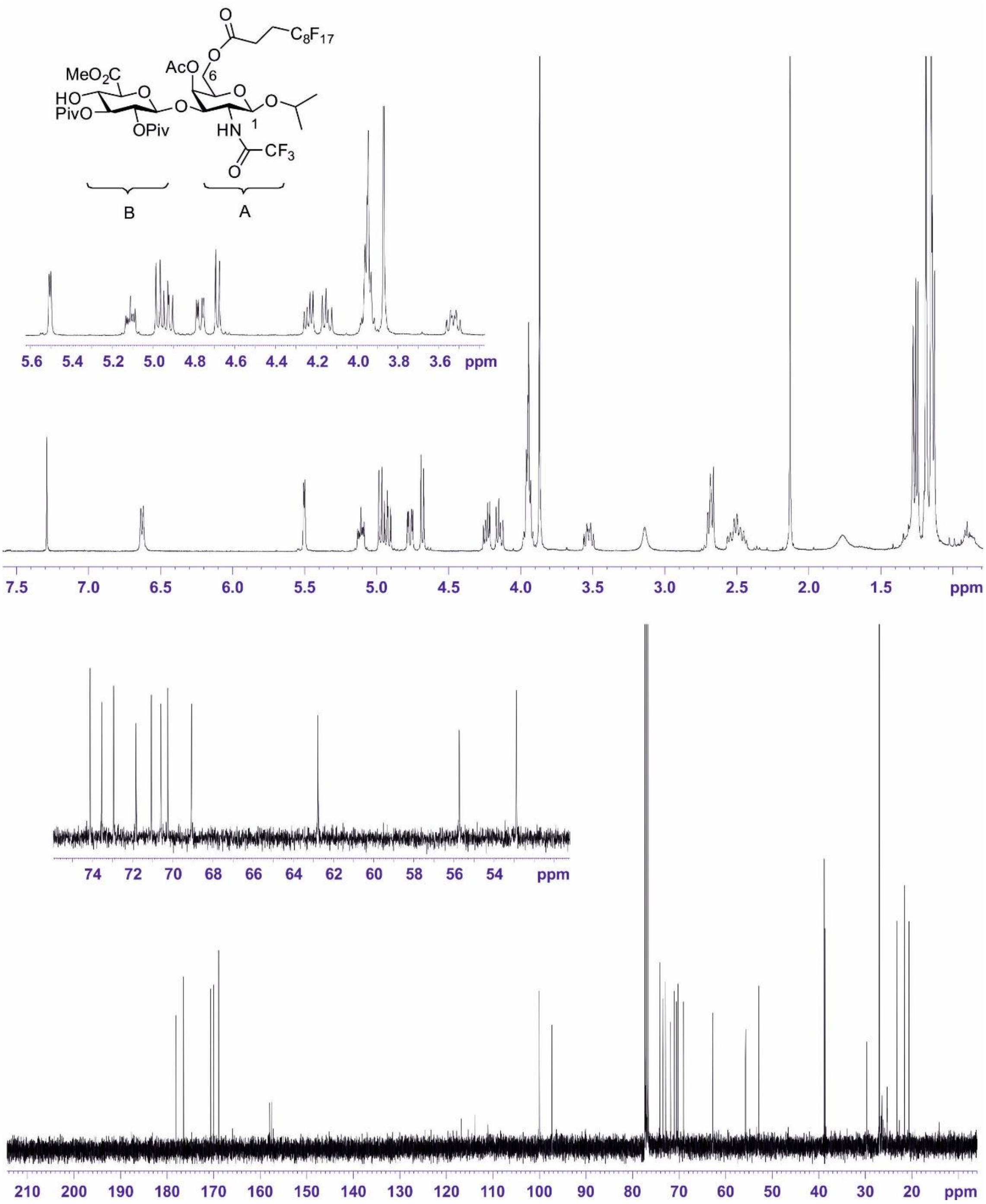

^{1}H and ^{13}C NMR (400 MHz, 100.6 MHz, $CDCl_3$) spectra of **2**.

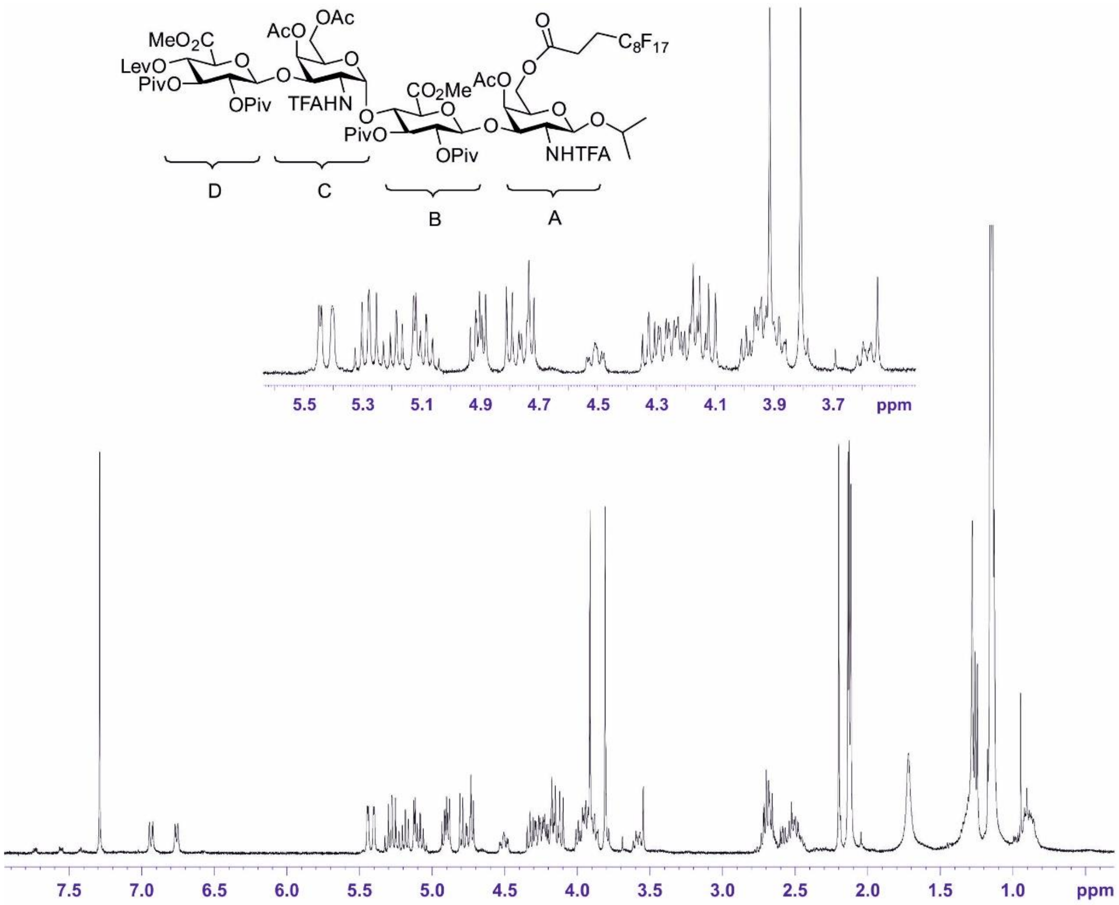

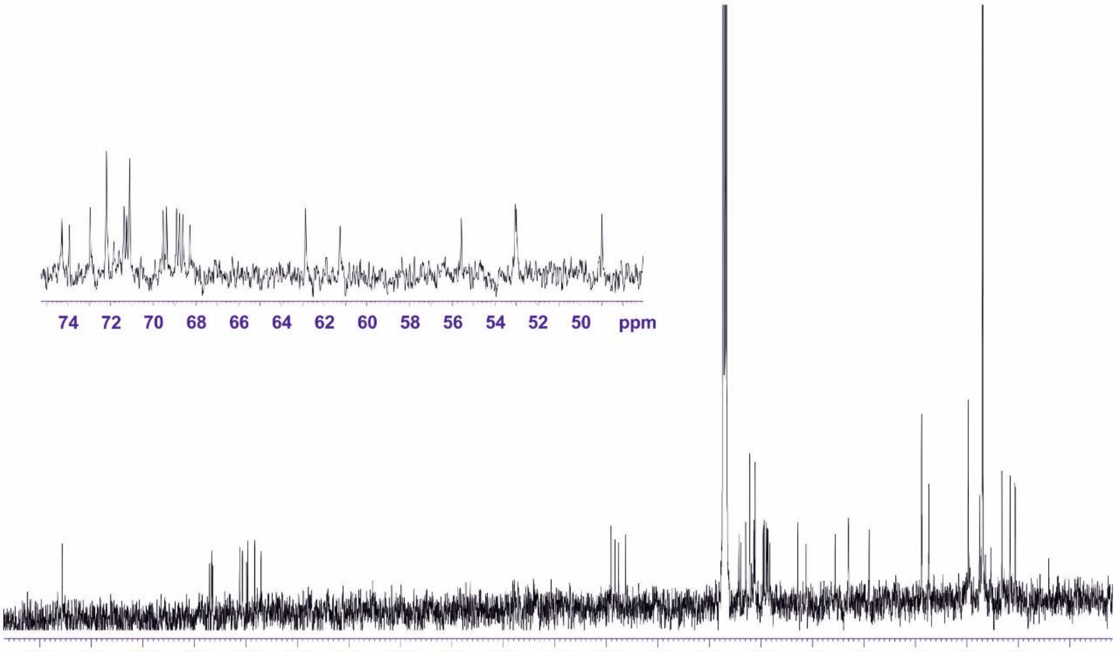

^{1}H and ^{13}C NMR (400 MHz, 100.6 MHz, $CDCl_3$) spectra of **14α**.

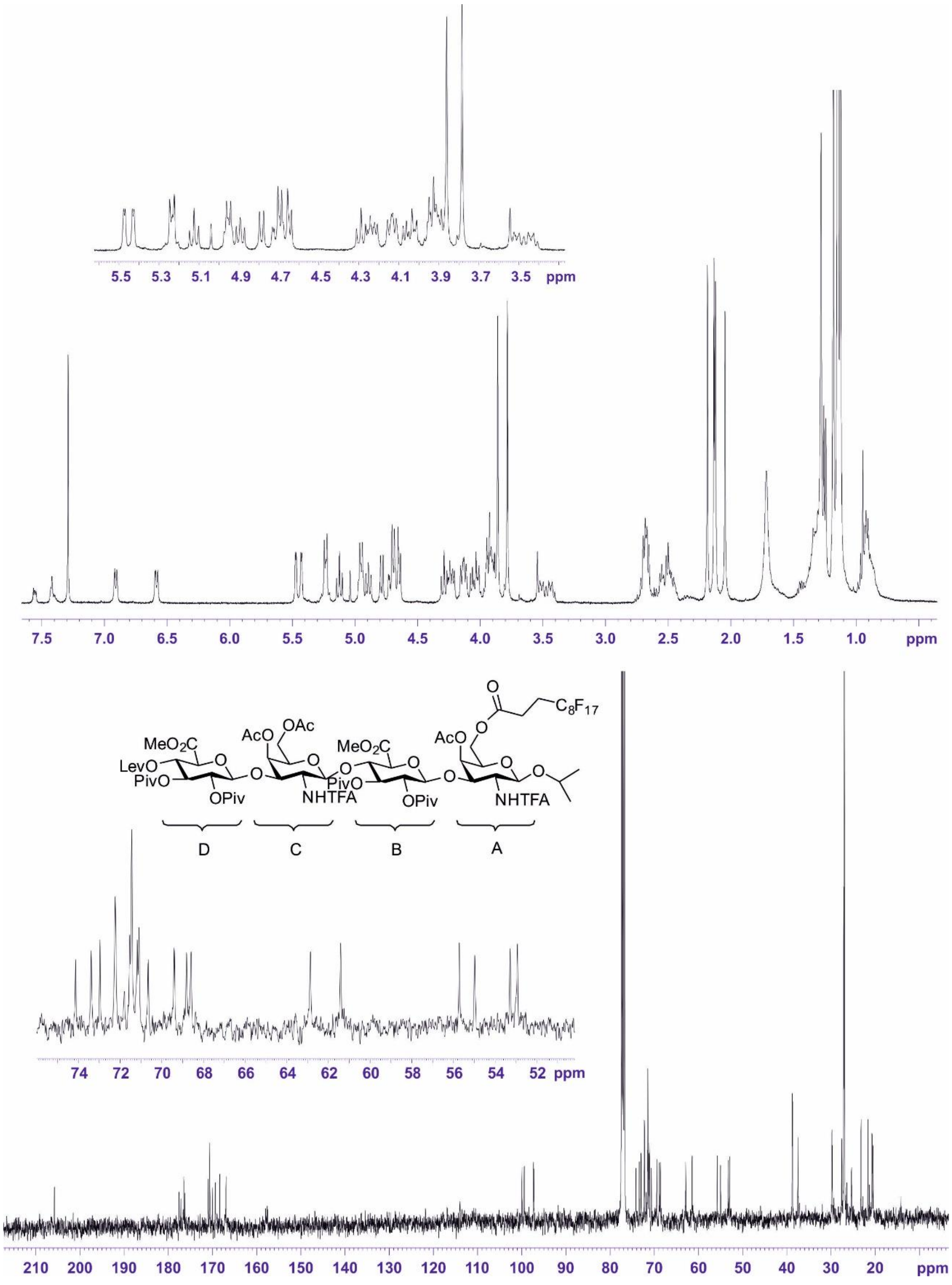

^{1}H and ^{13}C NMR (400 MHz, 100.6 MHz, $CDCl_3$) spectra of **14β**.

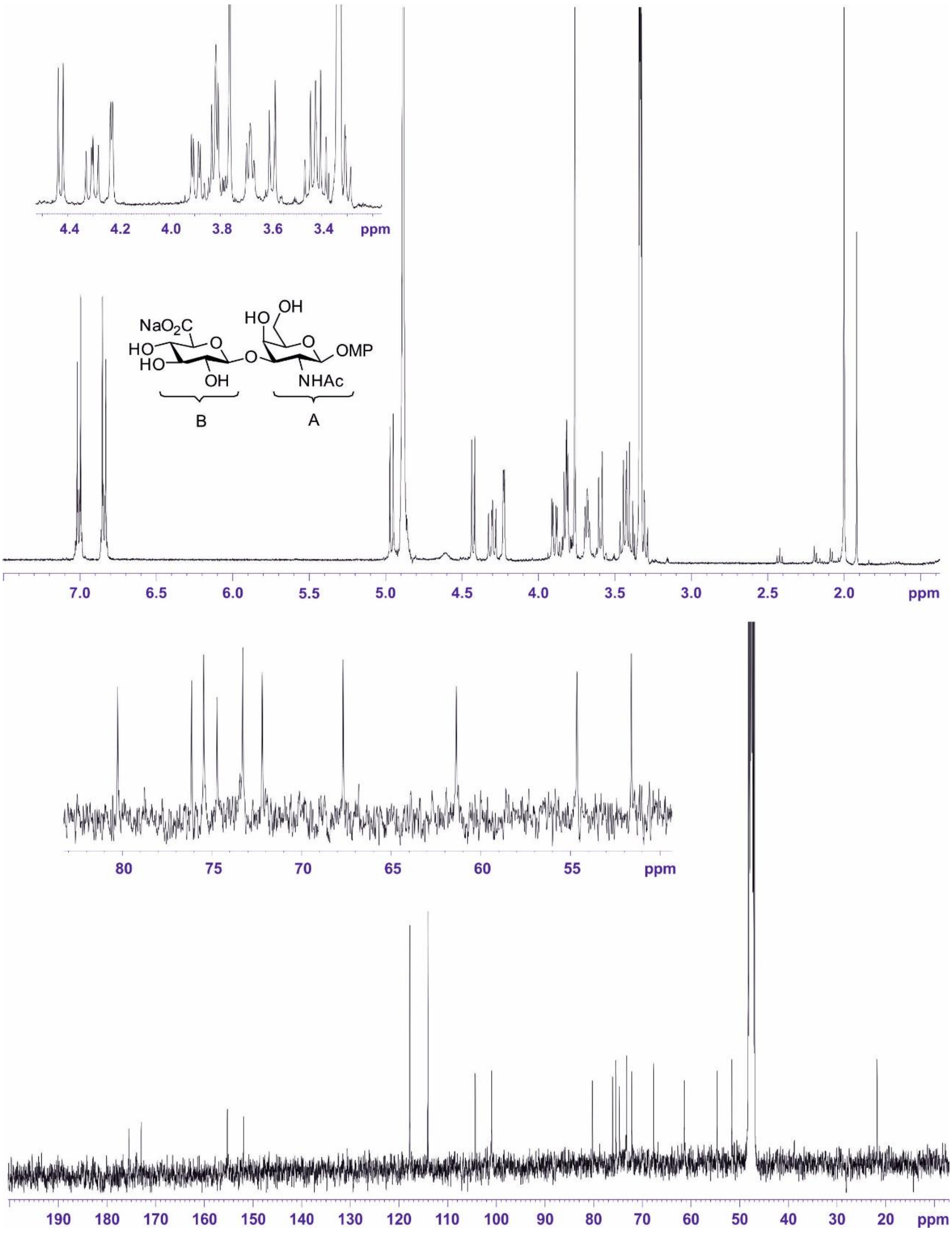

^{1}H and ^{13}C NMR (400 MHz, 100.6 MHz, MeOD) spectra of **15**.

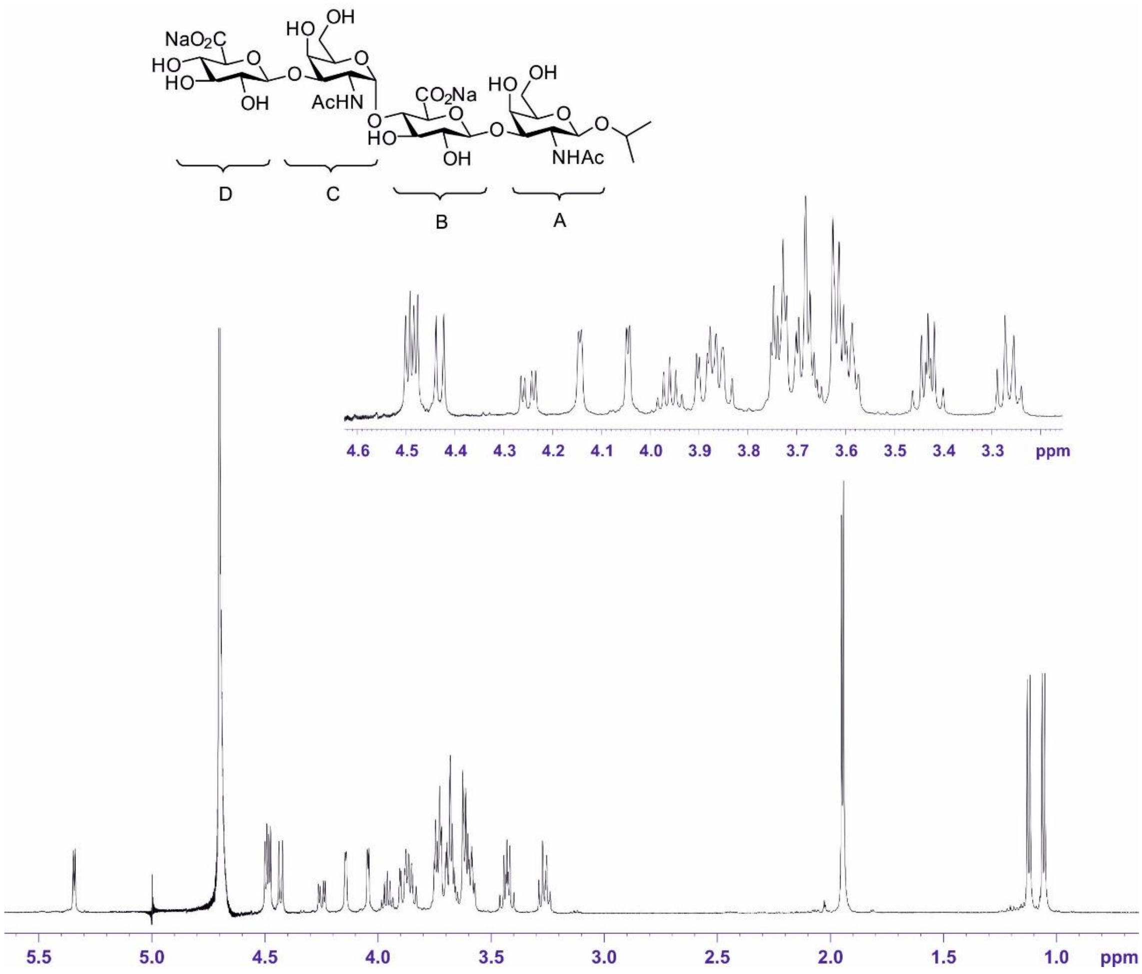

^{1}H NMR (500 MHz, D_2O) spectrum of **16α**.

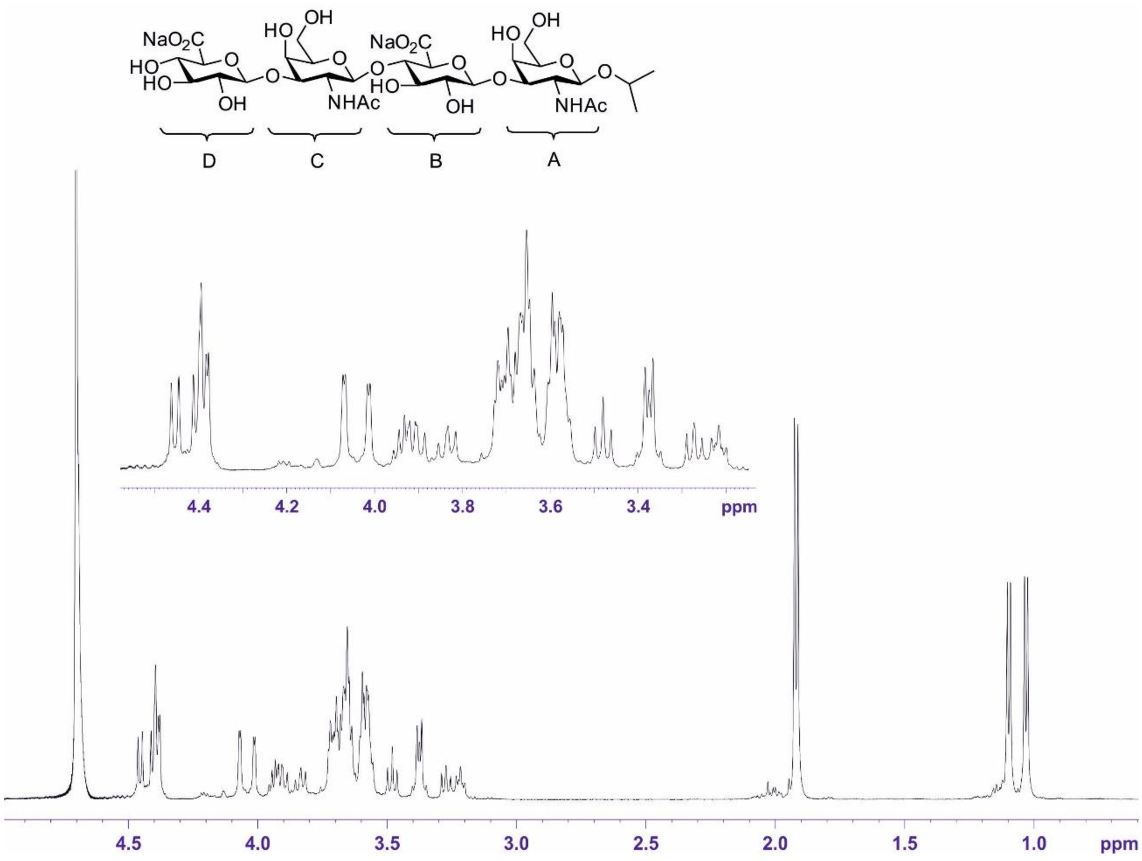

^{1}H NMR (500 MHz, D_2O) spectrum of **16β**.

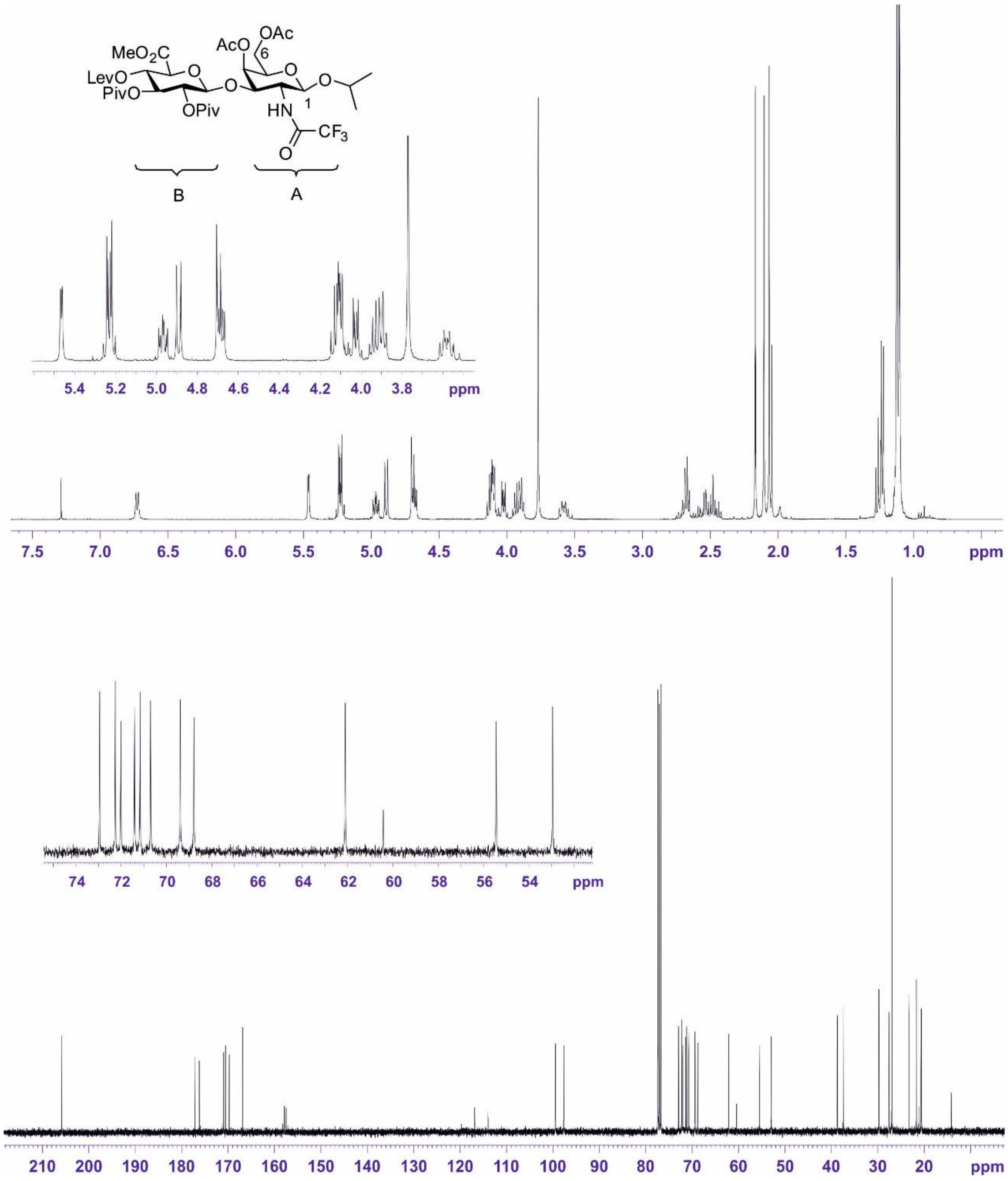

^{1}H and ^{13}C NMR (400 MHz, 100.6 MHz, $CDCl_3$) spectra of **17**.

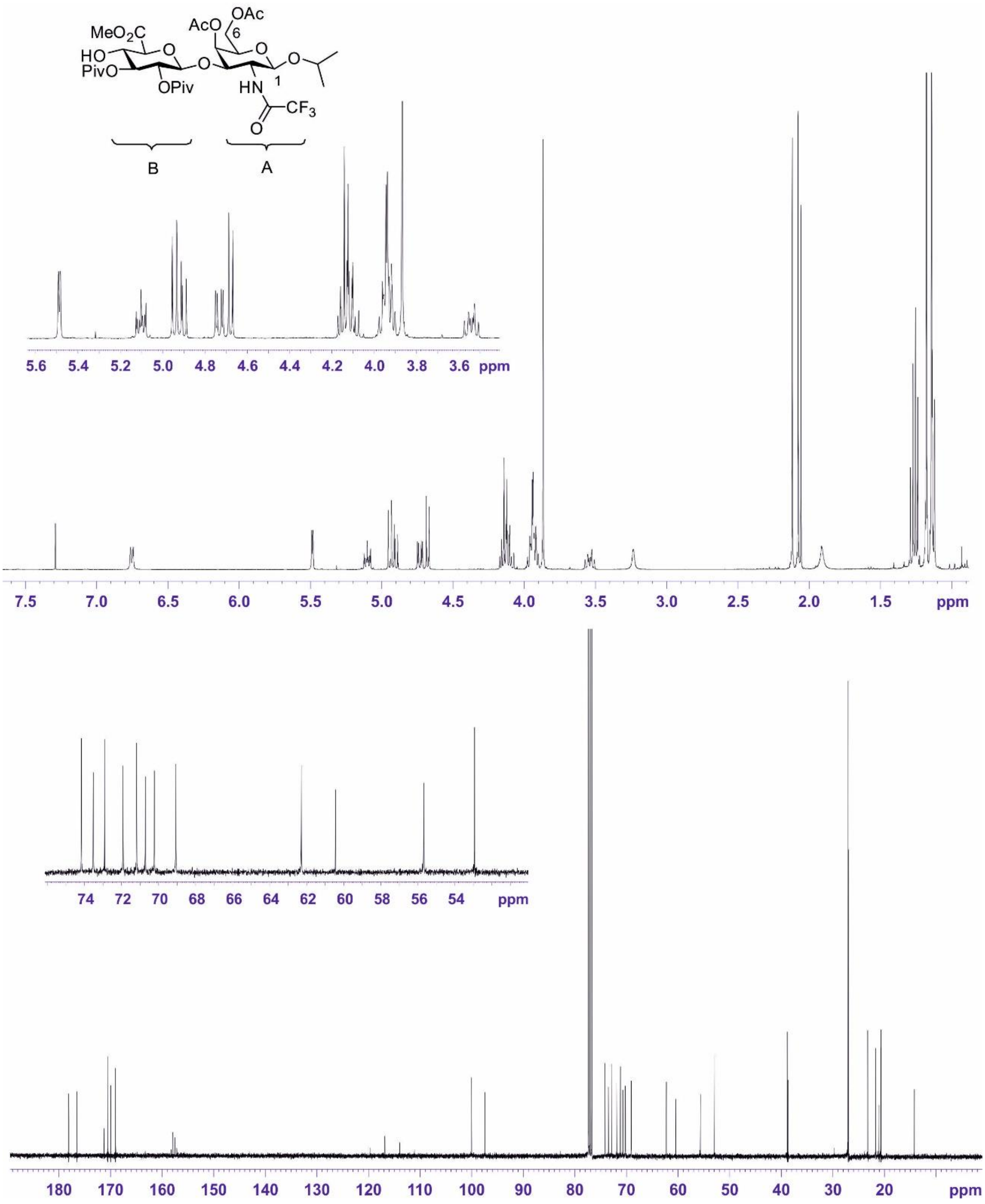

^{1}H and ^{13}C NMR (400 MHz, 100.6 MHz, $CDCl_3$) spectra of **18**.

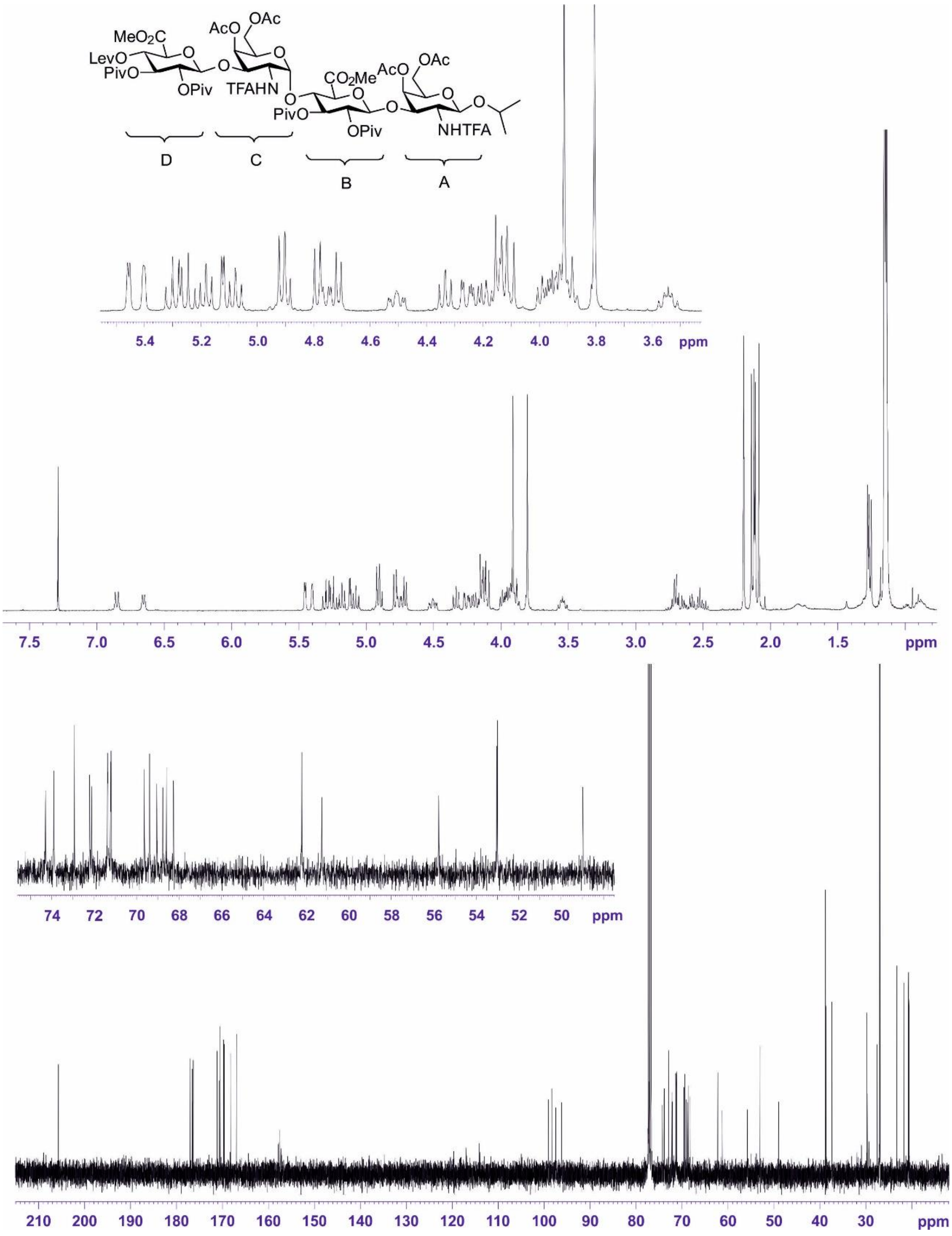

^{1}H and ^{13}C NMR (400 MHz, 100.6 MHz, $CDCl_3$) spectra of **19α**.

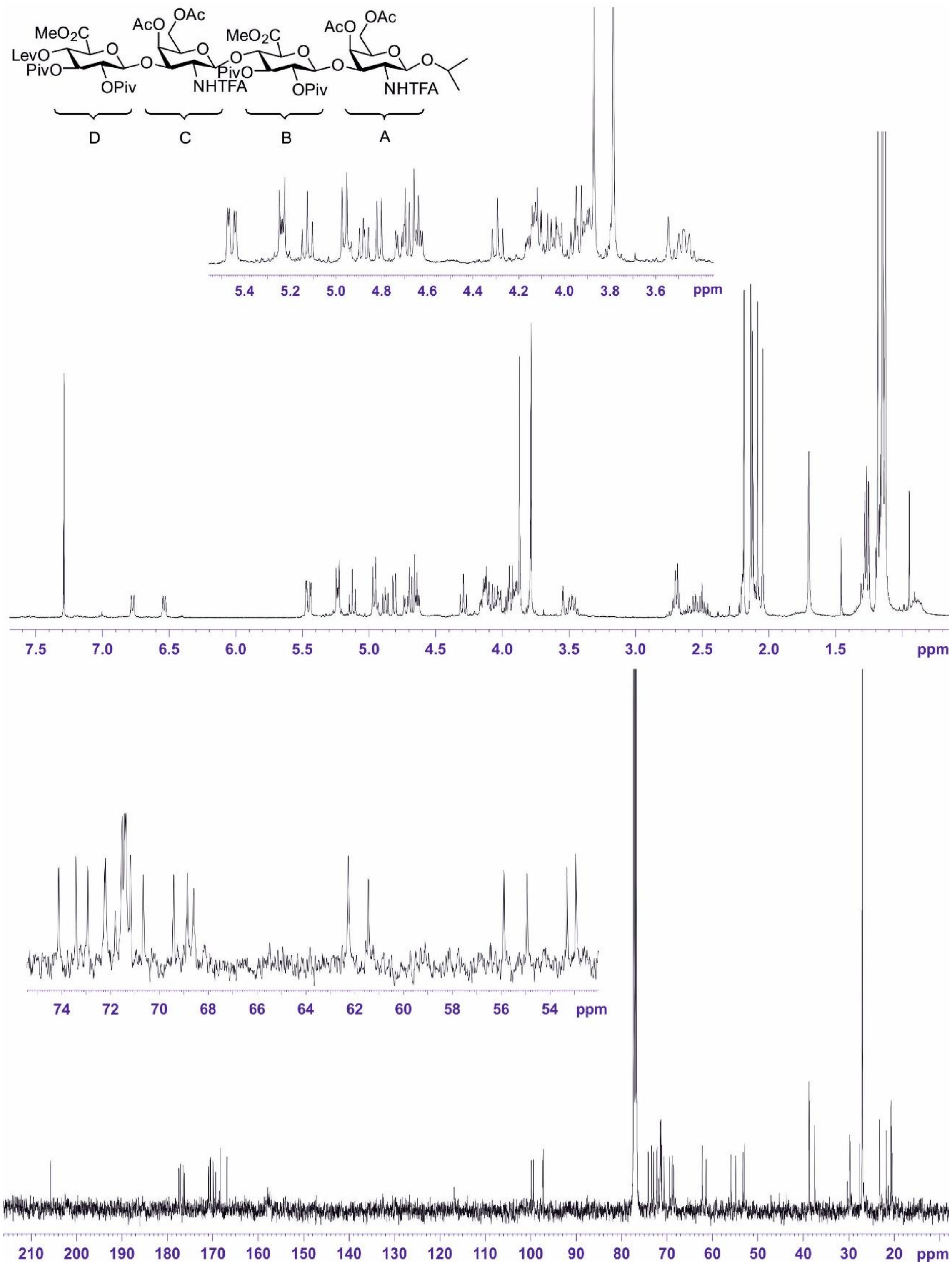

^{1}H and ^{13}C NMR (400 MHz, 100.6 MHz, $CDCl_3$) spectra of **19β**.

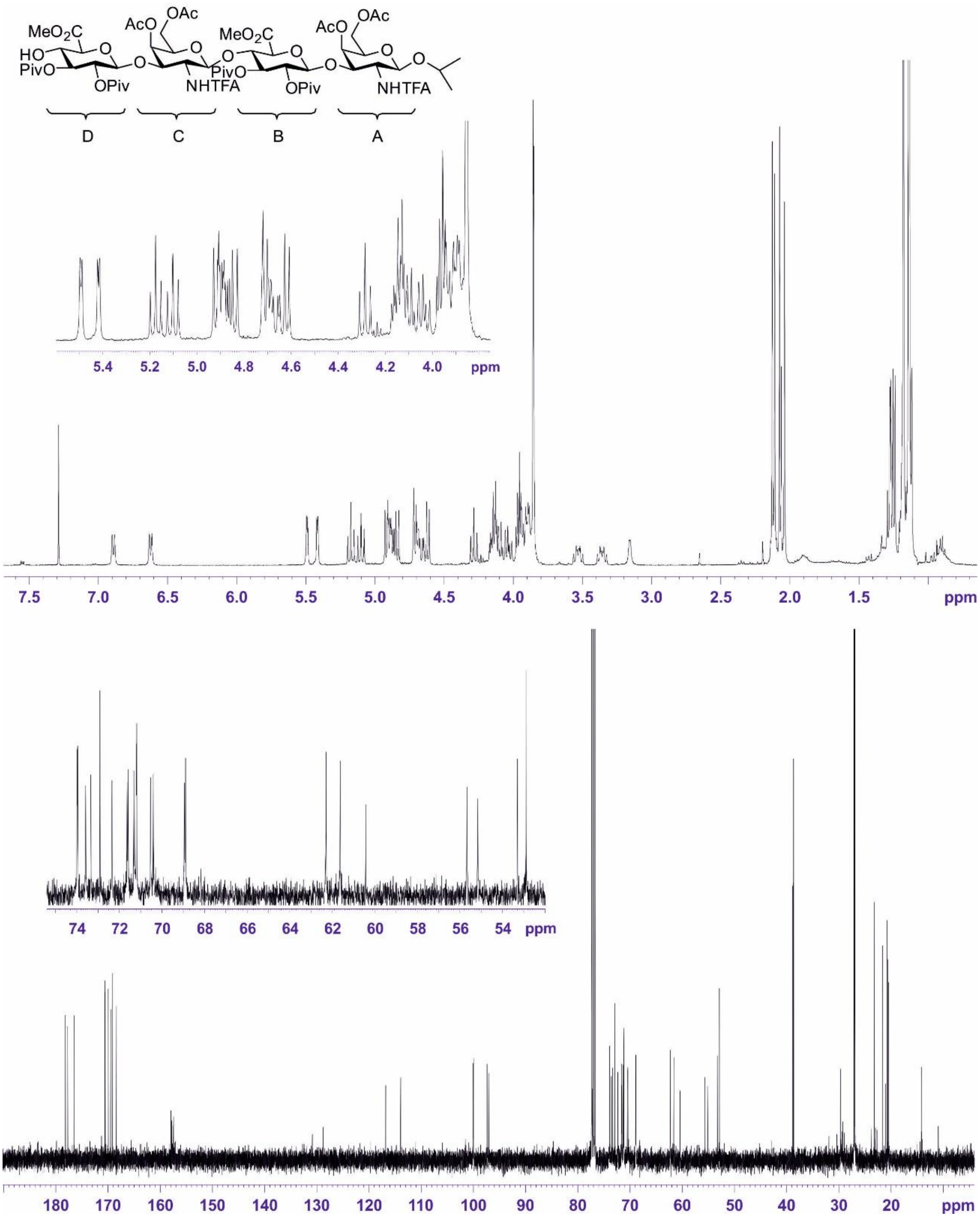

^{1}H and ^{13}C NMR (400 MHz, 100.6 MHz, $CDCl_3$) spectra of **20**.

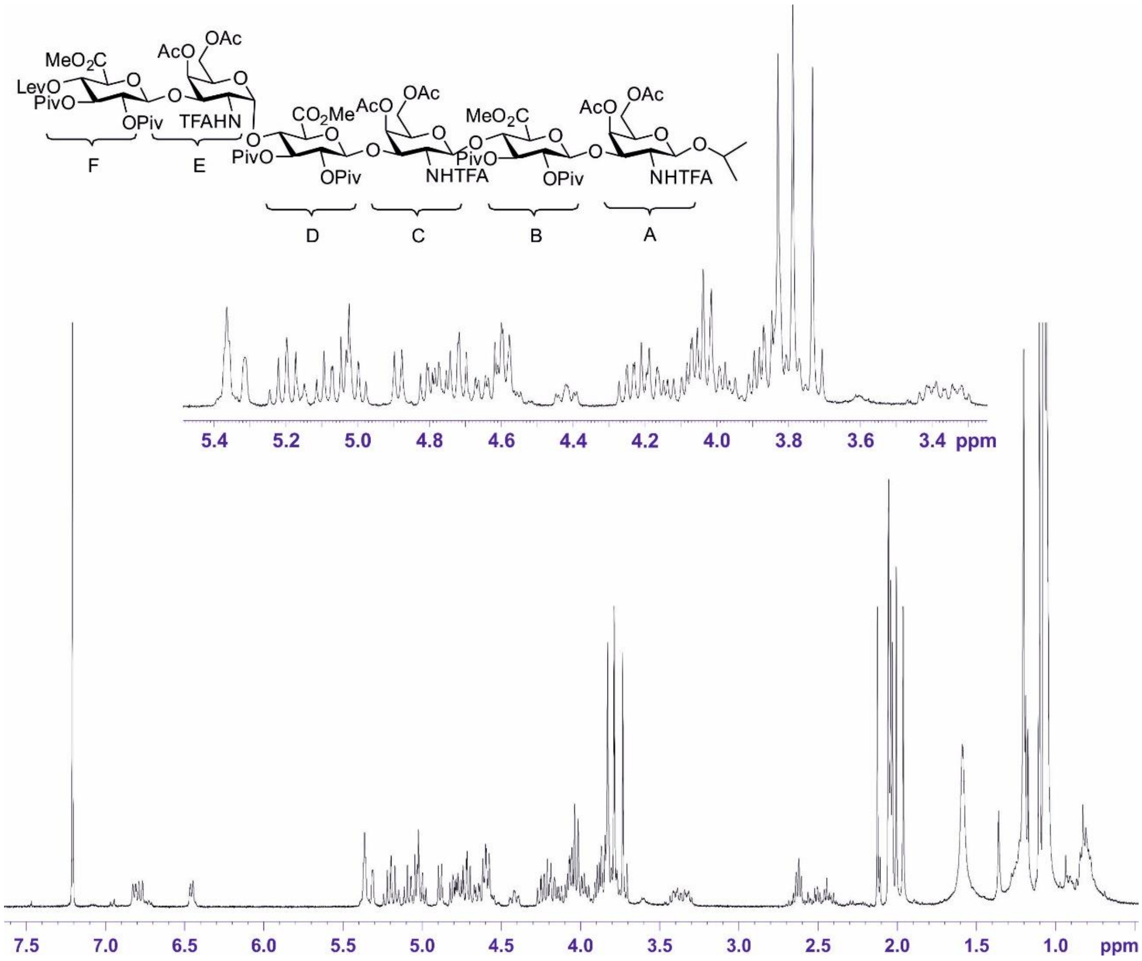

^{1}H NMR (400 MHz, $CDCl_3$) spectrum of **21α**.

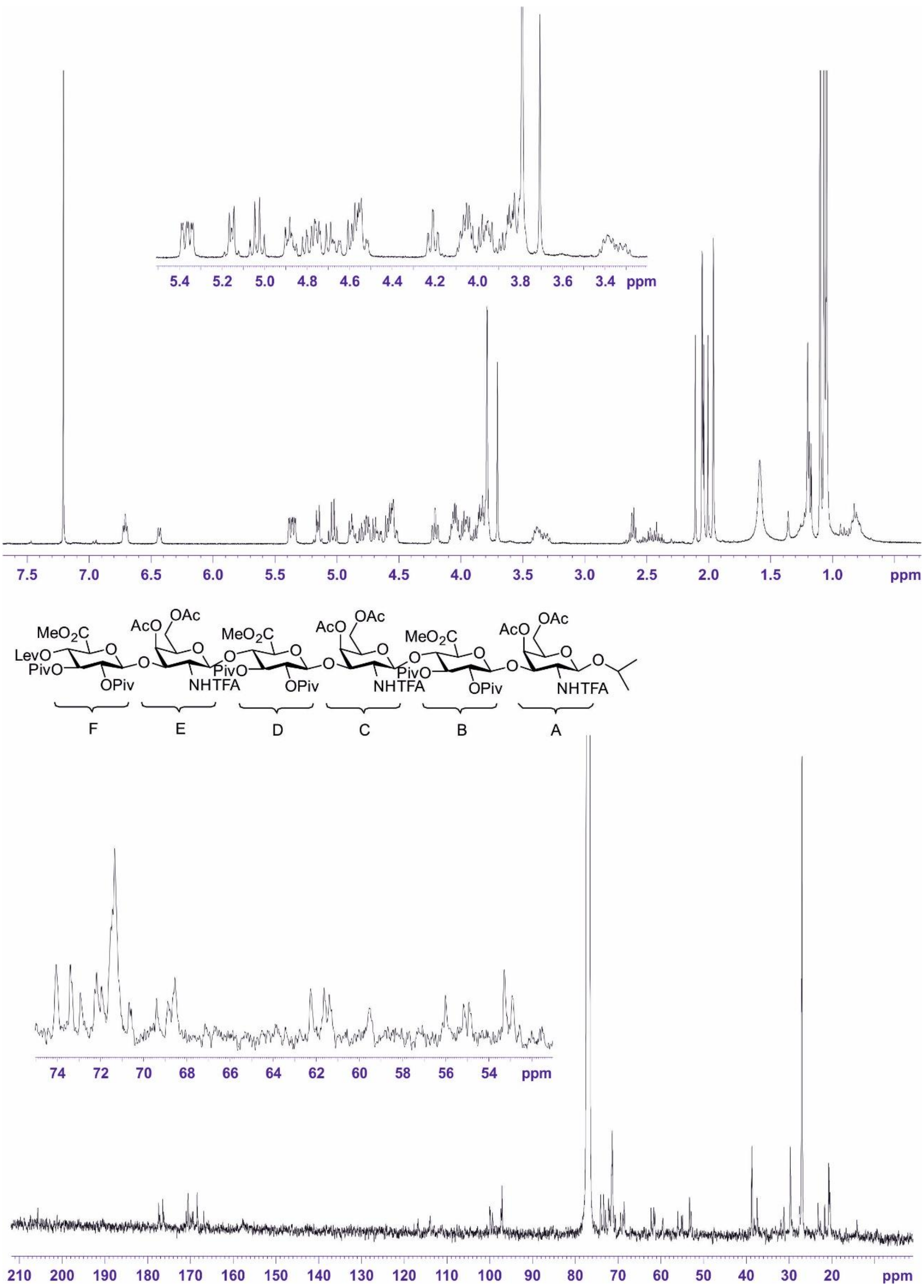

^{1}H and ^{13}C NMR (400 MHz, 100.6 MHz, $CDCl_3$) spectra of **21β**.

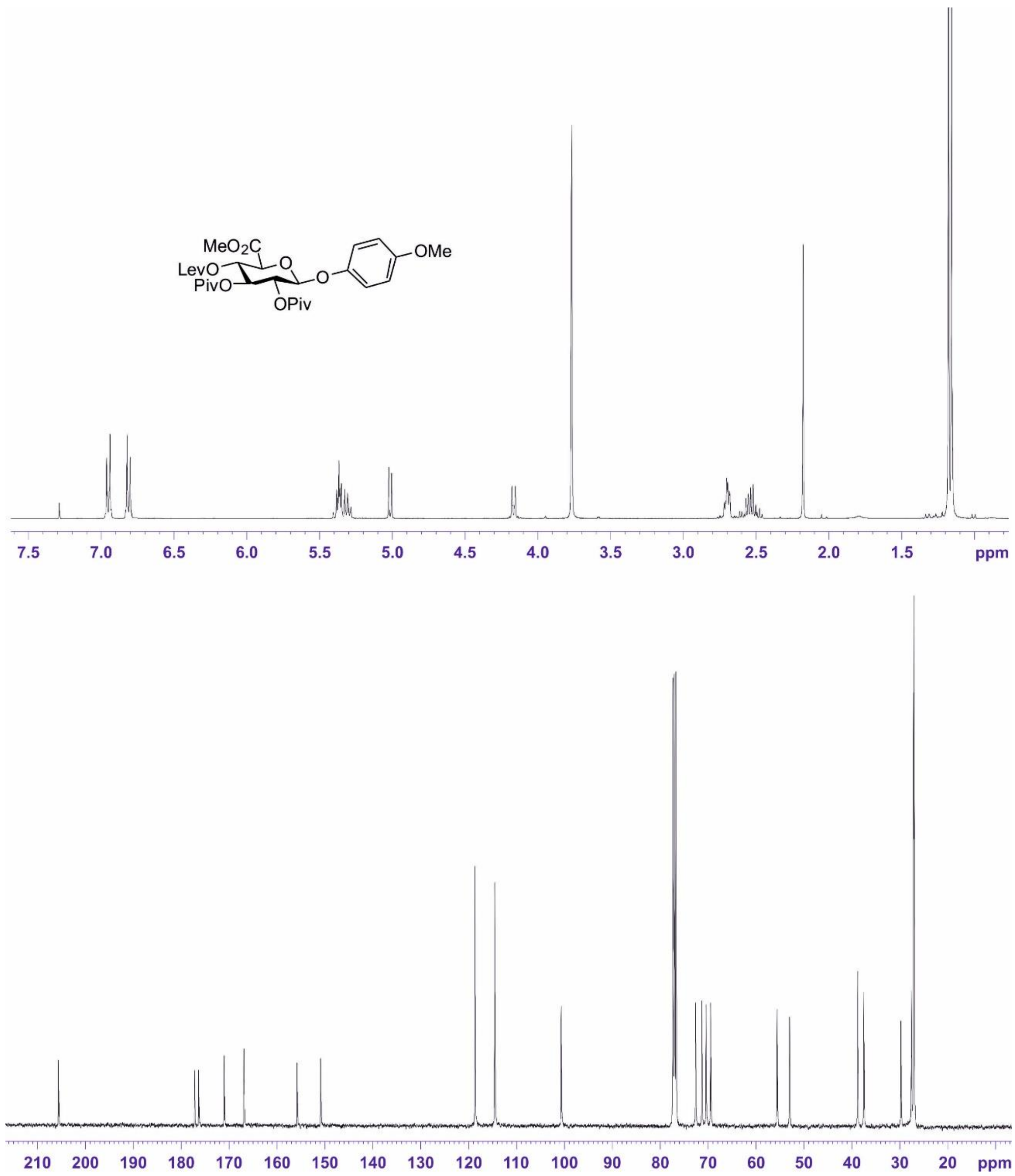

^{1}H and ^{13}C NMR (400 MHz, 100.6 MHz, $CDCl_3$) spectra of **25**.

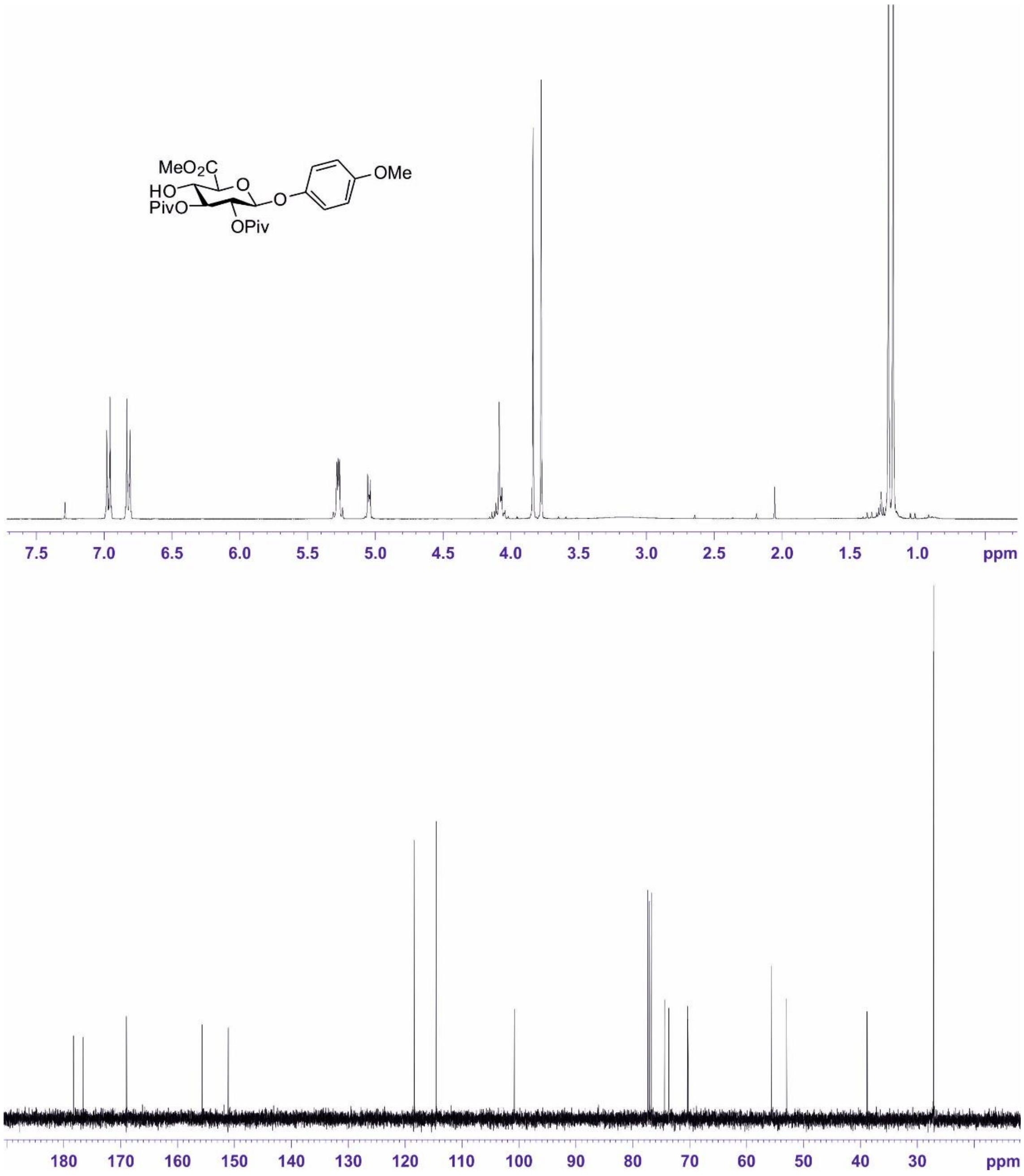

^{1}H and ^{13}C NMR (400 MHz, 100.6 MHz, $CDCl_3$) spectra of **26**.

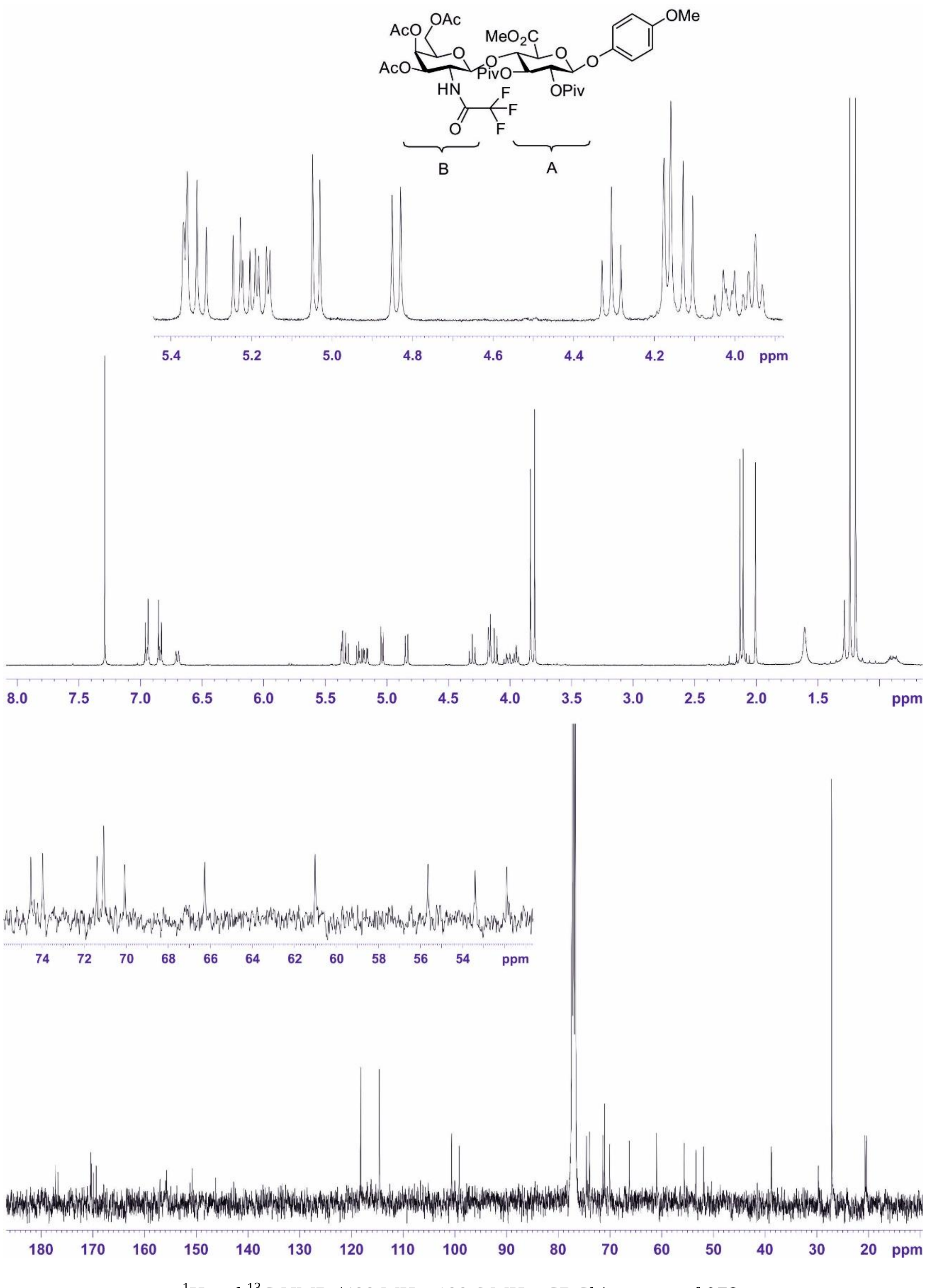

^{1}H and ^{13}C NMR (400 MHz, 100.6 MHz, $CDCl_3$) spectra of **27β**.

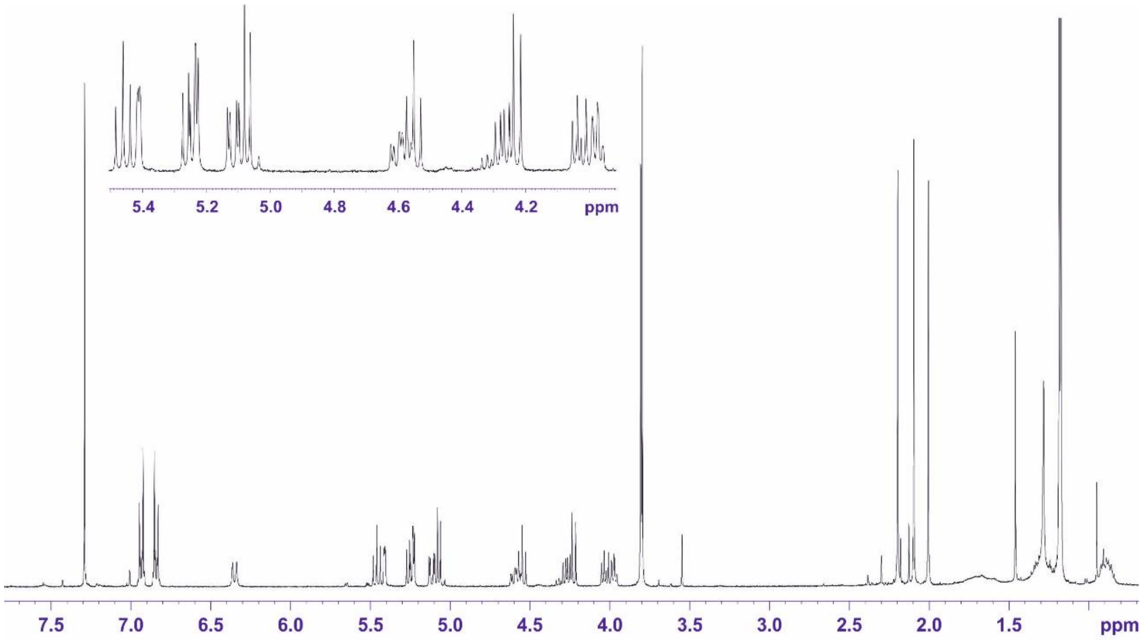

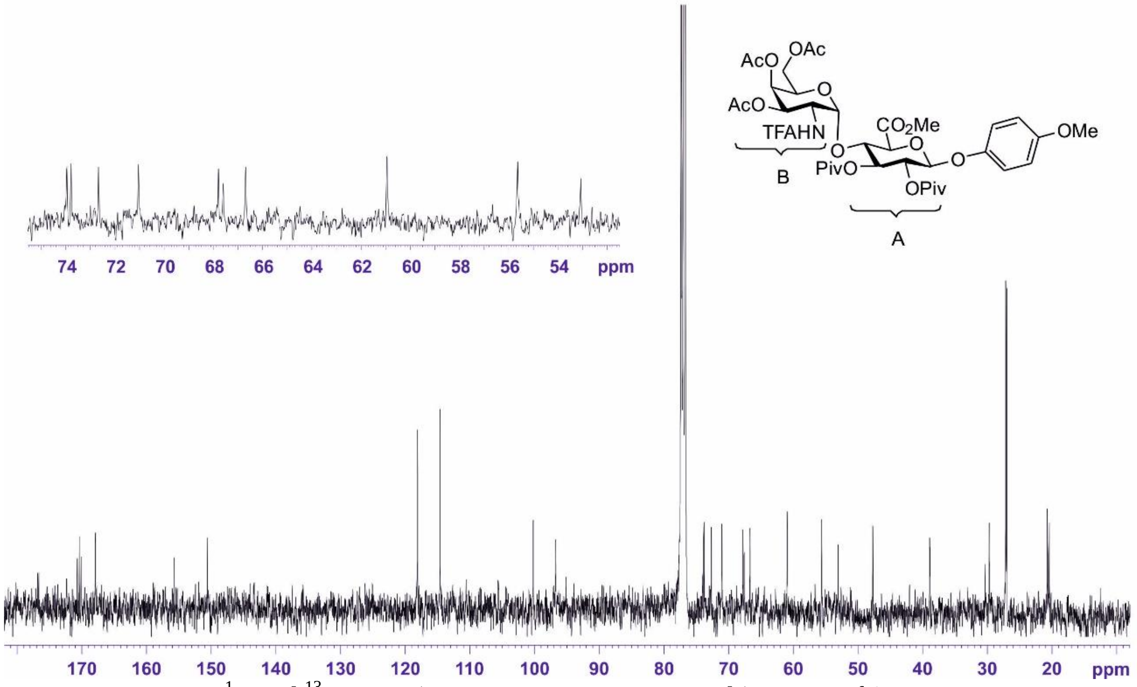

^{1}H and ^{13}C NMR (400 MHz, 100.6 MHz, $CDCl_3$) spectra of **27α**.

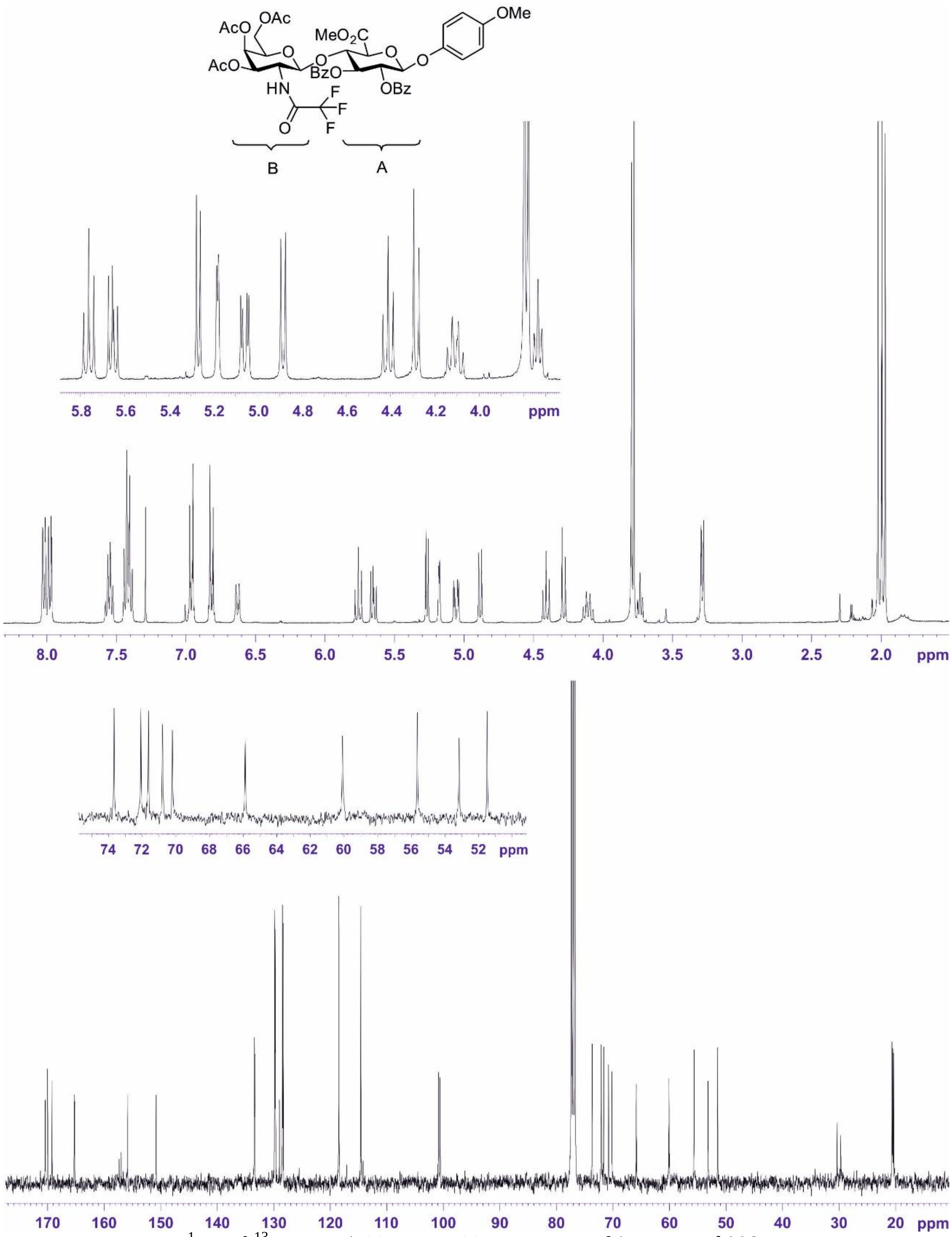

^{1}H and ^{13}C NMR (400 MHz, 100.6 MHz, $CDCl_3$) spectra of **29β**.
